# Single Cas9 nickase induced generation of *NRAMP1* knockin cattle with reduced off-target effects

**DOI:** 10.1186/s13059-016-1144-4

**Published:** 2017-02-01

**Authors:** Yuanpeng Gao, Haibo Wu, Yongsheng Wang, Xin Liu, Linlin Chen, Qian Li, Chenchen Cui, Xu Liu, Jingcheng Zhang, Yong Zhang

**Affiliations:** 1 College of Veterinary Medicine, Northwest A&F University, Yangling, 712100 Shaanxi China; 2 Key Laboratory of Animal Biotechnology, Ministry of Agriculture, Northwest A&F University, Yangling, 712100 Shaanxi China

**Keywords:** CRISPR-Cas9, Off-target, Homologous recombination, Chromatin immunoprecipitation sequencing (ChIP-seq), Nickase, Single-strand break, Tuberculosis

## Abstract

**Background:**

The CRISPR-Cas9 system is a widely utilized platform for transgenic animal production in various species, although its off-target effects should be addressed. Several applications of this tool have been proposed in model animals but remain insufficient for transgenic livestock production.

**Results:**

Here, we report the first application of single Cas9 nickase (Cas9n) to induce gene insertion at a selected locus in cattle. We identify the main binding sites of a catalytically inactive Cas9 (dCas9) protein in bovine fetal fibroblast cells (BFFs) with chromatin immunoprecipitation sequencing (ChIP-seq). Subsequently, we demonstrate that a single Cas9n-induced single-strand break can stimulate the insertion of the natural resistance-associated macrophage protein-1 (*NRAMP1*) gene with reduced, but still considerable, off-target effects. Through somatic cell nuclear transfer, we finally obtain transgenic cattle with increased resistance to tuberculosis.

**Conclusions:**

Our results contribute to the development of CRISPR-Cas9 system for agriculture applications.

**Electronic supplementary material:**

The online version of this article (doi:10.1186/s13059-016-1144-4) contains supplementary material, which is available to authorized users.

## Background

Homologous recombination (HR) has been widely applied to facilitate the exchange of DNA sequences between a targeted chromosomal locus and a homologous template containing the desired change [1–3]. HR is inefficient in many cell types and thus a site-specific double-strand break (DSB) is often introduced by site-specific homing endonucleases or artificial endonucleases, such as zinc finger and TALE nucleases. These endonucleases are introduced in the vicinity of the targeted genomic locus to stimulate subsequent DNA repair [4, 5]. Most recently, the type II bacterial clustered, regularly interspaced, short palindromic repeats (CRISPR)-associated protein 9 (Cas9) system has come to represent an efficient tool for the further advancement of gene targeting strategies [6–10] due to the simplicity of targeting any locus for cleavage with a single protein and a programmable single-guide RNA (sgRNA).

A site-specific DSB can efficiently stimulate HR by approximately 10,000-fold [11, 12]. Nonetheless, the competing non-homologous end-joining (NHEJ) pathway for DSB repair is often favored and frequently leads to small insertions/deletions (indels) or chromosomal rearrangements, particularly in mammalian cells [13, 14]. Thus, this repair pathway causes a major safety problem for gene targeting strategies, particularly for gene therapy and transgenic animal production [15, 16]. A number of studies have focused on enhancing the efficiency, specificity, and versatility of the CRISPR/Cas9 system [14, 17, 18], but reports of large functional gene insertions in mammalian genome editing are limited.

In the current study, chromatin immunoprecipitation (ChIP) was performed on a catalytically inactive double mutant (D10A and H840A) Cas9 (dCas9) [7, 19] for protein binding site detection in bovine fetal fibroblast cells (BFFs) and followed by sequencing (ChIP-seq). According to findings by others [6, 20], the single Cas9 nickase (Cas9n)-mediated single-strand break (SSB) has the potential to generally avoid the NHEJ repair pathway. Consequently, we determined the feasibility of using Cas9n-induced SSB to stimulate homology-directed repair (HDR) and therefore provided a safe alternative for gene insertion and transgenic animal generation. After a systematic selection of target sites and transgenic colonies, we successfully obtained nine exogenous natural resistance-associated macrophage protein-1 (*NRAMP1*) gene-inserted cows through the Cas9n strategy. Moreover, reduced off-target effects were detected both in the transgenic BFFs and cattle. We further demonstrated that Cas9n-mediated *NRAMP1* insertion provided the cattle with increased resistance to tuberculosis.

## Results

### Prediction and selection of the targeting locus and sgRNAs

**Fig. 1:**
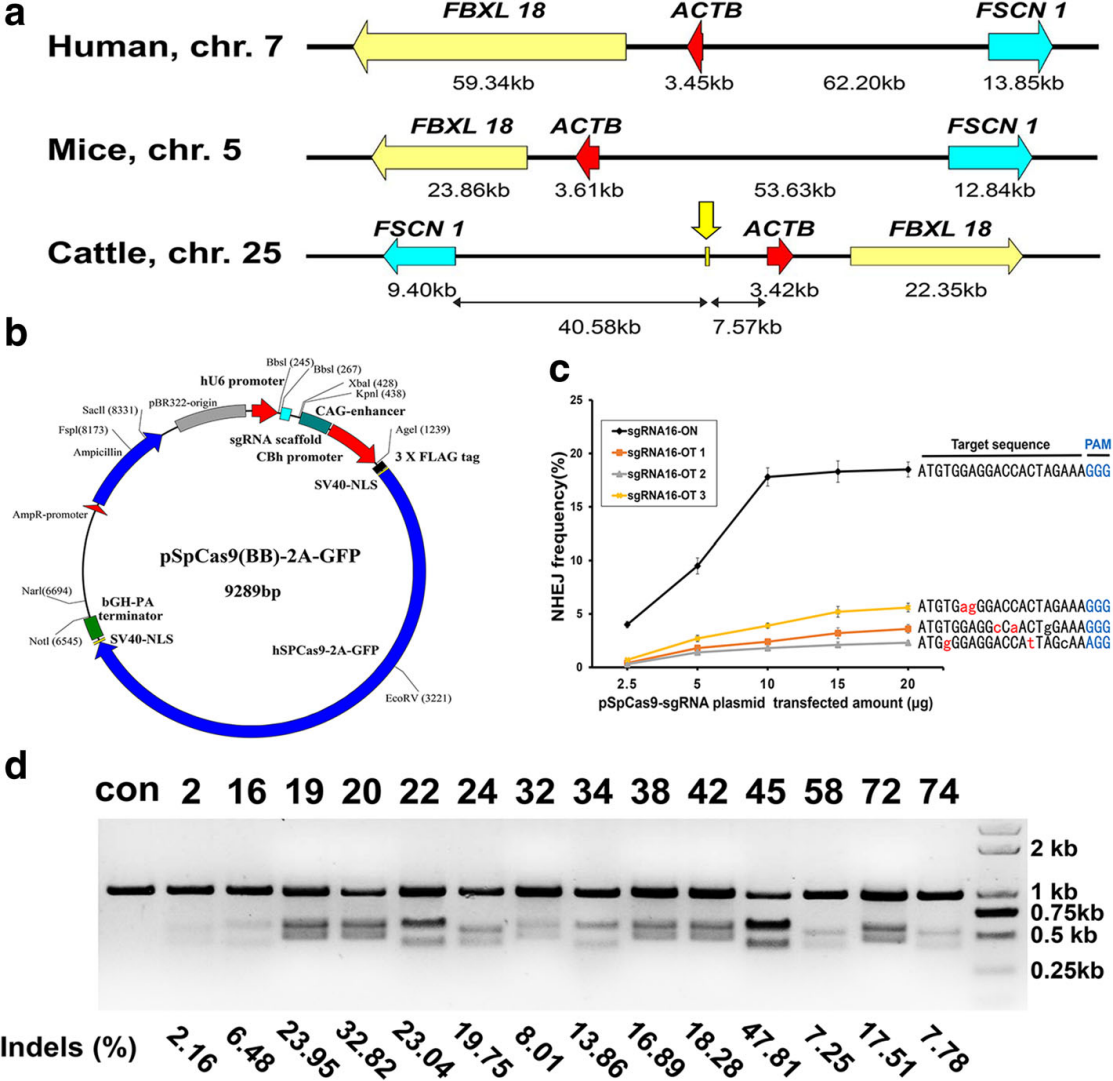
F-A locus and target site selection. **a** The molecular structures of the human, mouse, and cattle F-A loci were highly conserved. **b** *Schematic representation* of the plasmid encoding both hSpCas9 expression and sgRNA transcription. Different sgRNAs were cloned for the corresponding target sites through the two neighboring BbsI restriction enzyme sites. **c** Concentration-dependent cleavage activity and the amount of pSpCas9 DNA transfected. The three potential off-target sites were selected with Cas-OFFinder based on fewer than three mismatches and the NHEJ frequency was determined by a Surveyor nuclease assay. **d** Cleavage efficiencies of the Cas9 protein at the selected target sites. “Con” represents the DNA isolated from BFFs transfected with Cas9 only (without sgRNA). The degree of cleavage was quantified with Surveyor nuclease assays and ImageJ (http://imagej.net). The formula for estimating the indel rate is provided in the “Methods” section

Considering the potential synergistic effects of neighboring genes, we used an intergenic region between the fascin actin-bundling protein 1 gene (*FSCN1*) and the actin beta gene (*ACTB*) on chromosome 25 (positions 40,631,870–40,632,430; 561 bp long) as the potential gene target region (Additional file 1: Supplemental dataset S1). Housekeeping genes in the *FSCN1*-*ACTB* (F-A) locus (Fig. 1a) have relatively steady expression levels across various tissues and thus exogenous gene silencing resulting from chromatin inactivation might be avoided. We used the open-source website ZiFiT [21–23] (http://zifit.partners.org/ZiFiT/) and identified a total of 80 target sites in this locus. These sites either ended with NGG or started with CCN on the reverse strand (i.e. the protospacer adjacent motif (PAM)) [24] (Additional file 2: Supplemental dataset S2).

Regardless of the significant difference in the nucleotide number (varying from 5 to 12) of the seed region [14, 17, 25, 26], the PAM-proximal seed region of the guide sequence contributes more to the overall specificity of Cas9-mediated DNA cleavage and tolerates fewer mismatches than the PAM-distal sequences [19, 27, 28]. To implement the CRISPR/Cas9 system for cattle genome editing, we developed an evaluation model for the selection of sgRNAs. This evaluation model accounts for both the number of mismatches through an effective web-based off-target prediction tool termed Cas-OFFinder [29] (http://www.rgenome.net/cas-offinder) and the flexible relevance between the mismatch location and the seed region (Additional file 2: Supplemental dataset S2). As an alternative to this evaluation model, we also presented most of the predicted off-targets in detail. For example, the visualized possible off-target-enriched zones in the hypothetical 12-, 8-, and 5-nucleotide seed regions are presented as well as the copy number of the seed + NGG sequence at the genome level. The detailed data are available in Additional file 2: Supplemental dataset S2 and Additional file 3: Figure S1. Finally, we selected 14 potential target sites for subsequent efficiency detection.

### Construction of Cas9 target plasmids and cleavage efficiency detection

To avoid interference from the relative ratio discrepancy of the hspCas9 protein and sgRNA, we chose the widely used all-in-one pSpCas9 (BB)-2A-GFP plasmid (PX458, Addgene plasmid #48138) [30] as the basic target plasmid (Fig. 1b). The primers used to clone each sgRNA are available in Additional file 3: Table S1. The off-target cleavage of Cas9 is highly sensitive to the transfected amount of plasmid [25, 27, 30]; hence, we randomly selected the target site 16 and carefully titrated the amount of the transfected reformed plasmid pSpCas9-sgRNA16. When the transfected DNA was increased to 10 μg, the frequency of disruption at the target site reached a peak, whereas the disruptions of three putative off-target sites still occurred at relatively low levels (Fig. 1c). Thus, this concentration was optimal.

Subsequently, the DNA cleavage efficiencies in the BFFs at all 14 target sites were determined via Surveyor nuclease assays [31]. The results indicated that the majority (10/14) of the cleavage efficiencies (*f*_*cut*_) were in the range of 13.97–40.77% and the corresponding indel rate was in the range of 7.25–23.04% (Fig. 1d and Additional file 3: Table S2). Notably, the indel percentages in sgRNA20 and sgRNA45 reached 32.82% and 47.18%, respectively. We further amplified the genomic target loci from the pSpCas9-sgRNA22- and pSpCas9-sgRNA45-transfected BFFs via polymerase chain reaction (PCR) and TA cloning. Subsequently, totals of 182 and 179 transformed *Escherichia coli* bacterial colonies were randomly selected for Sanger sequencing to confirm the presence of Cas9 nuclease-induced indels at the targeted locus and these colonies had indel rates of 21.43% and 41.90%, respectively (representative sequences are provided in Additional file 3: Figure S2). These results agreed with those from the Surveyor nuclease assays. Thus, these detection efforts provided target sites with noticeably different cutting efficiencies for subsequent experiments.

### Genome-wide binding of the sgRNA-dCas9 in BFFs

**Fig. 2:**
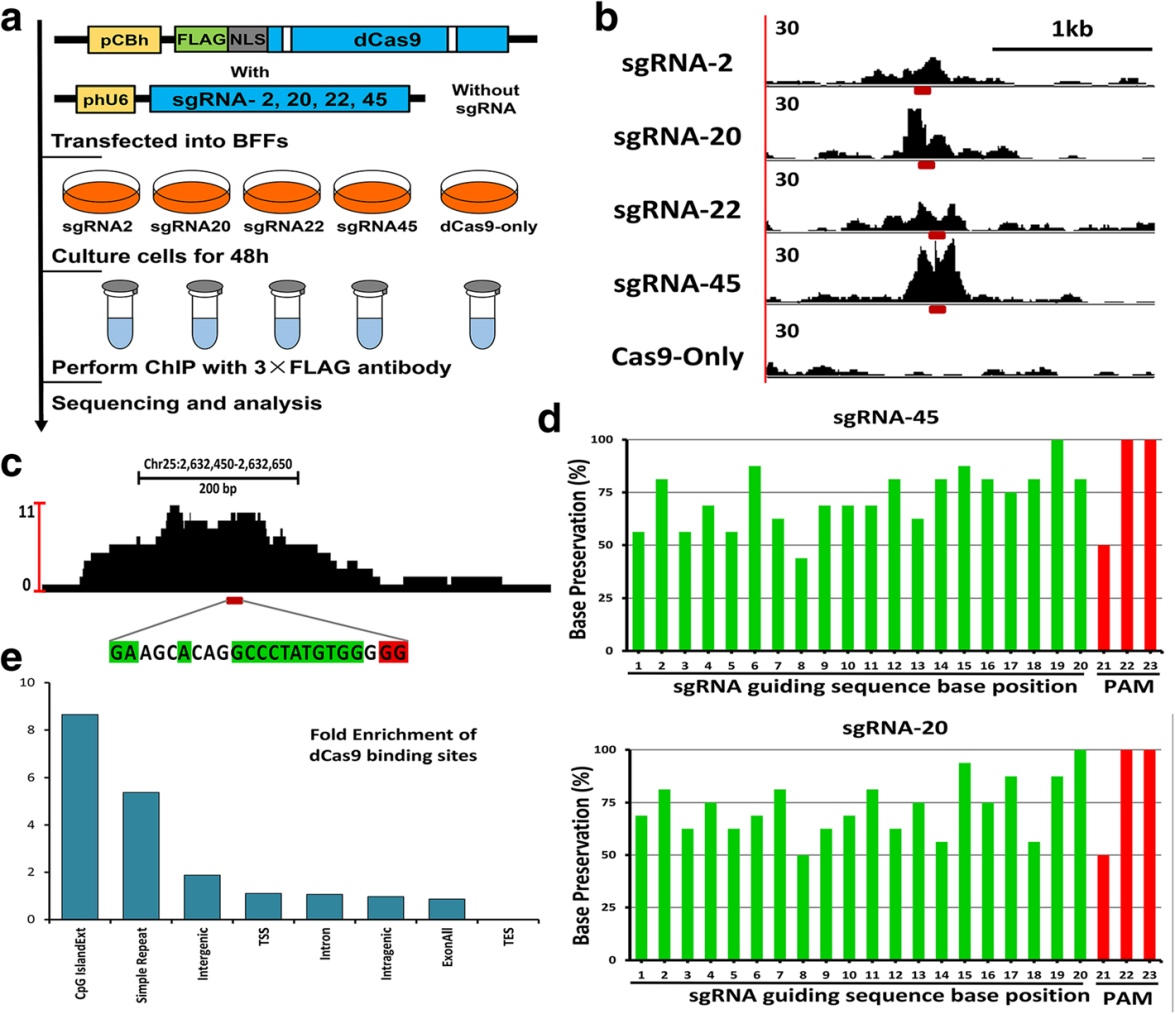
Process and characteristics of dCas9 binding in BFFs. **a** *Schematic representation* of the dCas9 ChIP-seq approach applied to the BFFs. The four 3 × FLAG-tagged Cas9-encoding plasmids with different sgRNAs were considered different experimental groups (*left*) and the same plasmid but without sgRNA was used for the dCas9-only control (*right*). **b** Visualization of the ChIP-seq peaks (normalized read counts) revealed that they were localized around four on-target sites and the control. The *red* dashes under each peak indicate the designed target sites. **c** Visualization of the ChIP-seq peak of one typical off-target region. The location of this region is shown above the peak. Bases matching the sgRNA guiding sequences and PAM sequences at the off-target sites are highlighted in *green* and *red*, respectively. **d** Percentages of preserved bases at the main off-target sites compared with the guiding sequences of sgRNA45 (*above*) and sgRNA 20 (*below*). **e** Performance of dCas9 in binding to the chromatin structure of the off-target binding site. “TSS” represents the 1 kb region centered on the transcription start site region; “TES” represents the same range of the transcription end site

To gain insight into the targeting specificity and the extent of the off-target effects of CRISPR/Cas9 system in the bovine genome, we performed ChIP-seq for dCas9 protein binding site detection in BFFs. A total of four target sites (2, 20, 22, and 45) with typical cleavage efficiencies were used and the corresponding sgRNAs were cloned into the 3 × FLAG-tagged dCas9 expression vector (Fig. 1b). After the transfection of these plasmids into the BFFs for 48 h, ChIP was performed following immunoprecipitated dCas9-associated DNA sequencing on a single lane of the Illumina HiSeq 2500 platform (Fig. 2a). The clean reads were then aligned to the *Bos taurus* genome sequence (version: Btau_4.6.1) using the BWA program [32].

Visually strong peaks (200–500 bp) were observed at the intended target sites of each sgRNA (Fig. 2b) and large numbers of common peaks were also detected among the different sgRNAs (including the dCas9-only group). These observations are consistent with those of previous studies [26, 28] that have identified such peaks as false positives due to sequencing biases, repeat-rich sequences, and GG/CC-rich motifs in a particular chromosomal region. To focus on the dCas9-binding sites that are specific for particular sgRNAs, we applied pair-wise peak calling with model-based analysis of ChIP-seq (MACS) [33] between each sgRNA sample and the other groups (including the dCas9-only control). Hence, only the enriched peaks in an individual group were retained for the remaining analyses.

**Fig. 3:**
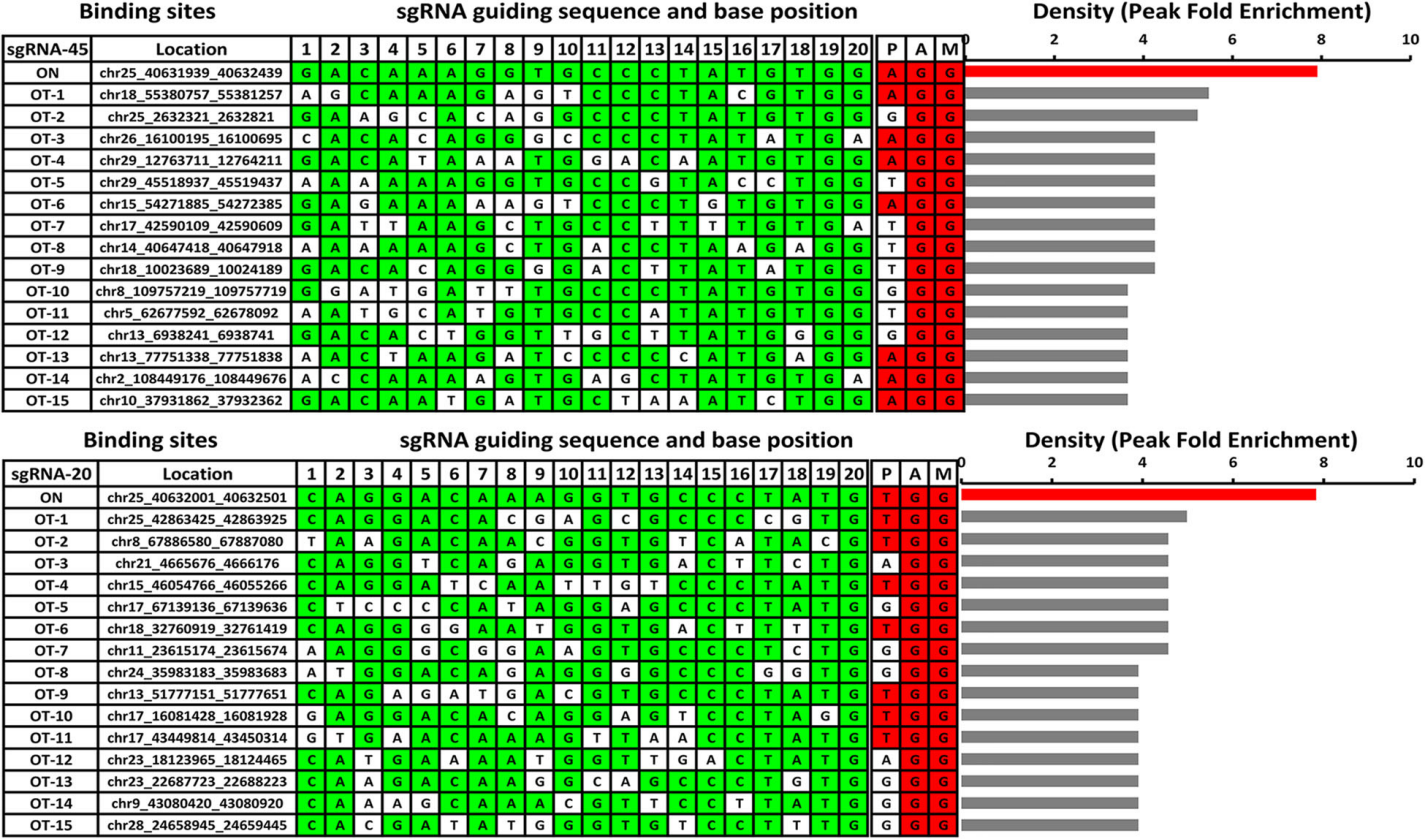
Off-target sites of sgRNA 45 and 20 with the top 15 ChIP-seq binding densities. All of the off-target sites were computationally identified with 20 bp sequences. These sites ended with PAM and aligned most effectively with the sgRNA guiding sequences in each peak. OT indicates off-target; all of the off-target sites in the same group were ranked according to the ChIP-seq binding densities (peak fold enrichments) as illustrated in the *bar graphs* on the right. At the off-target sites, bases matching the sgRNA guiding sequence and the PAM sequence are highlighted in *green* and *red*, respectively. Similar results for sgRNA 2 and 20 are available in Additional file 3: Figure S3

Because both the on-target (Fig. 2b) and potential off-target sites (Fig. 2c) occurred primarily in the centers of the binding peaks, we computationally identified the 20-bp-long potential recognition sequences. These sites both ended with PAM and aligned most effectively with the sgRNA-guiding sequence. Subsequently, the top 15 off-target sites of each sgRNA group were ranked according to the ChIP-seq binding density (peak fold enrichment) as illustrated in Fig. 3 and Additional file 3: Figure S3. The greatest density of Cas9 binding was located at the on-target site in all four sgRNA groups, but many of the peaks at the off-target sites exhibited high signal intensity. This observation demonstrated the existence of substantial off-target sites for the dCas9-sgRNA complexes that bound in the BFFs and might address the necessity to confirm the actual off-target effects to avoid unexpected gene modifications. Regarding sgRNA2, some of the off-target peaks were greater than the on-target peak (Additional file 3: Figure S3), which might have resulted from the additive effect of multiple potential binding sites in the same peak [28].

### Characteristics of dCas9 binding in BFFs

Additionally, we compared the base compositions of the main off-target sites with the on-target sequence (Fig. 2d and Additional file 3: Figure S4) to assess the presence and composition of the “seed sequence.” This sequence was defined as the conserved sequence next to the PAM and was less tolerant of mismatches in the sgRNA. As expected, a higher base preservation rate was observed in the PAM-proximal position than in the PAM-distal region. Using sgRNA 45 (n = 3562 sites) as an illustration, eight of the ten PAM-proximal bases were preserved in 75% of the off-target sites and only two bases were conserved in the PAM-distal ten bases. Similar but smaller magnitude trends were observed for the sgRNAs 20 and 22. Nevertheless, no obvious base preservation was observed in the proximal or distal regions of sgRNA 2. In accordance with the conclusion from previous studies, we confirmed that all positions were preserved in the sgRNA-guiding sequence, but the PAM-proximal seed region tolerated fewer mismatches and contributed more to the binding specificity than the remaining regions in the BFFs.

To detect the performance of dCas9 binding to the chromatin structure, the potential off-target sites were analyzed (Fig. 2e) based on the UCSC genome annotation. Considering the large number of common peaks in the repeat-rich sequences and the GG/CC-rich motifs (described above), we also considered the strong enrichment at CpG islands and simple repeats as false positives along with the results from the imperfect pair-wise peak calling with MACS. Interestingly, an approximately 40-fold enrichment was observed in the microsatellite sequence motifs, but this enrichment was primarily eliminated after the pair-wise peak calling process. The greater enrichment values in the transcription start site (TSS) regions compared with the transcription end site (TES) regions has previously been reported [28]. This result suggested that the chromatin structure might influence dCas9 binding. The substantial enrichment of off-target binding in the intergenic regions of the BFFs might also support the target locus selection of this study.

The ChIP enrichment level correlates well with the DNA binding intensity and the frequency of interaction [34], but the correlation between Cas9 binding and DNA cleavage remains to be tested. To corroborate the practical indel frequencies induced by the Cas9 nuclease at the ChIP-seq designated off-target sites, we again transfected reformed plasmids encoding wild-type (WT) Cas9 or Cas9n with sgRNAs 2, 20, 22, and 45 into BFFs. Subsequently, the peak-covered sequences were amplified by PCR and deep sequenced (the primers are provided in Additional file 3: Table S3). The plasmid encoding WT Cas9 without sgRNA was also used as a control at the same sites. Detailed calculations of the indel rates at the 60 main off-target sites and four on-target sites are provided in Additional file 3: Table S4. As expected, high indel rates were observed at the four on-target sites in the WT Cas9 group. Moreover, 13 of the 60 off-target sites (21.7%) exhibited significantly greater indel rates than the control (*P* < 0.05, Fisher’s exact test) and four of these sites belonged to the sgRNAs 20 and 45.

Similar indel frequencies were observed in the single Cas9n group at nearly all sites compared with the control. In addition, the single Cas9n group has low indel rates at the target site compared with the WT Cas9 group (Additional file 3: Table S4). Based on the substantial indel frequency detection at the off-target sites, we hypothesized that single Cas9n-mediated SSB can generally avoid the NHEJ repair pathway.

### Single Cas9n succeeded in inducing desired HDR with considerable efficiency

**Fig. 4:**
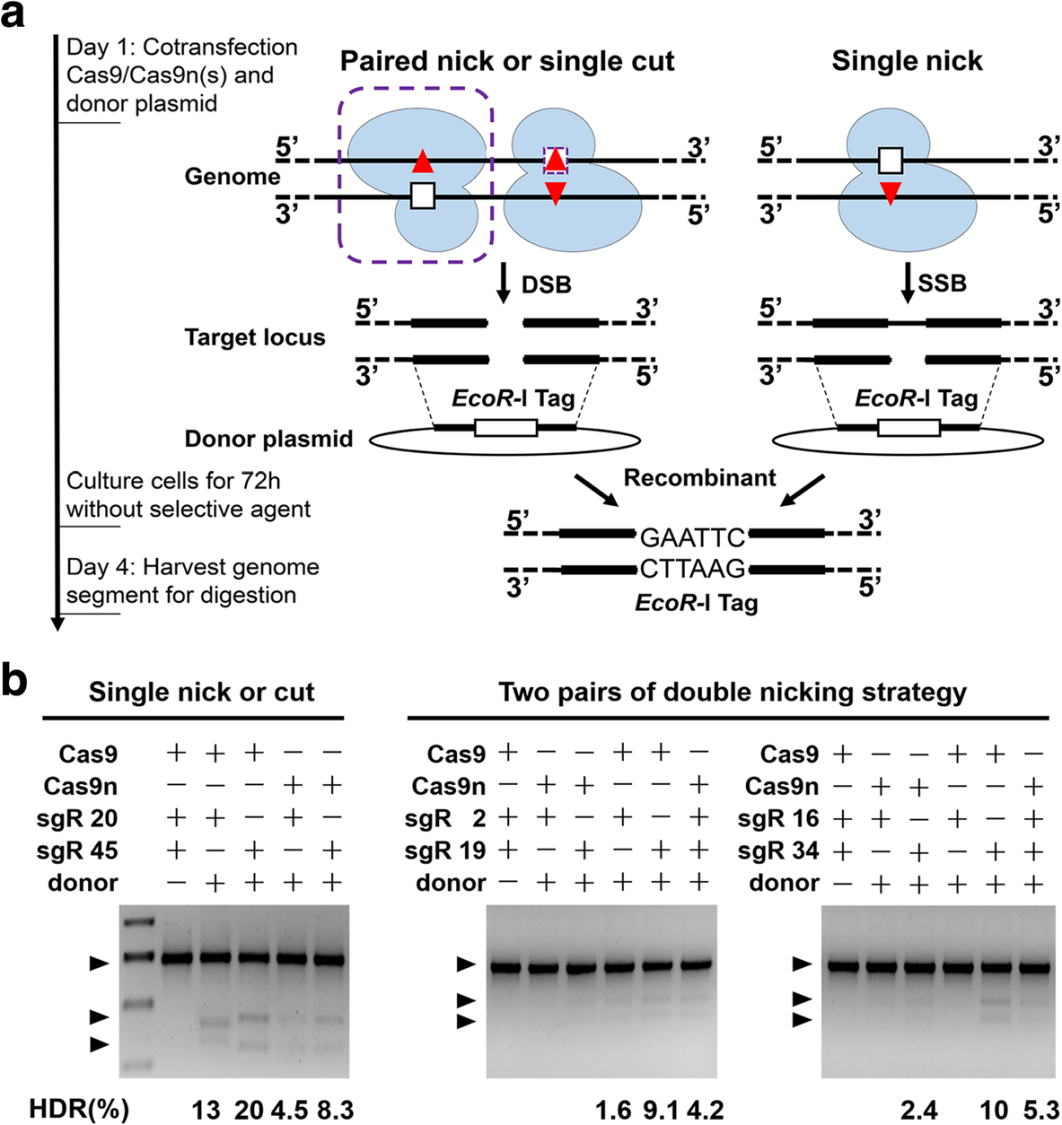
Single Cas9n stimulated HDR at the F-A locus. **a** Experiential *outline* and *schematic* of the HDR process. The designed donor plasmid was recombined with the genome through single Cas9-induced DSB (*left*, without the *purple frames*), paired Cas9n-induced DSB (*left*, with the *purple frames*), and single Cas9n-induced SSB (*right*). **b** HDR frequency measurements of the different targeting strategies based on restriction enzyme tag integration. The specific numbers below each lane were calculated with the relative intensities of digested bands and the undigested band

To further evaluate the Cas9n-induced HDR recombination efficiency, we selected target sites 20, 45, and two paired target sites, i.e. 2 + 19 and 16 + 34, for the subsequent experiments. In the double-nicking strategy, two highly active sgRNAs in sufficient proximity (selected through ZiFit) are required to design an efficient nickase pair [9, 20]. We designed two donor plasmids as HDR repair templates in which an EcoR-I restriction site was flanked by the paired homology arms for the above four groups (Fig. 4a).

WT Cas9, single Cas9n, or paired Cas9ns recombinant plasmid(s) were respectively co-transfected with the donor plasmid into the BFFs. The genomic DNA was extracted for targeted-locus PCR-amplification 72 h later and the subsequent EcoR-I tag digestion assays were showed in Fig. 4b. Compared with the WT Cas9 groups, all of the single Cas9n-induced HDRs occurred at perceptibly low frequencies (60–80% reductions), which indicated a decrease in the HDR efficiency at the same target site (Fig. 4b). We also observed that the HDR recombination rates of the single Cas9n at the target sites 20 (4.5%) and 45 (8.3%) were comparable to those of the paired Cas9n-2 + 19 (4.2%) and Cas9n-16 + 34 (5.3%) group.

Taken together, these results indicated that single Cas9n-mediated SSB can successfully induce HDR recombination at a systematically selected target site with reduced but still considerable efficiency.

### Selection of the transgene

To address one of the major diseases in cattle and minimize immunological disruptions, we focused on endogenous functional genes associated with innate immunity for the transgene. The first identified tuberculosis-susceptible candidate [35], i.e. the *NRAMP1* gene, which has been renamed as the solute carrier family 11A member 1 gene (*SLC11A1*), is associated with innate resistance to intracellular pathogens, such as *Mycobacterium*, *Leishmania*, *Salmonella*, and *Brucella* [36–38]. The expression of *NRAMP1*, which exclusively occurs in macrophages and other dedicated phagocytes, is upregulated by cytokines and induces the production of nitric oxide (NO) in addition to other pro-inflammatory responses [39].

In the present work, the bovine *NRAMP1* cDNA sequence was amplified from the peripheral blood of Holstein–Friesian cows (the sequence is available in Additional file 1: Supplemental Dataset S1). This sequence displayed 99% identity with the published sequence (NCBI Gene ID: 282470), i.e. there were only two nucleotide variations. The only non-synonymous nucleotide substitution occurred in codon 49 (ACA to GCA) and led to a threonine-to-alanine residue change that represents a functionally irrelevant single nucleotide polymorphism (SNP) based on previous work [40]. Moreover, the overexpression of bovine *NRAMP1* can reduce the multiplication of *Mycobacterium bovis* (*M. bovis*) in the murine RAW264.7 macrophage cell line after infection via a cfu test (Additional file 3: Figure S5, 96 h: 325% ± 21% versus 136% ± 24%, *P* = 0.0017). This cell line carries the *NRAMP1* G169D allele and therefore displays a functional *NRAMP1* deficiency [36]. Consequently, we decided to add a *NRAMP1* gene to the specific locus above in the bovine genome.

### Single Cas9n mediates a site-specific *NRAMP1* insertion in BFFs

**Fig. 5:**
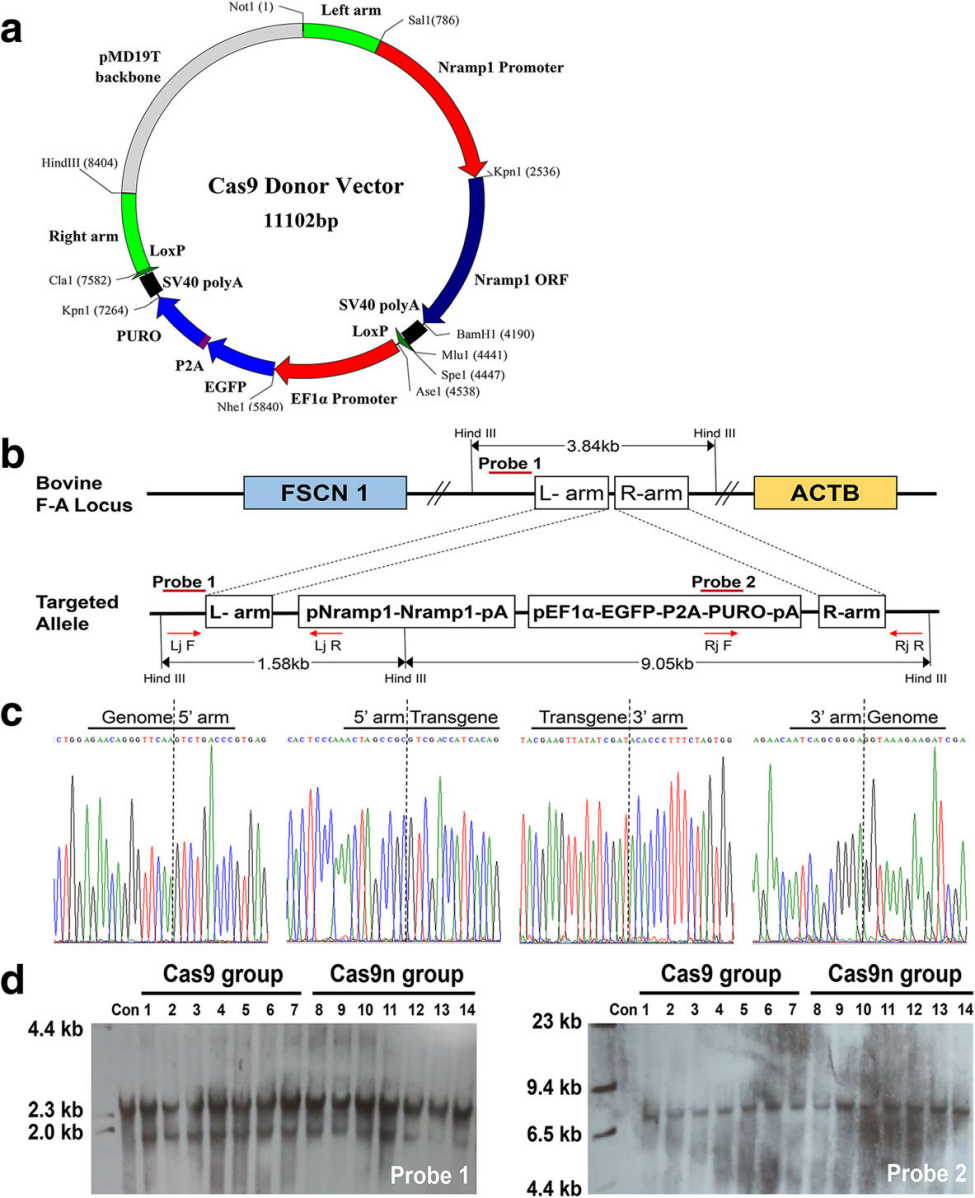
Insertion and selection of the *NRAMP1* transgenic colony using single Cas9 or Cas9n. **a** *Schematic representation* of the gene-targeting donor vector. **b** *Schematic overview* of the screening of the individual colonies. Lj F and Rj R were the primers for the regions outside the homologous arms, and Lj R and Rj F were the primers for the donor vector region. Southern blot probes are shown as *red lines* and Hind III digestion was used in the southern blot analysis. **c** Sanger sequencing confirming the precise insertion of the exogenous DNA. **d** Southern blot analysis of the donor cells used for SCNT. Non-transfected BFFs were used for negative controls. A 1.58 kb band resulting from the targeted insertion of the *NRAMP1* cassette was detected in addition to the 3.84 kb band from the endogenous F-A locus allele when probe 1 was used. A 9.05 kb targeted band was also detected with probe 2

The gene-targeting donor vectors p*NRAMP1*-eGFP-P2A-Puro-1/2 (Fig. 5a) were constructed with the same element but different homology arms for the corresponding target sites. The sequences are provided in Additional file 1: Supplemental Dataset S1. We cloned the Holstein *NRAMP1* 5′ flanking region (−1748 to +73) as an original promoter sequence to direct *NRAMP1* expression at a level comparable to that of the natural gene activity. Because the selected markers are driven by an EF1α promoter, the *puromycin* gene and enhanced green fluorescent protein were fused to the porcine teschovirus-1 2A (P2A) peptide sequence [41]. Subsequently, two LoxPs in the same orientation were added to each side to remove the markers when required [42]. We transfected the donor vector, along with an expression plasmid encoding WT cas9, single cas9n, or paired cas9n, to introduce a DSB/SSB and the further insertion of *NRAMP1* at four target sites (20, 45, 2 + 19, and 16 + 34) in the BFFs (Additional file 3: Table S5). Early-passage (P2 or P3) primary BFFs were obtained from female Holstein–Friesian dairy cows and used for targeting.

Following 8–10 days of *puromycin* (2 μg/mL) selection, firmly transfected colonies were trypsinized and screened by 5′ junction (1.58 kb) and 3′ junction (1.98 kb) PCR. The product should contain both the insert and internal genome sequences to confirm that stable genetic cell modification occurred at the intended specific site (Fig. 5b, c, and Additional file 3: Table S5). Representative PCR results are provided in Additional file 3: Figure S6. Statistical tests revealed that 34.55% (57/165) of the *puromycin*-resistant colonies contained cells that were correctly targeted at site 45 by WT Cas9, whereas the positive recombination rate for single Cas9n at the same site was 18.11% (69/381). The weakened positive recombination rate agreed with the HDR efficiency detected above (Fig. 4b). Based on our previous studies [43, 44], a positive recombination rate of 18.11% should be sufficient for gene editing.

To further evaluate the ploidy of transgenic cassette in the clones that passed two rounds of junction PCR, we designed two probes for southern blot analyses (Fig. 5d). The 1.58 kb band (probe 1) resulting from the *NRAMP1* cassette insertion and the single 9.05 kb targeted band (probe 2) suggested site-specific integration. The 3.84 kb band (probe 1) from the endogenous F-A locus allele suggested that the tested colonies were heterozygous and exhibited one normal chromosome. Constitution of the positive cell colonies generated by different types of Cas9 at the target site 45 were available in Additional file 3: Table S6. After confirming the successful insertion, karyotype analysis of each heterozygous colony was performed (a typical and representative karyotype is illustrated in Additional file 3: Figure S7).

**Fig. 6:**
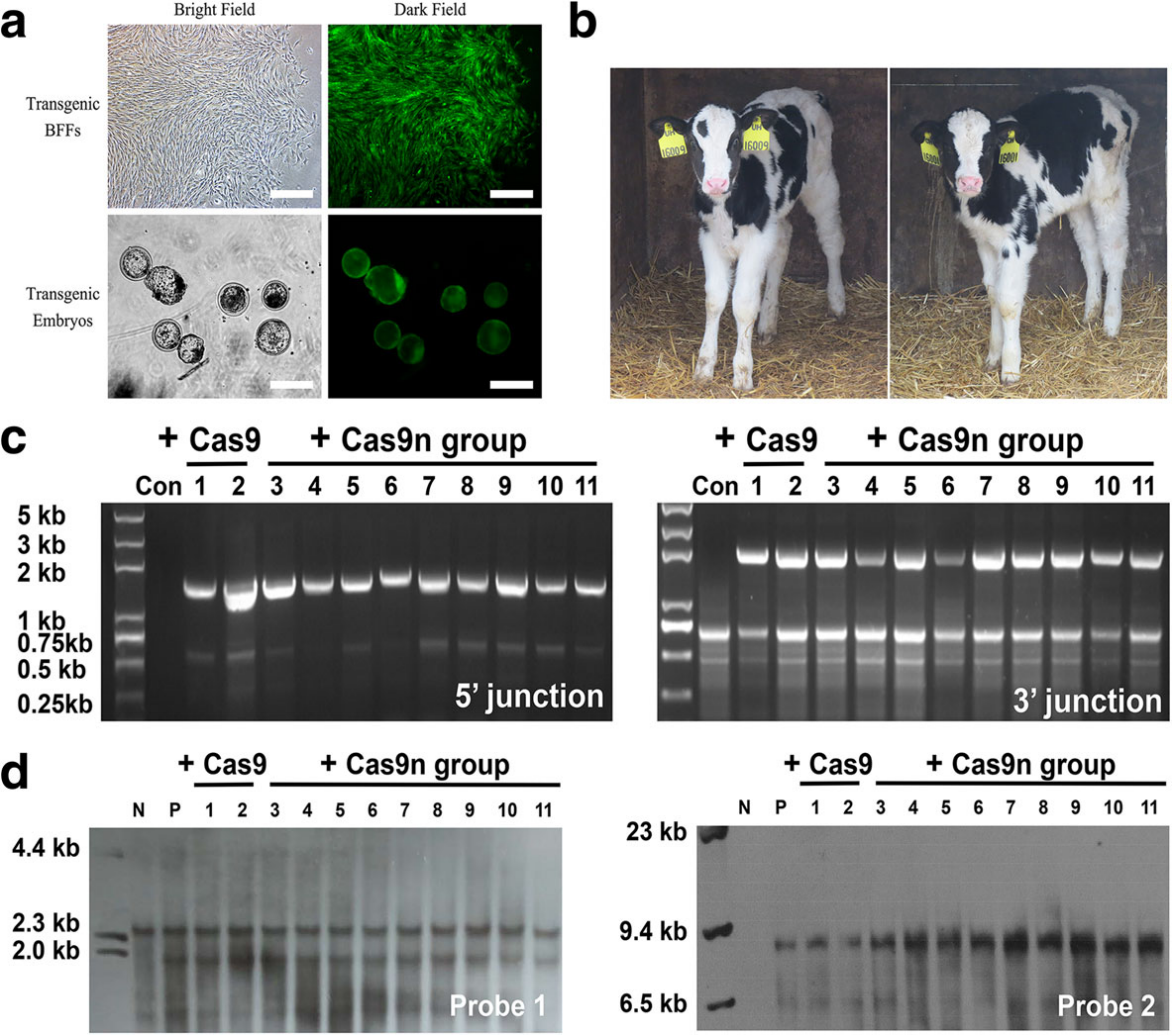
Assessment of the transgenic cattle. **a** Transgenic colony after positive drug selection and transgenic embryos from SCNT under a fluorescence stereomicroscope. **b** Photographs of one-month-old calves that carried the *NRAMP1* insertion. **c** The 5′ (*left*, 1.58 kb) and 3′ junction (*right*, 1.98 kb) PCR analyses confirming the site-specific targeting in the transgenic cattle. “Con” represents the control normal cattle. The templates for PCR were genomic DNA samples that were extracted from the peripheral blood of cattle. **d** Southern blot analysis of the genomic DNA extracted from transgenic cattle. “N” represents the negative control normal cattle; “P” represents the positive transgenic BFFs

Finally, a total of 113 heterozygous colonies that were edited at site 45 and exhibited normal karyotypes, compact spindle-like cell morphologies, and rapid growth (Fig. 6a) were available for somatic cell nuclear transfer (SCNT).

### Nuclear transfer for generating *NRAMP1* knockin cattle

We randomly picked eight transgenic colonies (evenly split between the WT Cas9 and single Cas9n groups) as donor cells to produce cloned transgenic cows according to the above selection criteria. A total of 2385 and 2434 embryos were successfully reconstructed in vitro (Fig. 6a) for each group. Among these, 525 (22.0%) and 753 (30.9%) of the embryos from the WT Cas9 and single Cas9n groups developed to the blastocyst stage and were transferred into the oviducts of 173 and 248 recipient heifers, respectively. As indicated in Table 1, both the blastocyst formation and pregnancy rates of the Cas9n group were greater than those of the WT Cas9 group, which may be attributable to the less mutagenic NHEJ and low toxicity. Finally, a total of 20 calves (16 from the Cas9n group) were born and 11 calves (nine from the Cas9n group) survived longer than three months (Fig. 6b).

**Table 1:** Summary of nuclear transfer results from gene-targeted bovine fetal fibroblast cells

Nuclear donor	Cas9 nuclease					Cas9 nickase				
	Typical colonies suitable for SCNT				Total	Typical colonies suitable for SCNT				Total
Cell clone	45–57	45–86	45–136	45–159	-	45–113	45–187	45–243	45–351	-
Embryos obtained	632	578	644	531	2385	568	674	527	665	2434
Blastocysts (%)	137 (21.7)	143 (24.7)	126 (19.6)	119 (22.4)	525 (22.0)	163 (28.7)	210 (31.2)	157 (29.8)	223 (33.5)	753 (30.9)
Recipients	46	49	41	37	173	54	68	51	75	248
Pregnancies (%)	6 (13.0)	9 (18.4)	2 (4.9)	5 (13.5)	22 (12.7)	9 (16.7)	18 (26.5)	11 (21.6)	23 (30.7)	61 (24.6)
Calves at birth	1	2	0	1	4	2	5	3	6	16
Survived for 3 months	0	1	0	1	2	1	3	2	3	9

Junction PCR and southern blot analyses were again performed on the remaining 11 living calves (Fig. 6c and d). The results demonstrated that the exogenous *NRAMP1* was precisely integrated at target site 45 in a heterozygous form as expected. We also cloned the 15 main sgRNA45 off-target sites designated by ChIP-seq from the transgenic cattle genomes to evaluate the practical off-target effects. Finally, we obtained three indels in the WT Cas9-induced group (Additional file 3: Figure S8), but no typical indels occurred in any of the nine calves from the Cas9n group*.*

### *NRAMP1* insertion provides cattle with increased resistance to tuberculosis

**Fig. 7:**
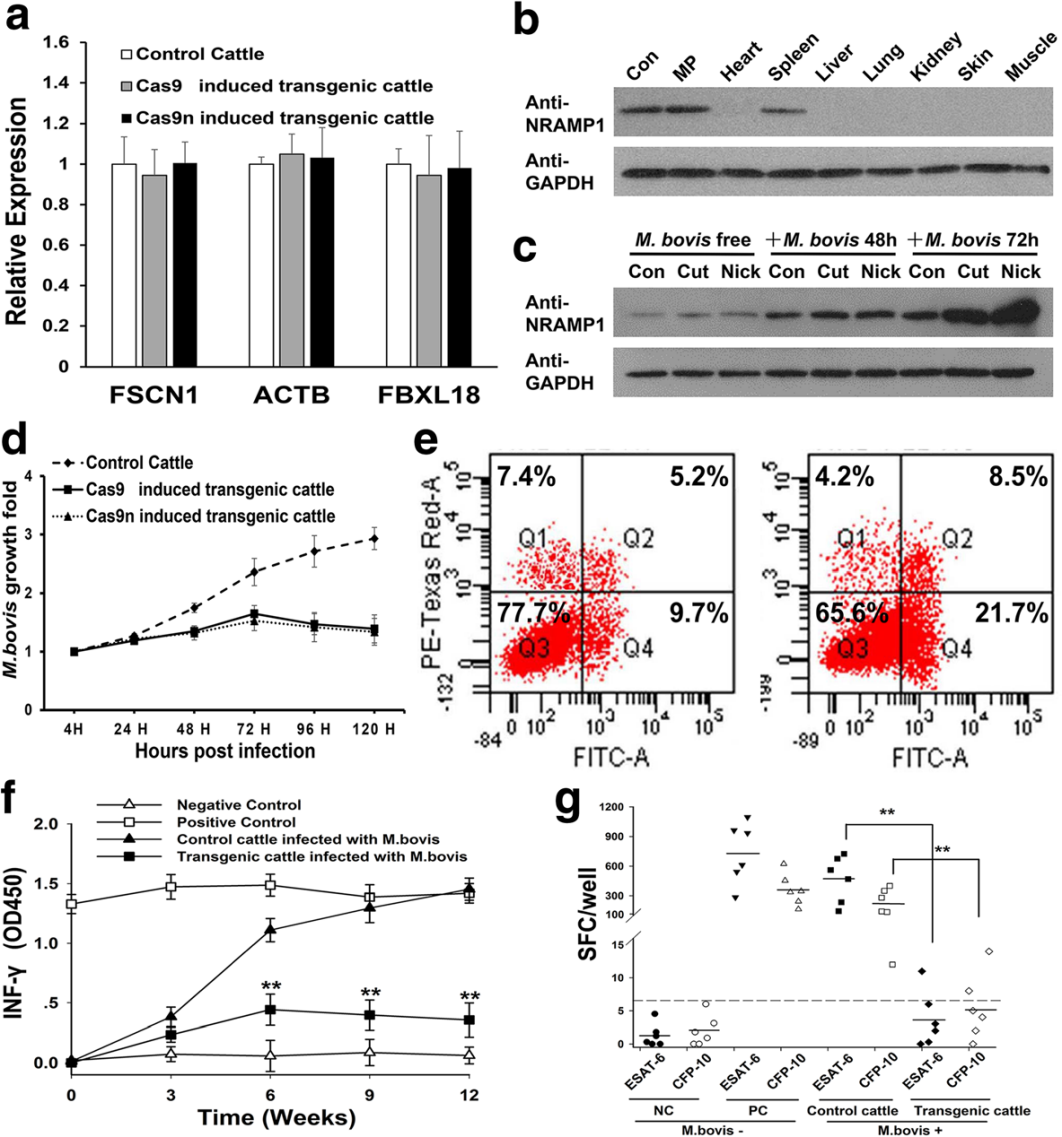
Assessment of the increased resistance of the transgenic cattle to tuberculosis. **a** The relative expression levels of the nearby endogenous genes in the F-A locus. Each sample was individually detected in macrophages through real-time PCR, but the data were analyzed according to the group. **b** The expression of *NRAMP1* was restricted to dedicated phagocytes. The organs were obtained from a pool of dead transgenic cattle. Con, *NRAMP1* over-expression Raw264.7 cells; *MP* macrophages. **c** The expression of *NRAMP1* was highly activated in the transgenic cattle following infection. All the samples were mixed monocyte-derived macrophages (MDMs) that were isolated from the blood of the same group of cattle as a pool. “Con” represents the control normal cattle. **d** Multiplication of *M. bovis* in MDMs from the control or transgenic cattle in vitro. The MDMs were separated from each animal individually and mixed according to group. *M. bovis* multiplication was determined via cfu assays. **e** Flow cytometry analysis of the cell death mechanism of the transgenic cattle MDMs after *M. bovis* infection. Necrotic (Q1), early apoptotic (Q2), and late apoptotic (Q4). *Left*, infected experiment control MDMs. *Right*, infected transgenic MDMs. **f** Amounts of IFN-γ produced in the experimental control (*n* = 6) and transgenic (*n* = 6) cattle at regular intervals of 12 weeks. **g** Concentrations of ESAT-6 and CFP-10 IFN-γ–producing SFCs among the PBMCs of the control and transgenic cattle

No significant difference was detected in the relative expression levels of the nearby endogenous genes between the transgenic and control cattle based on real-time PCR analysis (Fig. 7a and Additional file 3: Table S7). Furthermore, no *NRAMP1* protein was detected in the skin, muscle, heart, liver, lungs, or kidneys of the transgenic cattle by western blot (Fig. 7b). These results indicated that the expression of *NRAMP1* was still restricted to dedicated phagocytes as observed in conventional cattle.

To evaluate the biological responses of the transgenic cattle to *M. bovis* infection, we isolated peripheral blood mononuclear cells (PBMCs) from transgenic and normal Holstein–Friesian cows. Subsequently, the PBMCs were induced into macrophages via stimulation with granulocyte macrophage colony-stimulating factor (GM-CSF) for the subsequent experiments as described in Additional file 3: Supplemental Methods and Figure S9. These two groups exhibited similar constitutive expression levels of *NRAMP1* and obviously different responses to *M. bovis* following infection. First, we observed the robust expression of *NRAMP1* for the elimination of intracellular mycobacteria in the transgenic groups and the endogenous NRAMP1 protein level also increased after infection (Fig. 7c). Second, the growth rate of *M. bovis* in the monocyte-derived macrophages (MDMs) from the transgenic cattle was lower than that of the normal group (Fig. 7d). Finally, a notable bias towards the macrophage cell death pathway after infection was observed (Fig. 7e). Flow cytometry analysis revealed that 7.4% of the challenged macrophages were necrotized in the normal group, whereas the necrosis rate in the transgenic cattle macrophages was 4.2%. Moreover, a nearly twofold increase in the rate of apoptosis after infection was detected in the transgenic cattle compared with the control group (30.2% ± 0.63% versus 14.9% ± 0.58%, *P* = 0.0036).

An in vivo challenge experiment was also performed to further estimate the ability of the transgenic cattle to resist tuberculosis. Six randomly selected transgenic cattle and six experimental control normal cattle (breed-matched, sex-matched, and age-matched with the transgenic cattle) were infected via endobronchial instillation with 5 × 10^4^ cfu of *M. bovis* [45]. Blood samples were collected at regular intervals throughout the challenge period. Next, IFN-γ release assays (IGRAs) were conducted to assess the pathology [45, 46]. The control cattle developed IFN-γ responses within three weeks of stimulation with purified bovine tuberculin protein derivatives (PPD-Bs) and the response continued to steadily increase throughout the 12-week challenge period (Fig. 7f). In contrast, the IFN-γ responses against the PPD-Bs in the transgenic cattle were significantly lower than those of the control group after infection (nine weeks: transgenic 0.40 ± 0.13 versus control 1.29 ± 0.12, *P* = 0.000; 12 weeks: transgenic 0.36 ± 0.14 versus control 1.45 ± 0.09, *P* = 0.000). After the challenge period, we also performed an MTB-specific enzyme-linked immunospot (ELISPOT) assay [47] to confirm our results. As illustrated in Fig. 7g, the average number of spot-forming cells (SFC) was significantly lower in the transgenic cattle than the control cattle (early secretory antigenic target-6 (ESAT-6): control 467.8 ± 235.3 versus transgenic 3.72 ± 4.18, *P* = 0.005; culture filtrate protein-10 (CFP-10): control 219.7 ± 148.1 versus transgenic 5.5 ± 4.96, *P* = 0.016). Moreover, the number of SFCs in the transgenic cattle was not significantly different from that in the negative control cattle (ESAT-6: negative control 1.3 ± 1.72 versus transgenic 3.72 ± 4.18, *P* = 0.219; CFP-10: negative control 1.97 ± 2.30 versus transgenic 5.5 ± 4.96, *P* = 0.145). These results indicated that the transgenic cattle exhibited increased resistance to *M. bovis*.

## Discussion

Genome editing has developed rapidly since the seminal discovery of programmable artificial endonucleases such as ZFN, TALEN, and Cas9. These programmable nucleases can produce site-specific DNA DSBs and these DSBs can enhance the efficiency of HDR by at least two orders of magnitude and/or trigger NHEJ and subsequently lead to targeted mutagenesis [48]. ZFNs and TALENs both require the complex engineering of highly specific DNA-binding domains for proper targeting. In contrast, the CRISPR/Cas9 system only requires a small artificial sgRNA to direct the Cas9 endonuclease to virtually any location in the genome [14]. Due to high efficiency, CRISPR/Cas9 technology can readily simultaneously target several loci including both alleles of the same gene [7, 10, 49].

However, considering its variable and substantial off-target effects, specificity remains an issue in this system. To date, no definitive rules are available for predicting Cas9 specificity, but several mutants and corresponding strategies have been proposed to minimize off-target cleavage events. These strategies include the use of double nickase [19, 20], short sgRNAs [50], and a fusion protein containing a catalytically inactive dCas9 and the endonuclease Fok1 [8, 51]. Because each single indel at an unexpected off-target site may be disastrous, benefits can be derived from these strategies for precise gene therapy and transgenic animal production is possible.

To our knowledge, the current study demonstrates the application of single Cas9n in livestock for the first time. This technique aims to efficiently induce gene insertion at a selected bovine locus and produce transgenic cattle with reduced off-target effects. Moreover, we further demonstrated that the inserted *NRAMP1* was correctly expressed and provided cattle with increased resistance to infection with *M. bovis*, which is the mycobacterial pathogen that causes bovine tuberculosis.

Because they have significant influence over targeted specificity, the putative transgenic locus and the specific target site should be selected through meticulous deliberation. Evaluating the practical functions of CRISPR/Cas9 at the individual sites was a significant task due to the variable mutation rates at the 14 computationally predicted target sites (Fig. 1d). Thus, we performed systematically selective processes, which included cleavage efficiency detection, recombination efficiency detection, and binding specificity mapping, on the selected target site 45 to ensure its quality performance. Moreover, the current study confirmed that this target site can provide the internal transgene with stable and tissue-specific expression (Fig. 7b and c) without disrupting adjacent genes (Figs. 6a, b, and 7a). This finding indicated that the target site 45 of the F-A locus might be a safe harbor for site-specific gene insertion in the bovine genome.

The exact mechanism by which a SSB leads to fewer off-target sites and reduced toxicity targeting compared with a DSB is not yet well understood [52], but this phenomenon is a very active area of research. Generally, the majority of chromosomal single-stranded nicks are rapidly detected and removed by the SSB repair (SSBR) pathway throughout the entire mitotic cycle and no genomic modification occurs during this process [53, 54]. Therefore, single Cas9n-induced HDRs commonly exhibit low efficiencies in some cell types and it has been suggested that this phenomenon should be leveraged for specific gene targeting [5]. Based on the notably high indel rate (~47%) of the WT CRISPR/Cas9 at the selected target site 45, the utilization of a single Cas9n-induced SSB to stimulate HDR with subsequent gene insertion is technically feasible. Additionally, the considerably high efficiencies of the generation of transgenic BFFs (~18% positive colony rate) and cattle (9/11 new-born calves) with a single Cas9n group suggests that single Cas9n can be regarded as a valid alternative to the classical WT Cas9 method. Recent studies have focused on improving the specificity of the Cas9 nuclease through rational engineering of the Cas9 protein [55, 56]. Considering these tools under development, a single nickase at a selected target site should exhibit excellent performance.

According to our previous studies [43, 44], obtaining homozygous transgene knockin colonies through conducting gene targeting operation once was difficult in BFFs. Although none of the 57 positive colonies gained biallelic *NRAMP1* knockin at target site 45, indels were observed in the other allele in several clones of WT Cas9 group (Additional file 3: Table S6). This finding suggests that Cas9 can cleave both alleles. Hence, obtaining homozygous livestock for propagation is potential for future studies through increasing the amount of transgenic colonies or the use of small molecules and proteins that can increase HDR rates [57, 58].

Regarding the MDMs that were isolated from the transgenic cattle, more robust expression of *NRAMP1* was observed following the *M. bovis* challenge compared with the MDMs from the conventional cattle. However, no difference was detected in the constitutive expression levels of *NRAMP1* between these groups. The *NRAMP1* transgenic cattle exhibited increased resistance to *M. bovis*. This finding might be associated with the activity of the apoptotic pathway after infection, which can reduce the effectiveness of the intracellular survival mechanisms of *M. bovis* [59]*.* We speculate that the open genomic structure of the inserted *NRAMP1* locus might enhance interactions with transcriptional activators, but the specific mechanism by which *NRAMP1* determines the fate of macrophages should be further investigated.

## Conclusions

In summary, we demonstrated that a single Cas9n can be used for gene insertion at a selected target site in the cattle genome and that this method is advantageous in terms of avoiding additional indel mutations. The resulting transgenic cattle exhibited increased resistance to *M. bovis* infection. Our study provides an avenue to develop the CRISPR/Cas9 system for agriculture applications.

## Methods

### Construction of Cas9 plasmids

The query sequence of the F-A locus for the design tool ZiFiT is available in Additional file 1: Supplemental Dataset S1; the 80 potential target sites were inputted into the web-based off-targets predication tool Cas-OFFinder [29] to evaluate the mismatches number. Through the basic lookup and filter function, we performed statistical analysis of the mismatches number and location of all the 80 sgRNAs, individually. All the data during this procedure are shown in Additional file 2: Supplemental Dataset S2.

To generate the sgRNA expression construct, we cloned sgRNA into the pSpCas9 (BB) vector for co-expression with Cas9 [30]. Primer sequences for sgRNA cloning are shown in Additional file 3: Table S1. The restriction enzyme BbsI (NEB, UK) and ligation reaction buffer Solution I (Takara, Tokyo, Japan) were used for plasmid construction. The Cas9n with D10A HNH^+^/RuvC^–^ mutant plasmid pSpCas9n (BB)-2A-GFP (PX461, Addgene plasmid # 48140) was also received from Addgene. The catalytically inactive, double mutant dCas9 plasmids were generated by introducing a H840A mutation into the Cas9n plasmid using the QuikChange Site-Directed Mutagenesis kit (Stratagene, CA, USA).

### Surveyor nuclease assay

BFFs were transfected with the DNA constructs described above. Cells were incubated at 37 °C for 72 h post transfection prior to genomic DNA extraction. In brief, genomic DNA was extracted using a Universal Genomic DNA Extraction Kit (TaKaRa). The genomic region flanking the CRISPR target locus was PCR amplified using the following primers: Surveyor-detect-F (5′-GACTCCTGTAACCTCTGTCCCTG-3′) and Surveyor-detect-R (5′-TCAGCAGTTGCGGTTCG-3′). The products were purified by PCR Clean-Up Kit (Axygen, CA, USA), digested with Surveyor nuclease (Transgenomic, NE, USA) and analyzed by agarose gel electrophoresis according to the manufacturers’ instructions.

Genome modification rates can be estimated first by calculating the relative intensities of digestion products a, b, and the undigested band c. The frequency of cutting *f*_cut_ is then given by (a + b)/(a + b + c). The following formula, based on the binomial probability distribution of duplex formation, estimates the percentage of indels in the sample:

$$ \mathrm{Indel}\ \%=\left(1-\sqrt{\left(1\hbox{-} {f}_{cut}\right)}\right)\ast 100 $$

### Cell culture and transfection

BFFs were isolated from 35–40-day-old fetuses and maintained in DMEM/F12 (Gibco, NY, USA) supplemented with 10% FBS at 37 °C in a 5% CO_2_ environment. Raw 264.7 cells (ATCC, VA, USA) were cultured with 1640 (Gibco) supplemented with 10% FBS (Gibco). BFFs were harvested using 0.25% trypsin/EDTA solution (Gibco). Cells (1 × 10^7^) were resuspended in Opti-MEM (Gibco), mixed, if not otherwise indicated, with 12 μg of linearized donor plasmid and 10 μg of Cas9-encoding plasmid, and electroporated at 510 V with three pulses of 1-ms duration using the BTX Electro-cell manipulator ECM2001 (BTX Technologies, CA, USA). Electroporated cells were sorted via flow cytometry and plated on 10-cm plates at 1 × 10^6^ cells per plate. Individual colonies were selected and expanded after puromycin selection (2 μg/mL) 10–14 days after electroporation.

### ChIP experiments and data analysis

One million cells were seeded to 6-cm plates on day 1, transfected with reformed sgRNAs-plasmids (or dCas9 plasmids without sgRNA) on day 2, and transferred to 10-cm plates on day 3; and cross-linking was done on day 4 approximately about five million cells. Then the transfected cells were subjected to standard ChIP protocol in Pierce Agarose ChIP Kit (Thermo Scientific, NH, USA). The primers used to detect DNA fragments are shown below. For: 5′- TAAGGATTAGAAGGCAGAGTG -3′; Rev: 5′- CCTGTTTTCGTGGAGTTTA -3′. DNA fragments after ChIP were end-repaired, A-tailed, and ligated with indexed adapters. The products were purified and enriched by PCR to create the final cDNA libraries. Target bands were harvested by 2% agarose gel electrophoresis and quantified by Agilent 2200. The tagged cDNA libraries were pooled in equal ratios and used for 101-bp paired-end sequencing in a single lane of the Illumina HiSeq™ 2500 with 51 + 7 cycles.

Before read mapping, clean reads were obtained from the raw reads by removing the adaptor sequences, reads with >5% ambiguous bases (noted as N) and low-quality reads containing more than 20% of bases with qualities of <20. The clean reads were then aligned to *B. taurus* genome (version: Btau_4.6.1) using the BWA program (0.7.8-r455). For alignment, preliminary experiments were performed to optimize the alignment parameters [32]. We applied the Peak calling between sgRNA and control groups based on the mapped reads utilizing MACS. To eliminate the effect of the Cas9 protein, the peak calling between each sgRNA sample and Venn Analysis between each peak calling result were applied. To analyze sequences in the resulting significant regions that matched the sgRNA guiding sequence and PAM sequence (23 bp), the sgRNA selection tool Cas-Offinder and PATMAN alignment tool [60] were used to align the target sequences to those present under the peak regions. The relationship between the CpG island and binding site were performed based on the UCSC genome annotation.

### Detection of individual colonies by PCR

Drug-resistant cell clones derived from the transfected cell populations were collected by trypsinization and 70% of these were plated in serum-containing culture medium and expanded. The remaining clones were resuspended in 20 μL of PCR-compatible lysis buffer (10 mM of Tris-HCl, pH 8.5; 50 mM of KCl; 1.5 mM of MgCl_2_; 0.5% NP-40; 0.5% Tween-20; 400 g/mL of proteinase K) for PCR analysis. The lysates were incubated at 65 °C for 60 min and then at 95 °C for 15 min. To distinguish the *Nramp1*-targeted cell clones, 2 μL of the DNA lysate was added to a PCR reaction with PCR primers for 5′-junction PCR and subjected to PCR with EmeraldAmp (TaKaRa) using standard methods. Subsequently, 3′-junction PCR was performed on the positive clones to confirm the correct targeting events. The primers used for junction PCR were as follows: 5′-junction, Lj F (5′-AGTTGTGCCCTCCGTGTA-3′) and Lj R (5′-CTGCCATGCCCACTCAT-3′); 3′-junction, Rj F (5′-GCGCATGGCCGAGTTGA-3′) and Rj R (5′-CCCGCATTGCTCCCTCT-3′).

### Southern blot analysis

Probes for southern blot were amplified from cattle genomic DNA using the following primers: Probe 1, p1F (5′-CCAGTTCTTTGATGGGTGT-3′) and p1R (5′-GGCTTGACAGAAGGGTATG-3′); and Probe 2, p2F (5′-AGCAAGCAGGAGACGTGGAA-3′) and p2R (5′-CGCTCGTAGAAGGGGAGGTT-3′). PCR products were labeled with digoxigenin using the DIG-High Prime DNA Labeling and Detection Starter Kit II (Roche Diagnostics, IN, USA). Hind III-digested genomic DNA was separated on 1% (wt/vol) agarose gels, transferred to a nylon membrane (GE Healthcare, USA), and hybridized with 3′-end digoxigenin-labeled probes. The following procedures were performed using the DIG-High Prime DNA Labeling and Detection Starter Kit II according to the manufacturer’s instructions.

### Nuclear transfer

Ovaries were collected from the local abattoir and transported to the laboratory within 4–6 h in sterile saline at 37 °C. In vitro maturation of oocytes, enucleation, microinjection, and fusion of reconstructed oocytes were carried out in our laboratory according to previously described methods [44]. The reconstructed oocytes were cultured until they developed to the blastocyst stage. Three or four fresh day 7 blastocysts produced in vitro were non-surgically transferred to randomly assigned synchronized recipient heifers on day 7 after estrus. Pregnancy was diagnosed by rectal palpation on day 35 and confirmed by ultrasonography on day 60 after blastocyst transfer.

### Cfu assay

Infection with *M. bovis* was performed by the State Key Laboratory of Veterinary Etiological Biology (Lanzhou, China). In brief, a bacterial suspension corresponding to a multiplicity of infection (MOI) of 10:1 (∼10^7^ bacteria per 10^6^ cells) was added to the medium and incubated at 37 °C and 5% (vol/vol) CO_2_ for 4 h. Cells were then washed extensively with PBS to remove non-ingested bacteria. At the time points indicated in the text after infection, bacterial cfu were quantitated by plating on 7H10 agar plates (Difco Laboratories, Detroit, Mich) and incubated for eight weeks at 37 °C.

### *M. bovis* challenge experiments

We had set a control group and an experimental group in the subsequent experiments. Skin tests and IFN-γ assays were performed to confirm that cattle were infected with *M. bovis*. The control group comprised a negative control (a normal animal without the transgene or *M. bovis* infection) and a positive control (a normal animal without the transgene but infected with *M. bovis* by endobronchial instillation and diagnosed as tuberculous). The experimental groups comprised the control (a normal animal without the transgene or *M. bovis* infection; breed-matched, sex-matched, and age-matched with the transgenic cattle) and transgenic animals. Positive controls used for the challenge experiment were produced by endobronchial instillation with 5 × 10^4^ cfu of *M. bovis*. For the challenge experiments, all the calves of experimental groups were infected with 5 × 10^4^ cfu of *M. bovis* (strain AF 2212/97) by endobronchial instillation as previously described [45, 61]. Then blood samples were collected and IFN-γ assays were performed to monitor the level of IFN-γ release at the time points indicated in Fig. 7f. At the end of the experimental period, the calves were killed by i.v. injection of sodium phenobarbitone.

### IGRAs

IGRAs were performed using a BOVIGAM kit (Prionics AG, Schlieren, Switzerland) according to the manufacturer’s instructions. In brief, whole-blood samples were incubated with PPD-B to stimulate the lymphocytes to secrete IFN-γ. The plasma supernatants were harvested after 24 h of incubation and IFN-γ was estimated using a sandwich enzyme immunoassay. Optical density at 450 nm was determined using a VICTOR × 5 Multilabel Plate Reader (PerkinElmer, USA).

### ELISPOT assay

MTB-specific ELISPOT assays were performed after the challenge period as previously described [46, 47]. In brief, ELISPOT plates (Millipore, MA, USA) were coated overnight at 4 °C with mouse anti-bovine IFN-γ monoclonal antibody (Thermo Scientific). Peripheral blood mononuclear cells (PBMCs) (2 × 10^5^) then were added and cultured at 37 °C for 24 h. The cells were stimulated with ESAT-6 and CFP-10 peptides in separate wells following procedures performed strictly according to the manufacturer’s recommendations. The response of stimulated cultures was considered positive when the test well contained at least six more spots than the control well.

## Additional files

Additional file 1: Supplemental dataset S1. (PDF 117 kb)
Additional file 2: Supplemental dataset S2. (XLSX 8654 kb)
Additional file 3: Supplemental Methods, Figures, and Tables. Supplemental Methods, Figures S1–S9, and Tables S1–S7. (PDF 1638 kb)
