# Supplementary material for: Single Cas9 nickase induced generation of NRAMP1 knockin cattle with reduced off-target effects
Source: Genome Biol. 2017 Feb 1;18:13. doi: 10.1186/s13059-016-1144-4 (PMC5286826; doi:10.1186/s13059-016-1144-4)
Supplement: Additional file 1: — Supplemental dataset S1. (PDF 117 kb) [file 13059_2016_1144_MOESM1_ESM.pdf]

Supplemental Dataset S1

```
WT-cow        AGCTAATCTC CAGGCCTAGG AAGCACTTTC CACCTCCTGG CTGAATTCCA
Trans-cow     AGCTAATCTC CAGGCCTAGG AAGCACTTTC CACCTCCTGG CTGAATTCCA
pDonor-1      ---------- ---------- ---------- ---------- ----------
pDonor-2      ---------- ---------- ---------- ---------- ----------

WT-cow        AGGAGACATT CTTGTTCTGT GACCCTGCAG CTTGTTTCCT TCTCTGGGCC
Trans-cow     AGGAGACATT CTTGTTCTGT GACCCTGCAG CTTGTTTCCT TCTCTGGGCC
pDonor-1      ---------- ---------- ---------- ---------- ----------
pDonor-2      ---------- ---------- ---------- ---------- ----------

WT-cow        AAGGTGGAAG CTGACGAGGA GAGAAAAGGG GCGCCTGGCC ACGTAGGGGG
Trans-cow     AAGGTGGAAG CTGACGAGGA GAGAAAAGGG GCGCCTGGCC ACGTAGGGGG
pDonor-1      ---------- ---------- ---------- ---------- ----------
pDonor-2      ---------- ---------- ---------- ---------- ----------

WT-cow        AGGGAAGCCC AGAGGCTCTG CAGCTGCCGC CTGCTGCAGA GTTCTTGTCC
Trans-cow     AGGGAAGCCC AGAGGCTCTG CAGCTGCCGC CTGCTGCAGA GTTCTTGTCC
pDonor-1      ---------- ---------- ---------- ---------- ----------
pDonor-2      ---------- ---------- ---------- ---------- ----------
```

Probe 1-primer-1F

```
WT-cow        CTGGTCACCA ACCAGTTCTT TGATGGGTGT CATGGAACCC CCAGCGCTGA
Trans-cow     CTGGTCACCA ACCAGTTCTT TGATGGGTGT CATGGAACCC CCAGCGCTGA
pDonor-1      ---------- ---------- ---------- ---------- ----------
pDonor-2      ---------- ---------- ---------- ---------- ----------

WT-cow        GCACCAGAGG CTGGCCTGGT AAGCTTGTGT CCAACTCCCA AGAGGGCCTG
Trans-cow     GCACCAGAGG CTGGCCTGGT AAGCTTGTGT CCAACTCCCA AGAGGGCCTG
pDonor-1      ---------- ---------- ---------- ---------- ----------
pDonor-2      ---------- ---------- ---------- ---------- ----------

WT-cow        TCTCTTGCTC TTATGGGCTC TAGAACCTAA GTTTAACCCA GCTCTGTGAG
Trans-cow     TCTCTTGCTC TTATGGGCTC TAGAACCTAA GTTTAACCCA GCTCTGTGAG
pDonor-1      ---------- ---------- ---------- ---------- ----------
pDonor-2      ---------- ---------- ---------- ---------- ----------

WT-cow        CCTCGCTCAG ACCCTGCTCA CCTCAATCTC CCCACCTATA CACATGGACC
Trans-cow     CCTCGCTCAG ACCCTGCTCA CCTCAATCTC CCCACCTATA CACATGGACC
pDonor-1      ---------- ---------- ---------- ---------- ----------
pDonor-2      ---------- ---------- ---------- ---------- ----------

WT-cow        TGAATTCAAG GCCCAGCACA TTCCTCCCAA GGGGTAAGGG CACACAGTCC
Trans-cow     TGAATTCAAG GCCCAGCACA TTCCTCCCAA GGGGTAAGGG CACACAGTCC
pDonor-1      ---------- ---------- ---------- ---------- ----------
pDonor-2      ---------- ---------- ---------- ---------- ----------
```

```
WT-cow       CCTCTGAGCT GTGCTAAGAG GGCTCCCCCA AGCCCACGCC CATCCCAACT
Trans-cow    CCTCTGAGCT GTGCTAAGAG GGCTCCCCCA AGCCCACGCC CATCCCAACT
pDonor-1     ---------- ---------- ---------- ---------- ----------
pDonor-2     ---------- ---------- ---------- ---------- ----------

                                          5' Junction primer-F
WT-cow       CTGCTTGCCC AGTTGGGGTT TTGCCCAGTT GTGCCCTCCG TGTACTCTGC
Trans-cow    CTGCTTGCCC AGTTGGGGTT TTGCCCAGTT GTGCCCTCCG TGTACTCTGC
pDonor-1     ---------- ---------- ---------- ---------- ----------
pDonor-2     ---------- ---------- ---------- ---------- ----------

WT-cow       TGAGAGCTGA CCAGAGCAGA AATGCTATTG AGCCCAGACC TCCGTACTGA
Trans-cow    TGAGAGCTGA CCAGAGCAGA AATGCTATTG AGCCCAGACC TCCGTACTGA
pDonor-1     ---------- ---------- ---------- ---------- ----------
pDonor-2     ---------- ---------- ---------- ---------- ----------

WT-cow       CGTTTCTATG GACTCCAAAG GGCCCAGCCT CATCAAATGT TTGCAGTTCT
Trans-cow    CGTTTCTATG GACTCCAAAG GGCCCAGCCT CATCAAATGT TTGCAGTTCT
pDonor-1     ---------- ---------- ---------- ---------- ----------
pDonor-2     ---------- ---------- ---------- ---------- ----------

                     Probe 1-primer-1R
WT-cow       CCCAACATAC CCTTCTGTCA AGCCCTCTCT AAATTGGCTC CTTTGTGAAC
Trans-cow    CCCAACATAC CCTTCTGTCA AGCCCTCTCT AAATTGGCTC CTTTGTGAAC
pDonor-1     ---------- ---------- ---------- ---------- ----------
pDonor-2     ---------- ---------- ---------- ---------- ----------

WT-cow       TCCTATTCAC ATGTCAAAGC CCAGCCCCCA AAACCCCATC CTATAGGAAG
Trans-cow    TCCTATTCAC ATGTCAAAGC CCAGCCCCCA AAACCCCATC CTATAGGAAG
pDonor-1     ---------- ---------- ---------- ---------- ----------
pDonor-2     ---------- ---------- ---------- ---------- ----------

WT-cow       CCTTGCTTGA TCCTCGGACT GGGTTGCCCC TCCTGCAGTT CAGCACCTCA
Trans-cow    CCTTGCTTGA TCCTCGGACT GGGTTGCCCC TCCTGCAGTT CAGCACCTCA
pDonor-1     ---------- ---------- ---------- ---------- ----------
pDonor-2     ---------- ---------- ---------- ---------- ----------

WT-cow       TGTGTTCAGG ATATCAGACA GTTGTTTTCC CCAGGGACCC CTGGAGAACA
Trans-cow    TGTGTTCAGG ATATCAGACA GTTGTTTTCC CCAGGGACCC CTGGAGAACA
pDonor-1     ---------- ---------- ---------- ---------- ----------
pDonor-2     ---------- ---------- ---------- ---------- ----------

               Not I                     Left homology arm (777bp)
WT-cow       GGGTTCAAGT CTGACCCGTG AGTGTTATCA GACACCAGGT GAGTCAGCTG
Trans-cow    GGGTTCAAGT CTGACCCGTG AGTGTTATCA GACACCAGGT GAGTCAGCTG
pDonor-1     GCGGCCGCGT CTGACCCGTG AGTGTTATCA GACACCAGGT GAGTCAGCTG
pDonor-2     GCGGCCGCGT CTGACCCGTG AGTGTTATCA GACACCAGGT GAGTCAGCTG
```

```
WT-cow        CAGTCCAGAG ACCCGAAGAC TTGCTGTGAC ACCTTCCCTC CTATACTCCC
Trans-cow     CAGTCCAGAG ACCCGAAGAC TTGCTGTGAC ACCTTCCCTC CTATACTCCC
pDonor-1      CAGTCCAGAG ACCCGAAGAC TTGCTGTGAC ACCTTCCCTC CTATACTCCC
pDonor-2      CAGTCCAGAG ACCCGAAGAC TTGCTGTGAC ACCTTCCCTC CTATACTCCC

WT-cow        CGTCAACATC CGCTGGAAGG TGTGAGCCCT GAGCTGGCCC AACCCCTTCC
Trans-cow     CGTCAACATC CGCTGGAAGG TGTGAGCCCT GAGCTGGCCC AACCCCTTCC
pDonor-1      CGTCAACATC CGCTGGAAGG TGTGAGCCCT GAGCTGGCCC AACCCCTTCC
pDonor-2      CGTCAACATC CGCTGGAAGG TGTGAGCCCT GAGCTGGCCC AACCCCTTCC

WT-cow        TCCCCAGGCC TATTCCTCAT CAGCTTTGGC AGTCCTAGGA CATTATCCCT
Trans-cow     TCCCCAGGCC TATTCCTCAT CAGCTTTGGC AGTCCTAGGA CATTATCCCT
pDonor-1      TCCCCAGGCC TATTCCTCAT CAGCTTTGGC AGTCCTAGGA CATTATCCCT
pDonor-2      TCCCCAGGCC TATTCCTCAT CAGCTTTGGC AGTCCTAGGA CATTATCCCT
```

Left homology arm (777bp)

```
WT-cow        GCCAGGGCTG TGCCCACACC CTCCTGGGAC AGGGTGCTCA CACCTTGGAG
Trans-cow     GCCAGGGCTG TGCCCACACC CTCCTGGGAC AGGGTGCTCA CACCTTGGAG
pDonor-1      GCCAGGGCTG TGCCCACACC CTCCTGGGAC AGGGTGCTCA CACCTTGGAG
pDonor-2      GCCAGGGCTG TGCCCACACC CTCCTGGGAC AGGGTGCTCA CACCTTGGAG
```

Surveyor-detect-primer –F →

```
WT-cow        AGACAGCACA TCGACTCCTG TAACCTCTGT CCCTGCTGTA GAGAACTGGA
Trans-cow     AGACAGCACA TCGACTCCTG TAACCTCTGT CCCTGCTGTA GAGAACTGGA
pDonor-1      AGACAGCACA TCGACTCCTG TAACCTCTGT CCCTGCTGTA GAGAACTGGA
pDonor-2      AGACAGCACA TCGACTCCTG TAACCTCTGT CCCTGCTGTA GAGAACTGGA

WT-cow        TCCATTCATC TTCCCCTCCC TTCCTCTCCT CCTTCCTTCC TTCCTTCTAT
Trans-cow     TCCATTCATC TTCCCCTCCC TTCCTCTCCT CCTTCCTTCC TTCCTTCTAT
pDonor-1      TCCATTCATC TTCCCCTCCC TTCCTCTCCT CCTTCCTTCC TTCCTTCTAT
pDonor-2      TCCATTCATC TTCCCCTCCC TTCCTCTCCT CCTTCCTTCC TTCCTTCTAT

WT-cow        CTGAAGACAG AAGCTCAAAA CTGCACTTAC TTACATCCTG AGAGGGGGAC
Trans-cow     CTGAAGACAG AAGCTCAAAA CTGCACTTAC TTACATCCTG AGAGGGGGAC
pDonor-1      CTGAAGACAG AAGCTCAAAA CTGCACTTAC TTACATCCTG AGAGGGGGAC
pDonor-2      CTGAAGACAG AAGCTCAAAA CTGCACTTAC TTACATCCTG AGAGGGGGAC

WT-cow        CCCACAGTGA TCAAAACAGA CATGGTCCCT GCCAGTGGAG CATGGAGGAA
Trans-cow     CCCACAGTGA TCAAAACAGA CATGGTCCCT GCCAGTGGAG CATGGAGGAA
pDonor-1      CCCACAGTGA TCAAAACAGA CATGGTCCCT GCCAGTGGAG CATGGAGGAA
pDonor-2      CCCACAGTGA TCAAAACAGA CATGGTCCCT GCCAGTGGAG CATGGAGGAA

WT-cow        GAGAGCATGA TCACACACTT ACATCCAATA ACTGGCTGTG ATGAGCATGA
Trans-cow     GAGAGCATGA TCACACACTT ACATCCAATA ACTGGCTGTG ATGAGCATGA
pDonor-1      GAGAGCATGA TCACACACTT ACATCCAATA ACTGGCTGTG ATGAGCATGA
pDonor-2      GAGAGCATGA TCACACACTT ACATCCAATA ACTGGCTGTG ATGAGCATGA
```

```
WT-cow       GGAAGAAGTG TAGGAGCCAC AAGAACCTTG CCCTTGTCCA GGAATCCAGG
Trans-cow    GGAAGAAGTG TAGGAGCCAC AAGAACCTTG CCCTTGTCCA GGAATCCAGG
pDonor-1     GGAAGAAGTG TAGGAGCCAC AAGAACCTTG CCCTTGTCCA GGAATCCAGG
pDonor-2     GGAAGAAGTG TAGGAGCCAC AAGAACCTTG CCCTTGTCCA GGAATCCAGG

WT-cow       GGGGCTTCCT GGAAGAGGTG ATATGTACAC CAAACCCTAA AGGAGAAGCA
Trans-cow    GGGGCTTCCT GGAAGAGGTG ATATGTACAC CAAACCCTAA AGGAGAAGCA
pDonor-1     GGGGCTTCCT GGAAGAGGTG ATATGTACAC CAAACCCTAA AGGAGAAGCA
pDonor-2     GGGGCTTCCT GGAAGAGGTG ATATGTACAC CAAACCCTAA AGGAGAAGCA

WT-cow       GAACTTAAAG AAGCAGAAAG GATGCGTGTG GGGAGCAGAG TCCATTACAG
Trans-cow    GAACTTAAAG AAGCAGAAAG GATGCGTGTG GGGAGCAGAG TCCATTACAG
pDonor-1     GAACTTAAAG AAGCAGAAAG GATGCGTGTG GGGAGCAGAG TCCATTACAG
pDonor-2     GAACTTAAAG AAGCAGAAAG GATGCGTGTG GGGAGCAGAG TCCATTACAG

WT-cow       ACCGAGAGAA CAGGAAGGGC AGAGGCCCTG AGGCAGATTT GGTGTTCTAA
Trans-cow    ACCGAGAGAA CAGGAAGGGC AGAGGCCCTG AGGCAGATTT GGTGTTCTAA
pDonor-1     ACCGAGAGAA CAGGAAGGGC AGAGGCCCTG AGGCAGATTT GGTGTTCTAA
pDonor-2     ACCGAGAGAA CAGGAAGGGC AGAGGCCCTG AGGCAGATTT GGTGTTCTAA
```

Left homology arm (777bp)

```
WT-cow       GGATTAGAAG GCAGAGTGTC TGGGAACACA GAGTAAGGCT GGAGGGGCAA
Trans-cow    GGATTAGAAG GCAGAGTGTC TGGGAACACA GAGTAAGGCT GGAGGGGCAA
pDonor-1     GGATTAGAAG GCAGAGTGTC TGGGAACACA GAGTAAGGCT GGAGGGGCAA
pDonor-2     GGATTAGAAG GCAGAGTGTC TGGGAACACA GAGTAAGGCT GGAGGGGCAA
```

Sal I

```
WT-cow       GGGGGTGAGG TTAGCCACAC TCCCAAACTA GCCGCGGTCT CCACAAC---
Trans-cow    GGGGGTGAGG TTAGCCACAC TCCCAAACTA GCCGCGTCGA CCATCACAGC
pDonor-1     GGGGGTGAGG TTAGCCACAC TCCCAAACTA GCCGCGTCGA CCATCACAGC
pDonor-2     GGGGGTGAGG TTAGCCACAC TCCCAAACTA GCCGCGTCGA CCATCACAGC

WT-cow       ---------- ---------- ---------- ---------- ----------
Trans-cow    CTCCTACCCC AGCCCTATCC CCCAGCCACC CCACGTGAGT CCAAAGTAGT
pDonor-1     CTCCTACCCC AGCCCTATCC CCCAGCCACC CCACGTGAGT CCAAAGTAGT
pDonor-2     CTCCTACCCC AGCCCTATCC CCCAGCCACC CCACGTGAGT CCAAAGTAGT
```

***NRAMP1* promoter (1742bp)**

```
WT-cow       ---------- ---------- ---------- ---------- ----------
Trans-cow    GATTGCAAAC CTGTGTAAAC CCTAGAGGTC AAGGCTGGTG ACTTGAGCAA
pDonor-1     GATTGCAAAC CTGTGTAAAC CCTAGAGGTC AAGGCTGGTG ACTTGAGCAA
pDonor-2     GATTGCAAAC CTGTGTAAAC CCTAGAGGTC AAGGCTGGTG ACTTGAGCAA

WT-cow       ---------- ---------- ---------- ---------- ----------
Trans-cow    CGGGATTGCA CATGGACAAA CCACAGTGCT GCTCTGTCAT TCGTGACTCA
pDonor-1     CGGGATTGCA CATGGACAAA CCACAGTGCT GCTCTGTCAT TCGTGACTCA
pDonor-2     CGGGATTGCA CATGGACAAA CCACAGTGCT GCTCTGTCAT TCGTGACTCA
```

```
WT-cow       ---------- ---------- ---------- ---------- ----------
Trans-cow    ACCATTCACC CCTCTACCCC TGCCTCTCCC GTCCCTCCAG CCTCTCTTTT
pDonor-1     ACCATTCACC CCTCTACCCC TGCCTCTCCC GTCCCTCCAG CCTCTCTTTT
pDonor-2     ACCATTCACC CCTCTACCCC TGCCTCTCCC GTCCCTCCAG CCTCTCTTTT
```

```
WT-cow       ---------- ---------- ---------- ---------- ----------
Trans-cow    CAACGAGGAG CCAGGAAATT GGCTGCCTCC CCCCATCCTG GGGTTCCAAG
pDonor-1     CAACGAGGAG CCAGGAAATT GGCTGCCTCC CCCCATCCTG GGGTTCCAAG
pDonor-2     CAACGAGGAG CCAGGAAATT GGCTGCCTCC CCCCATCCTG GGGTTCCAAG
```

```
WT-cow       ---------- ---------- ---------- ---------- ----------
Trans-cow    CTTTGGCTCA AGTTTTTGAC TCAACCTGAG GAAACATCAC ATCCCAGATC
pDonor-1     CTTTGGCTCA AGTTTTTGAC TCAACCTGAG GAAACATCAC ATCCCAGATC
pDonor-2     CTTTGGCTCA AGTTTTTGAC TCAACCTGAG GAAACATCAC ATCCCAGATC
```

```
WT-cow       ---------- ---------- ---------- ---------- ----------
Trans-cow    AAGCCTGGGC TTGGGAACAA GCCCTGATTT TCTCTGTGTC TGGAGGTCCA
pDonor-1     AAGCCTGGGC TTGGGAACAA GCCCTGATTT TCTCTGTGTC TGGAGGTCCA
pDonor-2     AAGCCTGGGC TTGGGAACAA GCCCTGATTT TCTCTGTGTC TGGAGGTCCA
```

***NRAMP1* promoter (1742bp)**

```
WT-cow       ---------- ---------- ---------- ---------- ----------
Trans-cow    GACTTTGGAG TTGGAAACAC CATGACAATT CGAGTTTTAA ATCTTCTTGG
pDonor-1     GACTTTGGAG TTGGAAACAC CATGACAATT CGAGTTTTAA ATCTTCTTGG
pDonor-2     GACTTTGGAG TTGGAAACAC CATGACAATT CGAGTTTTAA ATCTTCTTGG
```

5' Junction primer-R ←

```
WT-cow       ---------- ---------- ---------- ---------- ----------
Trans-cow    CAGAAAAGAA AGGATACAGG ATAGAGGACA TGAGTGGGCA TGGCAGGGAG
pDonor-1     CAGAAAAGAA AGGATACAGG ATAGAGGACA TGAGTGGGCA TGGCAGGGAG
pDonor-2     CAGAAAAGAA AGGATACAGG ATAGAGGACA TGAGTGGGCA TGGCAGGGAG
```

```
WT-cow       ---------- ---------- ---------- ---------- ----------
Trans-cow    AAGACTCTGA GGGAGGTAAG TGGGGGGATA AAGGAGTTGG CAGCTGGCAA
pDonor-1     AAGACTCTGA GGGAGGTAAG TGGGGGGATA AAGGAGTTGG CAGCTGGCAA
pDonor-2     AAGACTCTGA GGGAGGTAAG TGGGGGGATA AAGGAGTTGG CAGCTGGCAA
```

```
WT-cow       ---------- ---------- ---------- ---------- ----------
Trans-cow    GGAGAAGAGA TTGGGAGCTG GAAGGGTCTA GAGCTAACAG ATTGATCATC
pDonor-1     GGAGAAGAGA TTGGGAGCTG GAAGGGTCTA GAGCTAACAG ATTGATCATC
pDonor-2     GGAGAAGAGA TTGGGAGCTG GAAGGGTCTA GAGCTAACAG ATTGATCATC
```

```
WT-cow       ---------- ---------- ---------- ---------- ----------
Trans-cow    CTAAAGTGGA ATCTGGCCCA TAGAAACGCT TGCTTAGGTC ACCCAATATT
pDonor-1     CTAAAGTGGA ATCTGGCCCA TAGAAACGCT TGCTTAGGTC ACCCAATATT
pDonor-2     CTAAAGTGGA ATCTGGCCCA TAGAAACGCT TGCTTAGGTC ACCCAATATT
```

```
WT-cow        ---------- ---------- ---------- ---------- ----------
Trans-cow     TGCAAAACAT TTGAGCCCAG TATTCAAACA GCATATGTTG CATAAATTTT
pDonor-1      TGCAAAACAT TTGAGCCCAG TATTCAAACA GCATATGTTG CATAAATTTT
pDonor-2      TGCAAAACAT TTGAGCCCAG TATTCAAACA GCATATGTTG CATAAATTTT

WT-cow        ---------- ---------- ---------- ---------- ----------
Trans-cow     CCAGCATCCT TGACAAAACA GACGACTTAG TGACAGTGGA CCCACATCCT
pDonor-1      CCAGCATCCT TGACAAAACA GACGACTTAG TGACAGTGGA CCCACATCCT
pDonor-2      CCAGCATCCT TGACAAAACA GACGACTTAG TGACAGTGGA CCCACATCCT

WT-cow        ---------- ---------- ---------- ---------- ----------
Trans-cow     CACATGACAA CTGGCTGGAA CTCTGTGAGC ACTGCCCTCT TCAGACAGAG
pDonor-1      CACATGACAA CTGGCTGGAA CTCTGTGAGC ACTGCCCTCT TCAGACAGAG
pDonor-2      CACATGACAA CTGGCTGGAA CTCTGTGAGC ACTGCCCTCT TCAGACAGAG

WT-cow        ---------- ---------- ---------- ---------- ----------
Trans-cow     TTTATGACCT TTGTCTCCAT CATTCCCTCC CTCCCTCCCT CATAGCTCCA
pDonor-1      TTTATGACCT TTGTCTCCAT CATTCCCTCC CTCCCTCCCT CATAGCTCCA
pDonor-2      TTTATGACCT TTGTCTCCAT CATTCCCTCC CTCCCTCCCT CATAGCTCCA
```

NRAMP1 **promoter (1742bp)**

```
WT-cow        ---------- ---------- ---------- ---------- ----------
Trans-cow     TCGGTAAAGA ATCTGCCTGC AATGCAGGAG ACCTGGGTTC GATTTCTGGG
pDonor-1      TCGGTAAAGA ATCTGCCTGC AATGCAGGAG ACCTGGGTTC GATTTCTGGG
pDonor-2      TCGGTAAAGA ATCTGCCTGC AATGCAGGAG ACCTGGGTTC GATTTCTGGG

WT-cow        ---------- ---------- ---------- ---------- ----------
Trans-cow     TTGGGAAGAT CCCCTGGAGA AGGAAATGGC AACCCACTCC AGTATTCTTG
pDonor-1      TTGGGAAGAT CCCCTGGAGA AGGAAATGGC AACCCACTCC AGTATTCTTG
pDonor-2      TTGGGAAGAT CCCCTGGAGA AGGAAATGGC AACCCACTCC AGTATTCTTG

WT-cow        ---------- ---------- ---------- ---------- ----------
Trans-cow     CCTGGAAAAT CCCATGGACA GAGGAGCCTG GCGGGCTACG TCCATGAGGT
pDonor-1      CCTGGAAAAT CCCATGGACA GAGGAGCCTG GCGGGCTACG TCCATGAGGT
pDonor-2      CCTGGAAAAT CCCATGGACA GAGGAGCCTG GCGGGCTACG TCCATGAGGT

WT-cow        ---------- ---------- ---------- ---------- ----------
Trans-cow     CGCAAGTGTT GGACACGACT TAGTGACTAA ACCACCACCA CTCCCTATTA
pDonor-1      CGCAAGTGTT GGACACGACT TAGTGACTAA ACCACCACCA CTCCCTATTA
pDonor-2      CGCAAGTGTT GGACACGACT TAGTGACTAA ACCACCACCA CTCCCTATTA

WT-cow        ---------- ---------- ---------- ---------- ----------
Trans-cow     CTTCCCCCTG ATACTGAGGC TGAGCATCGT TTGTCATTGA GCACTGAGCC
pDonor-1      CTTCCCCCTG ATACTGAGGC TGAGCATCGT TTGTCATTGA GCACTGAGCC
pDonor-2      CTTCCCCCTG ATACTGAGGC TGAGCATCGT TTGTCATTGA GCACTGAGCC
```

```
WT-cow        ---------- ---------- ---------- ---------- ----------
Trans-cow     AGCACTGTTC TTCTCATTCC CAACCTGTCT CATAGTTTAT CTTACCTGCC
pDonor-1      AGCACTGTTC TTCTCATTCC CAACCTGTCT CATAGTTTAT CTTACCTGCC
pDonor-2      AGCACTGTTC TTCTCATTCC CAACCTGTCT CATAGTTTAT CTTACCTGCC

WT-cow        ---------- ---------- ---------- ---------- ----------
Trans-cow     TGGCCTCCTT GGGCACCTTT TGACCTTTTA GCTAGACTGA ACCCTTCTTA
pDonor-1      TGGCCTCCTT GGGCACCTTT TGACCTTTTA GCTAGACTGA ACCCTTCTTA
pDonor-2      TGGCCTCCTT GGGCACCTTT TGACCTTTTA GCTAGACTGA ACCCTTCTTA

WT-cow        ---------- ---------- ---------- ---------- ----------
Trans-cow     ACAGAAGGAA CAAGGTCAGA GACAAGACGT GATCTGGTGA CAATGTCAAG
pDonor-1      ACAGAAGGAA CAAGGTCAGA GACAAGACGT GATCTGGTGA CAATGTCAAG
pDonor-2      ACAGAAGGAA CAAGGTCAGA GACAAGACGT GATCTGGTGA CAATGTCAAG

WT-cow        ---------- ---------- ---------- ---------- ----------
Trans-cow     TGGATCGGTG GCAGAACCAA GACCAGAATT CCAGTGCCTC TTTCTTCTGG
pDonor-1      TGGATCGGTG GCAGAACCAA GACCAGAATT CCAGTGCCTC TTTCTTCTGG
pDonor-2      TGGATCGGTG GCAGAACCAA GACCAGAATT CCAGTGCCTC TTTCTTCTGG
```

***NRAMP1* promoter (1742bp)**

```
WT-cow        ---------- ---------- ---------- ---------- ----------
Trans-cow     GGATCTCTTT GGCCTGAATC TTCAGTGTAT GTCCCCGTGG CAGTAGCCGG
pDonor-1      GGATCTCTTT GGCCTGAATC TTCAGTGTAT GTCCCCGTGG CAGTAGCCGG
pDonor-2      GGATCTCTTT GGCCTGAATC TTCAGTGTAT GTCCCCGTGG CAGTAGCCGG

WT-cow        ---------- ---------- ---------- ---------- ----------
Trans-cow     AAGATCTTTC CCTCTAAGGC ACTCAGTGGG ACACTGAGAC CTAGACAGCC
pDonor-1      AAGATCTTTC CCTCTAAGGC ACTCAGTGGG ACACTGAGAC CTAGACAGCC
pDonor-2      AAGATCTTTC CCTCTAAGGC ACTCAGTGGG ACACTGAGAC CTAGACAGCC

WT-cow        ---------- ---------- ---------- ---------- ----------
Trans-cow     ATCAGAACTC CCTTCCTCCC CGTGGCCATG GGACCCAAGA TTAAAAAGGA
pDonor-1      ATCAGAACTC CCTTCCTCCC CGTGGCCATG GGACCCAAGA TTAAAAAGGA
pDonor-2      ATCAGAACTC CCTTCCTCCC CGTGGCCATG GGACCCAAGA TTAAAAAGGA

WT-cow        ---------- ---------- ---------- ---------- ----------
Trans-cow     GAAGGACCTG ACTGCTTGTA CAGGGTGAGG GTGGGCTAGC GGGCAGTCAC
pDonor-1      GAAGGACCTG ACTGCTTGTA CAGGGTGAGG GTGGGCTAGC GGGCAGTCAC
pDonor-2      GAAGGACCTG ACTGCTTGTA CAGGGTGAGG GTGGGCTAGC GGGCAGTCAC

WT-cow        ---------- ---------- ---------- ---------- ----------
Trans-cow     GTGATCTAGA CATGCATGCC CAAGTGGCAG GAAGTTGTCC AAAATCAGAG
pDonor-1      GTGATCTAGA CATGCATGCC CAAGTGGCAG GAAGTTGTCC AAAATCAGAG
pDonor-2      GTGATCTAGA CATGCATGCC CAAGTGGCAG GAAGTTGTCC AAAATCAGAG
```

```
WT-cow      ---------- ---------- ---------- ---------- ----------
Trans-cow   CTAACTTGGG AGAGGTGCAG AACTCAGAGT GCCTGGAAGG GAGCCAGAGG
pDonor-1    CTAACTTGGG AGAGGTGCAG AACTCAGAGT GCCTGGAAGG GAGCCAGAGG
pDonor-2    CTAACTTGGG AGAGGTGCAG AACTCAGAGT GCCTGGAAGG GAGCCAGAGG

WT-cow      ---------- ---------- ---------- ---------- ----------
Trans-cow   TGTTGTGGGG CTCAGGCCAG GAGGGGAACA AATGTCCTTG CTGTGGCCAC
pDonor-1    TGTTGTGGGG CTCAGGCCAG GAGGGGAACA AATGTCCTTG CTGTGGCCAC
pDonor-2    TGTTGTGGGG CTCAGGCCAG GAGGGGAACA AATGTCCTTG CTGTGGCCAC
```

NRAMP1 **promoter (1742bp)**

```
WT-cow      ---------- ---------- ---------- ---------- ----------
Trans-cow   CCAGACCCTT CCTCCAGAAC TGGCCACTTC TGCCTTTGGA AAGTGTTTCA
pDonor-1    CCAGACCCTT CCTCCAGAAC TGGCCACTTC TGCCTTTGGA AAGTGTTTCA
pDonor-2    CCAGACCCTT CCTCCAGAAC TGGCCACTTC TGCCTTTGGA AAGTGTTTCA

WT-cow      ---------- ---------- ---------- ---------- ----------
Trans-cow   CAACGTGCCT GCGTGTGAGG ACAGCTGAGG ATGGCTAAGA GGGCGTGGAC
pDonor-1    CAACGTGCCT GCGTGTGAGG ACAGCTGAGG ATGGCTAAGA GGGCGTGGAC
pDonor-2    CAACGTGCCT GCGTGTGAGG ACAGCTGAGG ATGGCTAAGA GGGCGTGGAC
```

Kpn I **Kozak**

```
WT-cow      ---------- ---------- ---------- ---------- ----------
Trans-cow   CCGCACAATC GCCACCGCCT AGAGAGGAAG CACCCGGTAC CGCCACCATG
pDonor-1    CCGCACAATC GCCACCGCCT AGAGAGGAAG CACCCGGTAC CGCCACCATG
pDonor-2    CCGCACAATC GCCACCGCCT AGAGAGGAAG CACCCGGTAC CGCCACCATG

WT-cow      ---------- ---------- ---------- ---------- ----------
Trans-cow   TCAGGTGACA CGGGCCCCCC AAAGCAGGGA GGGACCAGAT ATGGCTCCAT
pDonor-1    TCAGGTGACA CGGGCCCCCC AAAGCAGGGA GGGACCAGAT ATGGCTCCAT
pDonor-2    TCAGGTGACA CGGGCCCCCC AAAGCAGGGA GGGACCAGAT ATGGCTCCAT
```

NRAMP1 **coding sequence (1647bp)**

```
WT-cow      ---------- ---------- ---------- ---------- ----------
Trans-cow   CTCCAGCCCA CCCAGTCCAG AGCCACAGCA AGCACCTCCC GGAGGGACCT
pDonor-1    CTCCAGCCCA CCCAGTCCAG AGCCACAGCA AGCACCTCCC GGAGGGACCT
pDonor-2    CTCCAGCCCA CCCAGTCCAG AGCCACAGCA AGCACCTCCC GGAGGGACCT

WT-cow      ---------- ---------- ---------- ---------- ----------
Trans-cow   ACCTAAGTGA GAAGATCCCC ATTCCGGATA CAGAATCGGG TGCATTCAGC
pDonor-1    ACCTAAGTGA GAAGATCCCC ATTCCGGATA CAGAATCGGG TGCATTCAGC
pDonor-2    ACCTAAGTGA GAAGATCCCC ATTCCGGATA CAGAATCGGG TGCATTCAGC

WT-cow      ---------- ---------- ---------- ---------- ----------
Trans-cow   CTGCGGAAGC TGTGGGCCTT CACGGGGCCT GGATTCCTCA TGAGCATCGC
pDonor-1    CTGCGGAAGC TGTGGGCCTT CACGGGGCCT GGATTCCTCA TGAGCATCGC
pDonor-2    CTGCGGAAGC TGTGGGCCTT CACGGGGCCT GGATTCCTCA TGAGCATCGC
```

```
WT-cow        ---------- ---------- ---------- ---------- ----------
Trans-cow     ATTCCTGGAC CCAGGAAACA TTGAGTCGGA TCTTCAGGCT GGGGCTGTGG
pDonor-1      ATTCCTGGAC CCAGGAAACA TTGAGTCGGA TCTTCAGGCT GGGGCTGTGG
pDonor-2      ATTCCTGGAC CCAGGAAACA TTGAGTCGGA TCTTCAGGCT GGGGCTGTGG

WT-cow        ---------- ---------- ---------- ---------- ----------
Trans-cow     CTGGATTCAA ACTGCTCTGG GTGCTGCTGT GGGCCACAGT GTTGGGCTTG
pDonor-1      CTGGATTCAA ACTGCTCTGG GTGCTGCTGT GGGCCACAGT GTTGGGCTTG
pDonor-2      CTGGATTCAA ACTGCTCTGG GTGCTGCTGT GGGCCACAGT GTTGGGCTTG

WT-cow        ---------- ---------- ---------- ---------- ----------
Trans-cow     CTTTGCCAGC GACTGGCTGC CCGGCTGGGC GTGGTGACAG GCAAGGACTT
pDonor-1      CTTTGCCAGC GACTGGCTGC CCGGCTGGGC GTGGTGACAG GCAAGGACTT
pDonor-2      CTTTGCCAGC GACTGGCTGC CCGGCTGGGC GTGGTGACAG GCAAGGACTT

WT-cow        ---------- ---------- ---------- ---------- ----------
Trans-cow     GGGCGAGGTC TGCCATCTCT ACTACCCTAA GGTGCCCCGC ATTCTCCTCT
pDonor-1      GGGCGAGGTC TGCCATCTCT ACTACCCTAA GGTGCCCCGC ATTCTCCTCT
pDonor-2      GGGCGAGGTC TGCCATCTCT ACTACCCTAA GGTGCCCCGC ATTCTCCTCT
```

***NRAMP1* coding sequence (1647bp)**

```
WT-cow        ---------- ---------- ---------- ---------- ----------
Trans-cow     GGCTGACCAT CGAGCTAGCC ATCGTGGGCT CAGACATGCA GGAAGTCATT
pDonor-1      GGCTGACCAT CGAGCTAGCC ATCGTGGGCT CAGACATGCA GGAAGTCATT
pDonor-2      GGCTGACCAT CGAGCTAGCC ATCGTGGGCT CAGACATGCA GGAAGTCATT

WT-cow        ---------- ---------- ---------- ---------- ----------
Trans-cow     GGCACAGCTA TTGCATTCAG TCTGCTCTCC GCCGGACGAA TCCCACTCTG
pDonor-1      GGCACAGCTA TTGCATTCAG TCTGCTCTCC GCCGGACGAA TCCCACTCTG
pDonor-2      GGCACAGCTA TTGCATTCAG TCTGCTCTCC GCCGGACGAA TCCCACTCTG

WT-cow        ---------- ---------- ---------- ---------- ----------
Trans-cow     GGGTGGTGTC CTCATCACCG TCGTGGACAC TTTCTTCTTC CTCTTCCTCG
pDonor-1      GGGTGGTGTC CTCATCACCG TCGTGGACAC TTTCTTCTTC CTCTTCCTCG
pDonor-2      GGGTGGTGTC CTCATCACCG TCGTGGACAC TTTCTTCTTC CTCTTCCTCG

WT-cow        ---------- ---------- ---------- ---------- ----------
Trans-cow     ATAACTACGG GTTGCGGAAG CTGGAAGCCT TTTTTGGATT TCTTATTACC
pDonor-1      ATAACTACGG GTTGCGGAAG CTGGAAGCCT TTTTTGGATT TCTTATTACC
pDonor-2      ATAACTACGG GTTGCGGAAG CTGGAAGCCT TTTTTGGATT TCTTATTACC

WT-cow        ---------- ---------- ---------- ---------- ----------
Trans-cow     ATAATGGCCT TGACCTTCGG CTATGAGTAC GTGGTGGCTC AGCCTGCTCA
pDonor-1      ATAATGGCCT TGACCTTCGG CTATGAGTAC GTGGTGGCTC AGCCTGCTCA
pDonor-2      ATAATGGCCT TGACCTTCGG CTATGAGTAC GTGGTGGCTC AGCCTGCTCA
```

```
WT-cow      ---------- ---------- ---------- ---------- ----------
Trans-cow   GGGAGCATTG CTTCAGGGCC TGTTCCTGCC CTCGTGCCCA GGCTGTGGCC
pDonor-1    GGGAGCATTG CTTCAGGGCC TGTTCCTGCC CTCGTGCCCA GGCTGTGGCC
pDonor-2    GGGAGCATTG CTTCAGGGCC TGTTCCTGCC CTCGTGCCCA GGCTGTGGCC

WT-cow      ---------- ---------- ---------- ---------- ----------
Trans-cow   AGCCCGAGCT GCTGCAAGCC GTGGGCATCA TTGGCGCCAT CATCATGCCC
pDonor-1    AGCCCGAGCT GCTGCAAGCC GTGGGCATCA TTGGCGCCAT CATCATGCCC
pDonor-2    AGCCCGAGCT GCTGCAAGCC GTGGGCATCA TTGGCGCCAT CATCATGCCC

WT-cow      ---------- ---------- ---------- ---------- ----------
Trans-cow   CACAACATCT ACCTGCATTC CTCCCTGGTC AAGTCTCGAG AGGTAGACCG
pDonor-1    CACAACATCT ACCTGCATTC CTCCCTGGTC AAGTCTCGAG AGGTAGACCG
pDonor-2    CACAACATCT ACCTGCATTC CTCCCTGGTC AAGTCTCGAG AGGTAGACCG

WT-cow      ---------- ---------- ---------- ---------- ----------
Trans-cow   GTCCCGGCGG GCGGACATCC GAGAGGCCAA CATGTACTTC CTGATTGAAG
pDonor-1    GTCCCGGCGG GCGGACATCC GAGAGGCCAA CATGTACTTC CTGATTGAAG
pDonor-2    GTCCCGGCGG GCGGACATCC GAGAGGCCAA CATGTACTTC CTGATTGAAG
```

***NRAMP1* coding sequence (1647bp)**

```
WT-cow      ---------- ---------- ---------- ---------- ----------
Trans-cow   CCACCATCGC CCTGTCTGTC TCCTTCCTCA TCAACCTGTT TGTCATGGCT
pDonor-1    CCACCATCGC CCTGTCTGTC TCCTTCCTCA TCAACCTGTT TGTCATGGCT
pDonor-2    CCACCATCGC CCTGTCTGTC TCCTTCCTCA TCAACCTGTT TGTCATGGCT

WT-cow      ---------- ---------- ---------- ---------- ----------
Trans-cow   GTCTTTGGGC AAGCCTTCTA CAAGCAAACC AACCAGGCTG CGTTCAACAT
pDonor-1    GTCTTTGGGC AAGCCTTCTA CAAGCAAACC AACCAGGCTG CGTTCAACAT
pDonor-2    GTCTTTGGGC AAGCCTTCTA CAAGCAAACC AACCAGGCTG CGTTCAACAT

WT-cow      ---------- ---------- ---------- ---------- ----------
Trans-cow   CTGTGCCGAC AGCAGCCTCC ACGACTACGC GCCGATCTTT CCCAGGAACA
pDonor-1    CTGTGCCGAC AGCAGCCTCC ACGACTACGC GCCGATCTTT CCCAGGAACA
pDonor-2    CTGTGCCGAC AGCAGCCTCC ACGACTACGC GCCGATCTTT CCCAGGAACA

WT-cow      ---------- ---------- ---------- ---------- ----------
Trans-cow   ACCTGACCGT GGCAGTGGAC ATTTACCAAG GAGGCGTGAT CCTGGGCTGC
pDonor-1    ACCTGACCGT GGCAGTGGAC ATTTACCAAG GAGGCGTGAT CCTGGGCTGC
pDonor-2    ACCTGACCGT GGCAGTGGAC ATTTACCAAG GAGGCGTGAT CCTGGGCTGC

WT-cow      ---------- ---------- ---------- ---------- ----------
Trans-cow   CTCTTTGGTC CTCCAGCCCT GTACATCTGG GCCGTGGGTC TCCTGGCTGC
pDonor-1    CTCTTTGGTC CTCCAGCCCT GTACATCTGG GCCGTGGGTC TCCTGGCTGC
pDonor-2    CTCTTTGGTC CTCCAGCCCT GTACATCTGG GCCGTGGGTC TCCTGGCTGC
```

```
WT-cow       ---------- ---------- ---------- ---------- ----------
Trans-cow    TGGGCAGAGC TCCACCATGA CCGGCACCTA CGCGGGACAG TTTGTGATGG
pDonor-1     TGGGCAGAGC TCCACCATGA CCGGCACCTA CGCGGGACAG TTTGTGATGG
pDonor-2     TGGGCAGAGC TCCACCATGA CCGGCACCTA CGCGGGACAG TTTGTGATGG

WT-cow       ---------- ---------- ---------- ---------- ----------
Trans-cow    AGGGCTTCCT GAAGCTGCGG TGGTCACGCT TCGCCCGAGT CCTGCTCACT
pDonor-1     AGGGCTTCCT GAAGCTGCGG TGGTCACGCT TCGCCCGAGT CCTGCTCACT
pDonor-2     AGGGCTTCCT GAAGCTGCGG TGGTCACGCT TCGCCCGAGT CCTGCTCACT

WT-cow       ---------- ---------- ---------- ---------- ----------
Trans-cow    CGCTCCTGCG CCATCCTGCC CACTGTGCTC CTGGCTGTCT TCAGGGACTT
pDonor-1     CGCTCCTGCG CCATCCTGCC CACTGTGCTC CTGGCTGTCT TCAGGGACTT
pDonor-2     CGCTCCTGCG CCATCCTGCC CACTGTGCTC CTGGCTGTCT TCAGGGACTT

WT-cow       ---------- ---------- ---------- ---------- ----------
Trans-cow    GCGGGACCTG TCAGGCCTCA ACGACCTGCT CAATGTGCTG CAGAGCCTGC
pDonor-1     GCGGGACCTG TCAGGCCTCA ACGACCTGCT CAATGTGCTG CAGAGCCTGC
pDonor-2     GCGGGACCTG TCAGGCCTCA ACGACCTGCT CAATGTGCTG CAGAGCCTGC
```

***NRAMP1* coding sequence (1647bp)**

```
WT-cow       ---------- ---------- ---------- ---------- ----------
Trans-cow    TGCTTCCCTT CGCTGTGCTG CCCATCCTCA CCTTCACCAG CATGCCCGCC
pDonor-1     TGCTTCCCTT CGCTGTGCTG CCCATCCTCA CCTTCACCAG CATGCCCGCC
pDonor-2     TGCTTCCCTT CGCTGTGCTG CCCATCCTCA CCTTCACCAG CATGCCCGCC

WT-cow       ---------- ---------- ---------- ---------- ----------
Trans-cow    CTGATGCAGG AGTTTGCCAA TGGCCTGGTG AGCAAAGTTA TCACTTCCTC
pDonor-1     CTGATGCAGG AGTTTGCCAA TGGCCTGGTG AGCAAAGTTA TCACTTCCTC
pDonor-2     CTGATGCAGG AGTTTGCCAA TGGCCTGGTG AGCAAAGTTA TCACTTCCTC

WT-cow       ---------- ---------- ---------- ---------- ----------
Trans-cow    CATCATGGTG CTGGTCTGCG CCGTCAACCT TTACTTCGTG ATCAGCTACT
pDonor-1     CATCATGGTG CTGGTCTGCG CCGTCAACCT TTACTTCGTG ATCAGCTACT
pDonor-2     CATCATGGTG CTGGTCTGCG CCGTCAACCT TTACTTCGTG ATCAGCTACT

WT-cow       ---------- ---------- ---------- ---------- ----------
Trans-cow    TGCCCAGCCT CCCCCACCCT GCCTACTTCA GCCTTGTAGC ACTGCTGGCC
pDonor-1     TGCCCAGCCT CCCCCACCCT GCCTACTTCA GCCTTGTAGC ACTGCTGGCC
pDonor-2     TGCCCAGCCT CCCCCACCCT GCCTACTTCA GCCTTGTAGC ACTGCTGGCC

WT-cow       ---------- ---------- ---------- ---------- ----------
Trans-cow    GCAGCCTACC TGGGCCTCAC CACTTACCTG GTCTGGACCT GTCTCATCAC
pDonor-1     GCAGCCTACC TGGGCCTCAC CACTTACCTG GTCTGGACCT GTCTCATCAC
pDonor-2     GCAGCCTACC TGGGCCTCAC CACTTACCTG GTCTGGACCT GTCTCATCAC
```

```
WT-cow       ---------- ---------- ---------- ---------- ----------
Trans-cow    CCAGGGAGCC ACTCTTCTGG CCCACAGTTC CCACCAACGC TTCCTGTATG
pDonor-1     CCAGGGAGCC ACTCTTCTGG CCCACAGTTC CCACCAACGC TTCCTGTATG
pDonor-2     CCAGGGAGCC ACTCTTCTGG CCCACAGTTC CCACCAACGC TTCCTGTATG
```

NRAMP1 **coding sequence (1647bp)** → **BamH I**

```
WT-cow       ---------- ---------- ---------- ---------- ----------
Trans-cow    GGCTTCCTGA AGAGGATCAG GAGAAGGGGA GGACCTCGGG ATGAGGATCC
pDonor-1     GGCTTCCTGA AGAGGATCAG GAGAAGGGGA GGACCTCGGG ATGAGGATCC
pDonor-2     GGCTTCCTGA AGAGGATCAG GAGAAGGGGA GGACCTCGGG ATGAGGATCC

WT-cow       ---------- ---------- ---------- ---------- ----------
Trans-cow    ACCGGATCTA GATAACTGAT CATAATCAGC CATACCACAT TTGTAGAGGT
pDonor-1     ACCGGATCTA GATAACTGAT CATAATCAGC CATACCACAT TTGTAGAGGT
pDonor-2     ACCGGATCTA GATAACTGAT CATAATCAGC CATACCACAT TTGTAGAGGT

WT-cow       ---------- ---------- ---------- ---------- ----------
Trans-cow    TTTACTTGCT TTAAAAAACC TCCCACACCT CCCCCTGAAC CTGAAACATA
pDonor-1     TTTACTTGCT TTAAAAAACC TCCCACACCT CCCCCTGAAC CTGAAACATA
pDonor-2     TTTACTTGCT TTAAAAAACC TCCCACACCT CCCCCTGAAC CTGAAACATA
```

SV40 polyA (245bp)

```
WT-cow       ---------- ---------- ---------- ---------- ----------
Trans-cow    AAATGAATGC AATTGTTGTT GTTAACTTGT TTATTGCAGC TTATAATGGT
pDonor-1     AAATGAATGC AATTGTTGTT GTTAACTTGT TTATTGCAGC TTATAATGGT
pDonor-2     AAATGAATGC AATTGTTGTT GTTAACTTGT TTATTGCAGC TTATAATGGT

WT-cow       ---------- ---------- ---------- ---------- ----------
Trans-cow    TACAAATAAA GCAATAGCAT CACAAATTTC ACAAATAAAG CATTTTTTTC
pDonor-1     TACAAATAAA GCAATAGCAT CACAAATTTC ACAAATAAAG CATTTTTTTC
pDonor-2     TACAAATAAA GCAATAGCAT CACAAATTTC ACAAATAAAG CATTTTTTTC

WT-cow       ---------- ---------- ---------- ---------- ----------
Trans-cow    ACTGCATTCT AGTTGTGGTT TGTCCAAACT CATCAATGTA TCTTAACGCG
pDonor-1     ACTGCATTCT AGTTGTGGTT TGTCCAAACT CATCAATGTA TCTTAACGCG
pDonor-2     ACTGCATTCT AGTTGTGGTT TGTCCAAACT CATCAATGTA TCTTAACGCG
```

Spe I **LoxP (34bp)**

```
WT-cow       ---------- ---------- ---------- ---------- ----------
Trans-cow    TACTAGTATA ACTTCGTATA GCATACATTA TACGAAGTTA TTGCGTTATC
pDonor-1     TACTAGTATA ACTTCGTATA GCATACATTA TACGAAGTTA TTGCGTTATC
pDonor-2     TACTAGTATA ACTTCGTATA GCATACATTA TACGAAGTTA TTGCGTTATC
```

pEF1α-eGFP-P2A-Puro cassette (3062bp)

```
WT-cow       ---------- ---------- ---------- ---------- ----------
Trans-cow    CCCTGATTCT GTGGATAACC GTATTACCGC CATGCATTAG TTATTAATAT
pDonor-1     CCCTGATTCT GTGGATAACC GTATTACCGC CATGCATTAG TTATTAATAT
pDonor-2     CCCTGATTCT GTGGATAACC GTATTACCGC CATGCATTAG TTATTAATAT
```

```
WT-cow      ---------- ---------- ---------- ---------- ----------
Trans-cow   CCCAGGGACC GTCGTTAAAC TCCCACTAAC GTAGAACCCA GAGATCGCTG
pDonor-1    CCCAGGGACC GTCGTTAAAC TCCCACTAAC GTAGAACCCA GAGATCGCTG
pDonor-2    CCCAGGGACC GTCGTTAAAC TCCCACTAAC GTAGAACCCA GAGATCGCTG

WT-cow      ---------- ---------- ---------- ---------- ----------
Trans-cow   CGTTCCCGCC CCCTCACCCG CCCGCTCTCG TCATCACTGA GGTGGAGAAG
pDonor-1    CGTTCCCGCC CCCTCACCCG CCCGCTCTCG TCATCACTGA GGTGGAGAAG
pDonor-2    CGTTCCCGCC CCCTCACCCG CCCGCTCTCG TCATCACTGA GGTGGAGAAG

WT-cow      ---------- ---------- ---------- ---------- ----------
Trans-cow   AGCATGCGTG AGGCTCCGGT GCCCGTCAGT GGGCAGAGCG CACATCGCCC
pDonor-1    AGCATGCGTG AGGCTCCGGT GCCCGTCAGT GGGCAGAGCG CACATCGCCC
pDonor-2    AGCATGCGTG AGGCTCCGGT GCCCGTCAGT GGGCAGAGCG CACATCGCCC

WT-cow      ---------- ---------- ---------- ---------- ----------
Trans-cow   ACAGTCCCCG AGAAGTTGGG GGGAGGGGTC GGCAATTGAA CCGGTGCCTA
pDonor-1    ACAGTCCCCG AGAAGTTGGG GGGAGGGGTC GGCAATTGAA CCGGTGCCTA
pDonor-2    ACAGTCCCCG AGAAGTTGGG GGGAGGGGTC GGCAATTGAA CCGGTGCCTA
```

pEF1α-eGFP-P2A-Puro cassette (3062bp)

```
WT-cow      ---------- ---------- ---------- ---------- ----------
Trans-cow   GAGAAGGTGG CGCGGGGTAA ACTGGGAAAG TGATGTCGTG TACTGGCTCC
pDonor-1    GAGAAGGTGG CGCGGGGTAA ACTGGGAAAG TGATGTCGTG TACTGGCTCC
pDonor-2    GAGAAGGTGG CGCGGGGTAA ACTGGGAAAG TGATGTCGTG TACTGGCTCC

WT-cow      ---------- ---------- ---------- ---------- ----------
Trans-cow   GCCTTTTTCC CGAGGGTGGG GGAGAACCGT ATATAAGTGC AGTAGTCGCC
pDonor-1    GCCTTTTTCC CGAGGGTGGG GGAGAACCGT ATATAAGTGC AGTAGTCGCC
pDonor-2    GCCTTTTTCC CGAGGGTGGG GGAGAACCGT ATATAAGTGC AGTAGTCGCC

WT-cow      ---------- ---------- ---------- ---------- ----------
Trans-cow   GTGAACGTTC TTTTTCGCAA CGGGTTTGCC GCCAGAACAC AGGTAAGTGC
pDonor-1    GTGAACGTTC TTTTTCGCAA CGGGTTTGCC GCCAGAACAC AGGTAAGTGC
pDonor-2    GTGAACGTTC TTTTTCGCAA CGGGTTTGCC GCCAGAACAC AGGTAAGTGC

WT-cow      ---------- ---------- ---------- ---------- ----------
Trans-cow   CGTGTGTGGT TCCCGCGGGC CTGGCCTCTT TACGGGTTAT GGCCCTTGCG
pDonor-1    CGTGTGTGGT TCCCGCGGGC CTGGCCTCTT TACGGGTTAT GGCCCTTGCG
pDonor-2    CGTGTGTGGT TCCCGCGGGC CTGGCCTCTT TACGGGTTAT GGCCCTTGCG

WT-cow      ---------- ---------- ---------- ---------- ----------
Trans-cow   TGCCTTGAAT TACTTCCACG CCCCTGGCTG CAGTACGTGA TTCTTGATCC
pDonor-1    TGCCTTGAAT TACTTCCACG CCCCTGGCTG CAGTACGTGA TTCTTGATCC
pDonor-2    TGCCTTGAAT TACTTCCACG CCCCTGGCTG CAGTACGTGA TTCTTGATCC
```

```
WT-cow       ---------- ---------- ---------- ---------- ----------
Trans-cow    CGAGCTTCGG GTTGGAAGTG GGTGGGAGAG TTCGAGGCCT TGCGCTTAAG
pDonor-1     CGAGCTTCGG GTTGGAAGTG GGTGGGAGAG TTCGAGGCCT TGCGCTTAAG
pDonor-2     CGAGCTTCGG GTTGGAAGTG GGTGGGAGAG TTCGAGGCCT TGCGCTTAAG

WT-cow       ---------- ---------- ---------- ---------- ----------
Trans-cow    GAGCCCCTTC GCCTCGTGCT TGAGTTGAGG CCTGGCTTGG GCGCTGGGGC
pDonor-1     GAGCCCCTTC GCCTCGTGCT TGAGTTGAGG CCTGGCTTGG GCGCTGGGGC
pDonor-2     GAGCCCCTTC GCCTCGTGCT TGAGTTGAGG CCTGGCTTGG GCGCTGGGGC

WT-cow       ---------- ---------- ---------- ---------- ----------
Trans-cow    CGCCGCGTGC GAATCTGGTG GCACCTTCGC GCCTGTCTCG CTGCTTTCGA
pDonor-1     CGCCGCGTGC GAATCTGGTG GCACCTTCGC GCCTGTCTCG CTGCTTTCGA
pDonor-2     CGCCGCGTGC GAATCTGGTG GCACCTTCGC GCCTGTCTCG CTGCTTTCGA

WT-cow       ---------- ---------- ---------- ---------- ----------
Trans-cow    TAAGTCTCTA GCCATTTAAA ATTTTTGATG ACCTGCTGCG ACGCTTTTTT
pDonor-1     TAAGTCTCTA GCCATTTAAA ATTTTTGATG ACCTGCTGCG ACGCTTTTTT
pDonor-2     TAAGTCTCTA GCCATTTAAA ATTTTTGATG ACCTGCTGCG ACGCTTTTTT
```

pEF1α-eGFP-P2A-Puro cassette (3062bp)

```
WT-cow       ---------- ---------- ---------- ---------- ----------
Trans-cow    TCTGGCAAGA TAGTCTTGTA AATGCGGGCC AAGATCTGCA CACTGGTATT
pDonor-1     TCTGGCAAGA TAGTCTTGTA AATGCGGGCC AAGATCTGCA CACTGGTATT
pDonor-2     TCTGGCAAGA TAGTCTTGTA AATGCGGGCC AAGATCTGCA CACTGGTATT

WT-cow       ---------- ---------- ---------- ---------- ----------
Trans-cow    TCGGTTTTTG GGGCCGCGGG CGGCGACGGG GCCCGTGCGT CCCAGCGCAC
pDonor-1     TCGGTTTTTG GGGCCGCGGG CGGCGACGGG GCCCGTGCGT CCCAGCGCAC
pDonor-2     TCGGTTTTTG GGGCCGCGGG CGGCGACGGG GCCCGTGCGT CCCAGCGCAC

WT-cow       ---------- ---------- ---------- ---------- ----------
Trans-cow    ATGTTCGGCG AGGCGGGGCC TGCGAGCGCG GCCACCGAGA ATCGGACGGG
pDonor-1     ATGTTCGGCG AGGCGGGGCC TGCGAGCGCG GCCACCGAGA ATCGGACGGG
pDonor-2     ATGTTCGGCG AGGCGGGGCC TGCGAGCGCG GCCACCGAGA ATCGGACGGG

WT-cow       ---------- ---------- ---------- ---------- ----------
Trans-cow    GGTAGTCTCA AGCTGGCCGG CCTGCTCTGG TGCCTGGCCT CGCGCCGCCG
pDonor-1     GGTAGTCTCA AGCTGGCCGG CCTGCTCTGG TGCCTGGCCT CGCGCCGCCG
pDonor-2     GGTAGTCTCA AGCTGGCCGG CCTGCTCTGG TGCCTGGCCT CGCGCCGCCG

WT-cow       ---------- ---------- ---------- ---------- ----------
Trans-cow    TGTATCGCCC CGCCCTGGGC GGCAAGGCTG GCCCGGTCGG CACCAGTTGC
pDonor-1     TGTATCGCCC CGCCCTGGGC GGCAAGGCTG GCCCGGTCGG CACCAGTTGC
pDonor-2     TGTATCGCCC CGCCCTGGGC GGCAAGGCTG GCCCGGTCGG CACCAGTTGC
```

```
WT-cow      ---------- ---------- ---------- ---------- ----------
Trans-cow   GTGAGCGGAA AGATGGCCGC TTCCCGGCCC TGCTGCAGGG AGCTCAAAAT
pDonor-1    GTGAGCGGAA AGATGGCCGC TTCCCGGCCC TGCTGCAGGG AGCTCAAAAT
pDonor-2    GTGAGCGGAA AGATGGCCGC TTCCCGGCCC TGCTGCAGGG AGCTCAAAAT

WT-cow      ---------- ---------- ---------- ---------- ----------
Trans-cow   GGAGGACGCG GCGCTCGGGA GAGCGGGCGG GTGAGTCACC CACACAAAGG
pDonor-1    GGAGGACGCG GCGCTCGGGA GAGCGGGCGG GTGAGTCACC CACACAAAGG
pDonor-2    GGAGGACGCG GCGCTCGGGA GAGCGGGCGG GTGAGTCACC CACACAAAGG

WT-cow      ---------- ---------- ---------- ---------- ----------
Trans-cow   AAAAGGGCCT TTCCGTCCTC AGCCGTCGCT TCATGTGACT CCACGGAGTA
pDonor-1    AAAAGGGCCT TTCCGTCCTC AGCCGTCGCT TCATGTGACT CCACGGAGTA
pDonor-2    AAAAGGGCCT TTCCGTCCTC AGCCGTCGCT TCATGTGACT CCACGGAGTA

WT-cow      ---------- ---------- ---------- ---------- ----------
Trans-cow   CCGGGCGCCG TCCAGGCACC TCGATTAGTT CTCGAGCTTT TGGAGTACGT
pDonor-1    CCGGGCGCCG TCCAGGCACC TCGATTAGTT CTCGAGCTTT TGGAGTACGT
pDonor-2    CCGGGCGCCG TCCAGGCACC TCGATTAGTT CTCGAGCTTT TGGAGTACGT
```

pEF1α-eGFP-P2A-Puro cassette (3062bp)

```
WT-cow      ---------- ---------- ---------- ---------- ----------
Trans-cow   CGTCTTTAGG TTGGGGGGAG GGGTTTTATG CGATGGAGTT TCCCCACACT
pDonor-1    CGTCTTTAGG TTGGGGGGAG GGGTTTTATG CGATGGAGTT TCCCCACACT
pDonor-2    CGTCTTTAGG TTGGGGGGAG GGGTTTTATG CGATGGAGTT TCCCCACACT

WT-cow      ---------- ---------- ---------- ---------- ----------
Trans-cow   GAGTGGGTGG AGACTGAAGT TAGGCCAGCT TGGCACTTGA TGTAATTCTC
pDonor-1    GAGTGGGTGG AGACTGAAGT TAGGCCAGCT TGGCACTTGA TGTAATTCTC
pDonor-2    GAGTGGGTGG AGACTGAAGT TAGGCCAGCT TGGCACTTGA TGTAATTCTC

WT-cow      ---------- ---------- ---------- ---------- ----------
Trans-cow   CTTGGAATTT GCCCTTTTTG AGTTTGGATC TTGGTTCATT CTCAAGCCTC
pDonor-1    CTTGGAATTT GCCCTTTTTG AGTTTGGATC TTGGTTCATT CTCAAGCCTC
pDonor-2    CTTGGAATTT GCCCTTTTTG AGTTTGGATC TTGGTTCATT CTCAAGCCTC

WT-cow      ---------- ---------- ---------- ---------- ----------
Trans-cow   AGACAGTGGT TCAAAGTTTT TTTCTTCCAT TTCAGGTGTC GTGAGCTAGC
pDonor-1    AGACAGTGGT TCAAAGTTTT TTTCTTCCAT TTCAGGTGTC GTGAGCTAGC
pDonor-2    AGACAGTGGT TCAAAGTTTT TTTCTTCCAT TTCAGGTGTC GTGAGCTAGC

WT-cow      ---------- ---------- ---------- ---------- ----------
Trans-cow   GCTACCGGTC GCCACCATGG TGAGCAAGGG CGAGGAGCTG TTCACCGGGG
pDonor-1    GCTACCGGTC GCCACCATGG TGAGCAAGGG CGAGGAGCTG TTCACCGGGG
pDonor-2    GCTACCGGTC GCCACCATGG TGAGCAAGGG CGAGGAGCTG TTCACCGGGG
```

```
WT-cow        ---------- ---------- ---------- ---------- ----------
Trans-cow     TGGTGCCCAT CCTGGTCGAG CTGGACGGCG ACGTAAACGG CCACAAGTTC
pDonor-1      TGGTGCCCAT CCTGGTCGAG CTGGACGGCG ACGTAAACGG CCACAAGTTC
pDonor-2      TGGTGCCCAT CCTGGTCGAG CTGGACGGCG ACGTAAACGG CCACAAGTTC

WT-cow        ---------- ---------- ---------- ---------- ----------
Trans-cow     AGCGTGTCCG GCGAGGGCGA GGGCGATGCC ACCTACGGCA AGCTGACCCT
pDonor-1      AGCGTGTCCG GCGAGGGCGA GGGCGATGCC ACCTACGGCA AGCTGACCCT
pDonor-2      AGCGTGTCCG GCGAGGGCGA GGGCGATGCC ACCTACGGCA AGCTGACCCT

WT-cow        ---------- ---------- ---------- ---------- ----------
Trans-cow     GAAGTTCATC TGCACCACCG GCAAGCTGCC CGTGCCCTGG CCCACCCTCG
pDonor-1      GAAGTTCATC TGCACCACCG GCAAGCTGCC CGTGCCCTGG CCCACCCTCG
pDonor-2      GAAGTTCATC TGCACCACCG GCAAGCTGCC CGTGCCCTGG CCCACCCTCG

WT-cow        ---------- ---------- ---------- ---------- ----------
Trans-cow     TGACCACCCT GACCTACGGC GTGCAGTGCT TCAGCCGCTA CCCCGACCAC
pDonor-1      TGACCACCCT GACCTACGGC GTGCAGTGCT TCAGCCGCTA CCCCGACCAC
pDonor-2      TGACCACCCT GACCTACGGC GTGCAGTGCT TCAGCCGCTA CCCCGACCAC
```

pEF1α-eGFP-P2A-Puro cassette (3062bp)

```
WT-cow        ---------- ---------- ---------- ---------- ----------
Trans-cow     ATGAAGCAGC ACGACTTCTT CAAGTCCGCC ATGCCCGAAG GCTACGTCCA
pDonor-1      ATGAAGCAGC ACGACTTCTT CAAGTCCGCC ATGCCCGAAG GCTACGTCCA
pDonor-2      ATGAAGCAGC ACGACTTCTT CAAGTCCGCC ATGCCCGAAG GCTACGTCCA

WT-cow        ---------- ---------- ---------- ---------- ----------
Trans-cow     GGAGCGCACC ATCTTCTTCA AGGACGACGG CAACTACAAG ACCCGCGCCG
pDonor-1      GGAGCGCACC ATCTTCTTCA AGGACGACGG CAACTACAAG ACCCGCGCCG
pDonor-2      GGAGCGCACC ATCTTCTTCA AGGACGACGG CAACTACAAG ACCCGCGCCG

WT-cow        ---------- ---------- ---------- ---------- ----------
Trans-cow     AGGTGAAGTT CGAGGGCGAC ACCCTGGTGA ACCGCATCGA GCTGAAGGGC
pDonor-1      AGGTGAAGTT CGAGGGCGAC ACCCTGGTGA ACCGCATCGA GCTGAAGGGC
pDonor-2      AGGTGAAGTT CGAGGGCGAC ACCCTGGTGA ACCGCATCGA GCTGAAGGGC

WT-cow        ---------- ---------- ---------- ---------- ----------
Trans-cow     ATCGACTTCA AGGAGGACGG CAACATCCTG GGGCACAAGC TGGAGTACAA
pDonor-1      ATCGACTTCA AGGAGGACGG CAACATCCTG GGGCACAAGC TGGAGTACAA
pDonor-2      ATCGACTTCA AGGAGGACGG CAACATCCTG GGGCACAAGC TGGAGTACAA

WT-cow        ---------- ---------- ---------- ---------- ----------
Trans-cow     CTACAACAGC CACAACGTCT ATATCATGGC CGACAAGCAG AAGAACGGCA
pDonor-1      CTACAACAGC CACAACGTCT ATATCATGGC CGACAAGCAG AAGAACGGCA
pDonor-2      CTACAACAGC CACAACGTCT ATATCATGGC CGACAAGCAG AAGAACGGCA
```

```
WT-cow       ---------- ---------- ---------- ---------- ----------
Trans-cow    TCAAGGTGAA CTTCAAGATC CGCCACAACA TCGAGGACGG CAGCGTGCAG
pDonor-1     TCAAGGTGAA CTTCAAGATC CGCCACAACA TCGAGGACGG CAGCGTGCAG
pDonor-2     TCAAGGTGAA CTTCAAGATC CGCCACAACA TCGAGGACGG CAGCGTGCAG
```

```
WT-cow       ---------- ---------- ---------- ---------- ----------
Trans-cow    CTCGCCGACC ACTACCAGCA GAACACCCCC ATCGGCGACG GCCCCGTGCT
pDonor-1     CTCGCCGACC ACTACCAGCA GAACACCCCC ATCGGCGACG GCCCCGTGCT
pDonor-2     CTCGCCGACC ACTACCAGCA GAACACCCCC ATCGGCGACG GCCCCGTGCT
```

```
WT-cow       ---------- ---------- ---------- ---------- ----------
Trans-cow    GCTGCCCGAC AACCACTACC TGAGCACCCA GTCCGCCCTG AGCAAAGACC
pDonor-1     GCTGCCCGAC AACCACTACC TGAGCACCCA GTCCGCCCTG AGCAAAGACC
pDonor-2     GCTGCCCGAC AACCACTACC TGAGCACCCA GTCCGCCCTG AGCAAAGACC
```

```
WT-cow       ---------- ---------- ---------- ---------- ----------
Trans-cow    CCAACGAGAA GCGCGATCAC ATGGTCCTGC TGGAGTTCGT GACCGCCGCC
pDonor-1     CCAACGAGAA GCGCGATCAC ATGGTCCTGC TGGAGTTCGT GACCGCCGCC
pDonor-2     CCAACGAGAA GCGCGATCAC ATGGTCCTGC TGGAGTTCGT GACCGCCGCC
```

pEF1α-eGFP-P2A-Puro cassette (3062bp)

```
WT-cow       ---------- ---------- ---------- ---------- ----------
Trans-cow    GGGATCACTC TCGGCATGGA CGAGCTGTAC AAGGGATCCG GAGCCACGAA
pDonor-1     GGGATCACTC TCGGCATGGA CGAGCTGTAC AAGGGATCCG GAGCCACGAA
pDonor-2     GGGATCACTC TCGGCATGGA CGAGCTGTAC AAGGGATCCG GAGCCACGAA
```

Probe 2-primer-1F →

```
WT-cow       ---------- ---------- ---------- ---------- ----------
Trans-cow    CTTCTCTCTG TTAAAGCAAG CAGGAGACGT GGAAGAAAAC CCCGGTCCTA
pDonor-1     CTTCTCTCTG TTAAAGCAAG CAGGAGACGT GGAAGAAAAC CCCGGTCCTA
pDonor-2     CTTCTCTCTG TTAAAGCAAG CAGGAGACGT GGAAGAAAAC CCCGGTCCTA
```

```
WT-cow       ---------- ---------- ---------- ---------- ----------
Trans-cow    TGACCGAGTA CAAGCCCACG GTGCGCCTCG CCACCCGCGA CGACGTCCCC
pDonor-1     TGACCGAGTA CAAGCCCACG GTGCGCCTCG CCACCCGCGA CGACGTCCCC
pDonor-2     TGACCGAGTA CAAGCCCACG GTGCGCCTCG CCACCCGCGA CGACGTCCCC
```

```
WT-cow       ---------- ---------- ---------- ---------- ----------
Trans-cow    AGGGCCGTAC GCACCCTCGC CGCCGCGTTC GCCGACTACC CCGCCACGCG
pDonor-1     AGGGCCGTAC GCACCCTCGC CGCCGCGTTC GCCGACTACC CCGCCACGCG
pDonor-2     AGGGCCGTAC GCACCCTCGC CGCCGCGTTC GCCGACTACC CCGCCACGCG
```

```
WT-cow       ---------- ---------- ---------- ---------- ----------
Trans-cow    CCACACCGTC GATCCGGACC GCCACATCGA GCGGGTCACC GAGCTGCAAG
pDonor-1     CCACACCGTC GATCCGGACC GCCACATCGA GCGGGTCACC GAGCTGCAAG
pDonor-2     CCACACCGTC GATCCGGACC GCCACATCGA GCGGGTCACC GAGCTGCAAG
```

```
WT-cow      ---------- ---------- ---------- ---------- ----------
Trans-cow   AACTCTTCCT CACGCGCGTC GGGCTCGACA TCGGCAAGGT GTGGGTCGCG
pDonor-1    AACTCTTCCT CACGCGCGTC GGGCTCGACA TCGGCAAGGT GTGGGTCGCG
pDonor-2    AACTCTTCCT CACGCGCGTC GGGCTCGACA TCGGCAAGGT GTGGGTCGCG

WT-cow      ---------- ---------- ---------- ---------- ----------
Trans-cow   GACGACGGCG CCGCGGTGGC GGTCTGGACC ACGCCGGAGA GCGTCGAAGC
pDonor-1    GACGACGGCG CCGCGGTGGC GGTCTGGACC ACGCCGGAGA GCGTCGAAGC
pDonor-2    GACGACGGCG CCGCGGTGGC GGTCTGGACC ACGCCGGAGA GCGTCGAAGC
```

3’ Junction primer-F

```
WT-cow      ---------- ---------- ---------- ---------- ----------
Trans-cow   GGGGGCGGTG TTCGCCGAGA TCGGCCCGCG CATGGCCGAG TTGAGCGGTT
pDonor-1    GGGGGCGGTG TTCGCCGAGA TCGGCCCGCG CATGGCCGAG TTGAGCGGTT
pDonor-2    GGGGGCGGTG TTCGCCGAGA TCGGCCCGCG CATGGCCGAG TTGAGCGGTT

WT-cow      ---------- ---------- ---------- ---------- ----------
Trans-cow   CCCGGCTGGC CGCGCAGCAA CAGATGGAAG GCCTCCTGGC GCCGCACCGG
pDonor-1    CCCGGCTGGC CGCGCAGCAA CAGATGGAAG GCCTCCTGGC GCCGCACCGG
pDonor-2    CCCGGCTGGC CGCGCAGCAA CAGATGGAAG GCCTCCTGGC GCCGCACCGG
```

pEF1α-eGFP-P2A-Puro cassette (3062bp)

```
WT-cow      ---------- ---------- ---------- ---------- ----------
Trans-cow   CCCAAGGAGC CCGCGTGGTT CCTGGCCACC GTCGGCGTCT CGCCCGACCA
pDonor-1    CCCAAGGAGC CCGCGTGGTT CCTGGCCACC GTCGGCGTCT CGCCCGACCA
pDonor-2    CCCAAGGAGC CCGCGTGGTT CCTGGCCACC GTCGGCGTCT CGCCCGACCA

WT-cow      ---------- ---------- ---------- ---------- ----------
Trans-cow   CCAGGGCAAG GGTCTGGGCA GCGCCGTCGT GCTCCCCGGA GTGGAGGCGG
pDonor-1    CCAGGGCAAG GGTCTGGGCA GCGCCGTCGT GCTCCCCGGA GTGGAGGCGG
pDonor-2    CCAGGGCAAG GGTCTGGGCA GCGCCGTCGT GCTCCCCGGA GTGGAGGCGG

WT-cow      ---------- ---------- ---------- ---------- ----------
Trans-cow   CCGAGCGCGC CGGGGTGCCC GCCTTCCTGG AGACCTCCGC GCCCCGCAAC
pDonor-1    CCGAGCGCGC CGGGGTGCCC GCCTTCCTGG AGACCTCCGC GCCCCGCAAC
pDonor-2    CCGAGCGCGC CGGGGTGCCC GCCTTCCTGG AGACCTCCGC GCCCCGCAAC
```

Probe 2-primer-1R

```
WT-cow      ---------- ---------- ---------- ---------- ----------
Trans-cow   CTCCCCTTCT ACGAGCGGCT CGGCTTCACC GTCACCGCCG ACGTCGAGGT
pDonor-1    CTCCCCTTCT ACGAGCGGCT CGGCTTCACC GTCACCGCCG ACGTCGAGGT
pDonor-2    CTCCCCTTCT ACGAGCGGCT CGGCTTCACC GTCACCGCCG ACGTCGAGGT

WT-cow      ---------- ---------- ---------- ---------- ----------
Trans-cow   GCCCGAAGGA CCGCGCACCT GGTGCATGAC CCGCAAGCCC GGTGCCTGAA
pDonor-1    GCCCGAAGGA CCGCGCACCT GGTGCATGAC CCGCAAGCCC GGTGCCTGAA
pDonor-2    GCCCGAAGGA CCGCGCACCT GGTGCATGAC CCGCAAGCCC GGTGCCTGAA
```

```
WT-cow      ---------- ---------- ---------- ---------- ----------
Trans-cow   TCTAGGTGGT CAATCAACCG GTACCGCGGG CCCGGGATCC ACCGGATCTA
pDonor-1    TCTAGGTGGT CAATCAACCG GTACCGCGGG CCCGGGATCC ACCGGATCTA
pDonor-2    TCTAGGTGGT CAATCAACCG GTACCGCGGG CCCGGGATCC ACCGGATCTA

WT-cow      ---------- ---------- ---------- ---------- ----------
Trans-cow   GATAACTGAT CATAATCAGC CATACCACAT TTGTAGAGGT TTTACTTGCT
pDonor-1    GATAACTGAT CATAATCAGC CATACCACAT TTGTAGAGGT TTTACTTGCT
pDonor-2    GATAACTGAT CATAATCAGC CATACCACAT TTGTAGAGGT TTTACTTGCT

WT-cow      ---------- ---------- ---------- ---------- ----------
Trans-cow   TTAAAAAACC TCCCACACCT CCCCCTGAAC CTGAAACATA AAATGAATGC
pDonor-1    TTAAAAAACC TCCCACACCT CCCCCTGAAC CTGAAACATA AAATGAATGC
pDonor-2    TTAAAAAACC TCCCACACCT CCCCCTGAAC CTGAAACATA AAATGAATGC

WT-cow      ---------- ---------- ---------- ---------- ----------
Trans-cow   AATTGTTGTT GTTAACTTGT TTATTGCAGC TTATAATGGT TACAAATAAA
pDonor-1    AATTGTTGTT GTTAACTTGT TTATTGCAGC TTATAATGGT TACAAATAAA
pDonor-2    AATTGTTGTT GTTAACTTGT TTATTGCAGC TTATAATGGT TACAAATAAA
```

pEF1α-eGFP-P2A-Puro cassette (3062bp)

```
WT-cow      ---------- ---------- ---------- ---------- ----------
Trans-cow   GCAATAGCAT CACAAATTTC ACAAATAAAG CATTTTTTTC ACTGCATTCT
pDonor-1    GCAATAGCAT CACAAATTTC ACAAATAAAG CATTTTTTTC ACTGCATTCT
pDonor-2    GCAATAGCAT CACAAATTTC ACAAATAAAG CATTTTTTTC ACTGCATTCT

WT-cow      --TATGTGCA GTGTCGACGT GCAGCTCCTT GAATTGCCAC TCACAGGTTC
Trans-cow   AGTTGTGGTT TGTCCAAACT CATCAATGTA TCTTAACGCG TAAATTGTAA
pDonor-1    AGTTGTGGTT TGTCCAAACT CATCAATGTA TCTTAACGCG TAAATTGTAA
pDonor-2    AGTTGTGGTT TGTCCAAACT CATCAATGTA TCTTAACGCG TAAATTGTAA
```

LoxP (34bp) **Cla I**

```
WT-cow      CCAGCACCCA GATCGACGTA CAGGCCTAGG GTTCCAGAAG CTTCCATCAC
Trans-cow   GCGATAACTT CGTATAGCAT ACATTATACG AAGTTATATC GAT-----AC
pDonor-1    GCGATAACTT CGTATAGCAT ACATTATACG AAGTTATATC GAT-----AC
pDonor-2    GCGATAACTT CGTATAGCAT ACATTATACG AAGTTATATC GAT-------
```

Right homology arm (816bp/764bp)

```
WT-cow      ACCCTTTCTA GTGGTCCTCC ACATAGGGCA CCTTTGTCCT GTCTTCTGCA
Trans-cow   ACCCTTTCTA GTGGTCCTCC ACATAGGGCA CCTTTGTCCT GTCTTCTGCA
pDonor-1    ACCCTTTCTA GTGGTCCTCC ACATAGGGCA CCTTTGTCCT GTCTTCTGCA
pDonor-2    ---------- ---------- ---------- ---------- ----------

WT-cow      CATAGATTGG CTCCCTGTCT TTAAACTCCA CGAAAACAGG ATCAGAGAGA
Trans-cow   CATAGATTGG CTCCCTGTCT TTAAACTCCA CGAAAACAGG ATCAGAGAGA
pDonor-1    CATAGATTGG CTCCCTGTCT TTAAACTCCA CGAAAACAGG ATCAGAGAGA
pDonor-2    ----GATTGG CTCCCTGTCT TTAAACTCCA CGAAAACAGG ATCAGAGAGA
```

```
WT-cow       ATCCGTTCTT GTGTGTGAAG TGGGAGCTCA CTGTGGTTTG CATTTGCATT
Trans-cow    ATCCGTTCTT GTGTGTGAAG TGGGAGCTCA CTGTGGTTTG CATTTGCATT
pDonor-1     ATCCGTTCTT GTGTGTGAAG TGGGAGCTCA CTGTGGTTTG CATTTGCATT
pDonor-2     ATCCGTTCTT GTGTGTGAAG TGGGAGCTCA CTGTGGTTTG CATTTGCATT

WT-cow       TCACGGATGA CCCGTGATGT CGAGTCTCTT CTTATCTTTC CTGGCGATGT
Trans-cow    TCACGGATGA CCCGTGATGT CGAGTCTCTT CTTATCTTTC CTGGCGATGT
pDonor-1     TCACGGATGA CCCGTGATGT CGAGTCTCTT CTTATCTTTC CTGGCGATGT
pDonor-2     TCACGGATGA CCCGTGATGT CGAGTCTCTT CTTATCTTTC CTGGCGATGT

WT-cow       GGAGATTTGG GTGGACTTTT GTTTAAAATG CCCCTTCAGG GGTTTCCCTG
Trans-cow    GGAGATTTGG GTGGACTTTT GTTTAAAATG CCCCTTCAGG GGTTTCCCTG
pDonor-1     GGAGATTTGG GTGGACTTTT GTTTAAAATG CCCCTTCAGG GGTTTCCCTG
pDonor-2     GGAGATTTGG GTGGACTTTT GTTTAAAATG CCCCTTCAGG GGTTTCCCTG

WT-cow       GTGGCTGAGT GGTAACGAAA CTGCTTGCCA ATGCAGTAAC ACCAGGAAGA
Trans-cow    GTGGCTGAGT GGTAACGAAA CTGCTTGCCA ATGCAGTAAC ACCAGGAAGA
pDonor-1     GTGGCTGAGT GGTAACGAAA CTGCTTGCCA ATGCAGTAAC ACCAGGAAGA
pDonor-2     GTGGCTGAGT GGTAACGAAA CTGCTTGCCA ATGCAGTAAC ACCAGGAAGA
```

Right homology arm (816bp/764bp)

```
WT-cow       TCCTACATGC CTTGGAGCAG CTAAGCCCAT GCTCCACGAC TACTGAGCCT
Trans-cow    TCCTACATGC CTTGGAGCAG CTAAGCCCAT GCTCCACGAC TACTGAGCCT
pDonor-1     TCCTACATGC CTTGGAGCAG CTAAGCCCAT GCTCCACGAC TACTGAGCCT
pDonor-2     TCCTACATGC CTTGGAGCAG CTAAGCCCAT GCTCCACGAC TACTGAGCCT
```

Surveyor-detect-primer-R

```
WT-cow       GTGCTCTCGG GCCCGGGAGC CACGACGACA GAGCCCTCGA ACCGCAACTG
Trans-cow    GTGCTCTCGG GCCCGGGAGC CACGACGACA GAGCCCTCGA ACCGCAACTG
pDonor-1     GTGCTCTCGG GCCCGGGAGC CACGACGACA GAGCCCTCGA ACCGCAACTG
pDonor-2     GTGCTCTCGG GCCCGGGAGC CACGACGACA GAGCCCTCGA ACCGCAACTG

WT-cow       CTGAGGCCCG TGTGCCTTGG AGCCCGAGCT CCACAGCAAG AGATGCACCA
Trans-cow    CTGAGGCCCG TGTGCCTTGG AGCCCGAGCT CCACAGCAAG AGATGCACCA
pDonor-1     CTGAGGCCCG TGTGCCTTGG AGCCCGAGCT CCACAGCAAG AGATGCACCA
pDonor-2     CTGAGGCCCG TGTGCCTTGG AGCCCGAGCT CCACAGCAAG AGATGCACCA

WT-cow       TAAGGAGAGG CCCGCACTCC ACGACCAGAG TAGCCCCCGC TGGCCACAAC
Trans-cow    TAAGGAGAGG CCCGCACTCC ACGACCAGAG TAGCCCCCGC TGGCCACAAC
pDonor-1     TAAGGAGAGG CCCGCACTCC ACGACCAGAG TAGCCCCCGC TGGCCACAAC
pDonor-2     TAAGGAGAGG CCCGCACTCC ACGACCAGAG TAGCCCCCGC TGGCCACAAC

WT-cow       TAGAGAAAAA CTTGTGCACA GTAGAGAAGA CCCAGGACAG CCAAAAAAAT
Trans-cow    TAGAGAAAAA CTTGTGCACA GTAGAGAAGA CCCAGGACAG CCAAAAAAAT
pDonor-1     TAGAGAAAAA CTTGTGCACA GTAGAGAAGA CCCAGGACAG CCAAAAAAAT
pDonor-2     TAGAGAAAAA CTTGTGCACA GTAGAGAAGA CCCAGGACAG CCAAAAAAAT
```

```
WT-cow      CCAAAGAAAA AGCCCCTTCA GGGCCTTCCT TGGCAGTCCA GTGATCAAGA
Trans-cow   CCAAAGAAAA AGCCCCTTCA GGGCCTTCCT TGGCAGTCCA GTGATCAAGA
pDonor-1    CCAAAGAAAA AGCCCCTTCA GGGCCTTCCT TGGCAGTCCA GTGATCAAGA
pDonor-2    CCAAAGAAAA AGCCCCTTCA GGGCCTTCCT TGGCAGTCCA GTGATCAAGA

WT-cow      CTCTCGCCTC CATTCAAGGG CGCACGGGCT TGATCCCTAG TTGGGGAACT
Trans-cow   CTCTCGCCTC CATTCAAGGG CGCACGGGCT TGATCCCTAG TTGGGGAACT
pDonor-1    CTCTCGCCTC CATTCAAGGG CGCACGGGCT TGATCCCTAG TTGGGGAACT
pDonor-2    CTCTCGCCTC CATTCAAGGG CGCACGGGCT TGATCCCTAG TTGGGGAACT

WT-cow      TAGGATCCCG AAAGTTGTGC AGTACAGCCA GAAATAATAA TAATGATGAA
Trans-cow   TAGGATCCCG AAAGTTGTGC AGTACAGCCA GAAATAATAA TAATGATGAA
pDonor-1    TAGGATCCCG AAAGTTGTGC AGTACAGCCA GAAATAATAA TAATGATGAA
pDonor-2    TAGGATCCCG AAAGTTGTGC AGTACAGCCA GAAATAATAA TAATGATGAA
```

Right homology arm (816bp/764bp)

```
WT-cow      TAAATAAATA AAATGCCCCT CCAGGTCTGT TTCTCATTCC CCTCCCTGCC
Trans-cow   TAAATAAATA AAATGCCCCT CCAGGTCTGT TTCTCATTCC CCTCCCTGCC
pDonor-1    TAAATAAATA AAATGCCCCT CCAGGTCTGT TTCTCATTCC CCTCCCTGCC
pDonor-2    TAAATAAATA AAATGCCCCT CCAGGTCTGT TTCTCATTCC CCTCCCTGCC

WT-cow      TTTCCCCTTG CTTTCTTGCC CAAAGGCCAG CCAGTATTCT CTGGTGGAAG
Trans-cow   TTTCCCCTTG CTTTCTTGCC CAAAGGCCAG CCAGTATTCT CTGGTGGAAG
pDonor-1    TTTCCCCTTG CTTTCTTGCC CAAAGGCCAG CCAGTATTCT CTGGTGGAAG
pDonor-2    TTTCCCCTTG CTTTCTTGCC CAAAGGCCAG CCAGTATTCT CTGGTGGAAG
```

Hind III

```
WT-cow      AACAATCAGC GGGAGGTAAA GAAGATCGAG GCAGTCTCCA CCTCCCACCC
Trans-cow   AACAATCAGC GGGAGGTAAA GAAGATCGAG GCAGTCTCCA CCTCCCACCC
pDonor-1    AACAATCAGC GGGAAAGCTT ---------- ---------- ----------
pDonor-2    AACAATCAGC GGGAGGTAAA GCTT------ ---------- ----------

WT-cow      TGGCCGGACG GGTCACAGGC CTGCAGACCA GGGAGAGGCG CACCACTGCG
Trans-cow   TGGCCGGACG GGTCACAGGC CTGCAGACCA GGGAGAGGCG CACCACTGCG
pDonor-1    ---------- ---------- ---------- ---------- ----------
pDonor-2    ---------- ---------- ---------- ---------- ----------

WT-cow      CACGCGCAGG CGAGGCCCCT GTTGGAGCCC CCGCCCTTGC TGCAGCCCCG
Trans-cow   CACGCGCAGG CGAGGCCCCT GTTGGAGCCC CCGCCCTTGC TGCAGCCCCG
pDonor-1    ---------- ---------- ---------- ---------- ----------
pDonor-2    ---------- ---------- ---------- ---------- ----------

WT-cow      GGACGCGGTG CGGTACGTGG CCAGTCACGC AGCATGCCCA GTCCGAGTGG
Trans-cow   GGACGCGGTG CGGTACGTGG CCAGTCACGC AGCATGCCCA GTCCGAGTGG
pDonor-1    ---------- ---------- ---------- ---------- ----------
pDonor-2    ---------- ---------- ---------- ---------- ----------
```

```
WT-cow        CCAGCCACAG CCACCCTGCA CCCACTCTGG GCAAGGCCCA GCTCTGGCGT
Trans-cow     CCAGCCACAG CCACCCTGCA CCCACTCTGG GCAAGGCCCA GCTCTGGCGT
pDonor-1      ---------- ---------- ---------- ---------- ----------
pDonor-2      ---------- ---------- ---------- ---------- ----------

WT-cow        GTCTGTTGCA CGTCTGTCCC CTCCCACAGC TCTGCGATGT AGCAAAGCGA
Trans-cow     GTCTGTTGCA CGTCTGTCCC CTCCCACAGC TCTGCGATGT AGCAAAGCGA
pDonor-1      ---------- ---------- ---------- ---------- ----------
pDonor-2      ---------- ---------- ---------- ---------- ----------

WT-cow        GGTGGCCAAG GCCAAGGCCA AGGCCAAGGC CACCAACACA CAAGCAGCTC
Trans-cow     GGTGGCCAAG GCCAAGGCCA AGGCCAAGGC CACCAACACA CAAGCAGCTC
pDonor-1      ---------- ---------- ---------- ---------- ----------
pDonor-2      ---------- ---------- ---------- ---------- ----------

WT-cow        AGCTGTGTCC CCGGGAATGG AGGTTGCAGG GCTGGACACA TAACTTCCCT
Trans-cow     AGCTGTGTCC CCGGGAATGG AGGTTGCAGG GCTGGACACA TAACTTCCCT
pDonor-1      ---------- ---------- ---------- ---------- ----------
pDonor-2      ---------- ---------- ---------- ---------- ----------

WT-cow        CCCCCTCCCG CCGTCCCCTG TCCTACGTAT CCAAGAATCA GGATGGCCCA
Trans-cow     CCCCCTCCCG CCGTCCCCTG TCCTACGTAT CCAAGAATCA GGATGGCCCA
pDonor-1      ---------- ---------- ---------- ---------- ----------
pDonor-2      ---------- ---------- ---------- ---------- ----------

                                              3' Junction primer-R
WT-cow        GCCTGGGCCC TGCCCTCCAG GGTCCTCGCA CCCGCAGAGA GGGAGCAATG
Trans-cow     GCCTGGGCCC TGCCCTCCAG GGTCCTCGCA CCCGCAGAGA GGGAGCAATG
pDonor-1      ---------- ---------- ---------- ---------- ----------
pDonor-2      ---------- ---------- ---------- ---------- ----------

WT-cow        CGGGGCCCAC AGGAGGCCCC GGAGGCAGCT GACCGAGGGA CAGATGAGGG
Trans-cow     CGGGGCCCAC AGGAGGCCCC GGAGGCAGCT GACCGAGGGA CAGATGAGGG
pDonor-1      ---------- ---------- ---------- ---------- ----------
pDonor-2      ---------- ---------- ---------- ---------- ----------

WT-cow        ACAAGGCCGT CAGTCCCGCA GGGCTGGGTC CAGCCAGACC CGGGCTGCCA
Trans-cow     ACAAGGCCGT CAGTCCCGCA GGGCTGGGTC CAGCCAGACC CGGGCTGCCA
pDonor-1      ---------- ---------- ---------- ---------- ----------
pDonor-2      ---------- ---------- ---------- ---------- ----------

WT-cow        GGCTGGCGTG TGCGTGCACC TTAAGGGGCA GGCACCGTGT GGCCCACGGT
Trans-cow     GGCTGGCGTG TGCGTGCACC TTAAGGGGCA GGCACCGTGT GGCCCACGGT
pDonor-1      ---------- ---------- ---------- ---------- ----------
pDonor-2      ---------- ---------- ---------- ---------- ----------
```

Query sequence for ZiFiT

Bos Taurus chromosome 25 Btau_4.6.1 Chr25. position 40,631,870- 40,632,430

```
AAGAACCTTGCCCTTGTCCAGGAATCCAGGGGGGCTTCCTGGAAGAGGTGATATGTACACC
AAACCCTAAAGGAGAAGCAGAACTTAAAGAAGCAGAAAGGATGCGTGTGGGGAGCAGAGTC
CATTACAGACCGAGAGAACAGGAAGGGCAGAGGCCCTGAGGCAGATTTGGTGTTCTAAGGA
TTAGAAGGCAGAGTGTCTGGGAACACAGAGTAAGGCTGGAGGGGCAAGGGGGTGAGGTTAG
CCACACTCCCAAACTAGCCGCGGTCTCCACAACTATGTGCAGTGTCGACGTGCAGCTCCTT
GAATTGCCACTCACAGGTTCCCAGCACCCAGATCGACGTACAGGCCTAGGGTTCCAGAAGC
TTCCATCACACCCTTTCTAGTGGTCCTCCACATAGGGCACCTTTGTCCTGTCTTCTGCACA
TAGATTGGCTCCCTGTCTTTAAACTCCACGAAAACAGGATCAGAGAGAATCCGTTCTTGTG
TGTGAAGTGGGAGCTCACTGTGGTTTGCATTTGCATTTCACGGATGACCCGTGATGTCGAG
TCTCTTCTTATC
```
