# Supplementary material for: Single Cas9 nickase induced generation of NRAMP1 knockin cattle with reduced off-target effects
Source: Genome Biol. 2017 Feb 1;18:13. doi: 10.1186/s13059-016-1144-4 (PMC5286826; doi:10.1186/s13059-016-1144-4)
Supplement: Additional file 3: — Supplemental Methods, Figures, and Tables. Supplemental Methods, Figures S1–S9, and Tables S1–S7. (PDF 1638 kb) [file 13059_2016_1144_MOESM3_ESM.pdf]

Supplementary Methods

Karyotype analysis

Chromosomal number analysis was carried out as previously reported [1]. In brief, cells were treated with colchicine for 6 h, followed by 40 mM KCl for 20 min at 37℃. Cells were then fixed with ice-cold acetic acid/methanol (1:3, vol/vol), and stained with Giemsa. Of the 100 cells examined, more than 95% cells had the normal karyotype (2n=60, XX) and were considered suitable for SCNT.

Real-time RT-PCR

Total RNA was isolated from tissues (tissue samples ground into a fine powder in liquid nitrogen) or macrophages using Trizol reagent (Invitrogen, CA, USA). Purified RNA was reverse-transcribed using a SYBR PrimeScript RT-PCR Kit (TaKaRa, Tokyo, Japan). Real-time RT-PCR was performed with an ABI StepOnePlus real-time PCR system (Applied Biosystems, CA, USA) using SYBR Premix ExTaq II (TaKaRa) as previously described [2]. The comparative C_t method was used to calculate the relative quantity of the target gene mRNA, normalized to bovine glyceraldehyde 3-phosphate dehydrogenase (GAPDH), and was expressed as the fold change = $2^{-\Delta\Delta Ct}$. Primer sequences used for qPCR are listed in Table S7.

Western blot analysis

Cells or liquid nitrogen grinded tissues were lysed in ice-cold RIPA cell buffer supplemented with protease inhibitors (Thermo Scientific, NH, USA). The proteins were separated with 12% acrylamide gels and transferred to PVDF membranes (Millipore, MA, USA) [3]. Probing was performed with specific primary antibodies and HRP-conjugated secondary antibodies (Beyotime technology, Jiangsu, China). The primary antibodies used were GAPDH (ab187172, 1:1000; Abcam, Cambridge, MA) and Nramp1 (ab59696, 1:300; Abcam).

Isolation and differentiation of peripheral blood mononuclear cells (PBMCs)

PBMCs were isolated from blood of control or transgenic cattle using Histopaque-1077 (Sigma-Aldrich, MO, USA) according to the manufacturer's instructions. Macrophages used in this study were derived from PBMCs by stimulation with granulocyte-macrophage colony-stimulating factor (GM-CSF) for 7 days [4]. Macrophages express high levels of CD14 and CD11b, while monocytes express high CD14 and very low CD11b; therefore, CD14 and CD11b surface markers were used to differentiate between monocytes and monocyte-derived macrophages (MDM). The efficiency of differentiation was assessed using Western blotting of CD11b and CD14

surface antigen expression. The percentage of macrophages (SI Appendix, Fig. S8) above 80% was considered to be suitable for the following experiments. (MDM) were cultured in RPMI-1640 medium (Gibco, NY, USA) containing 10% (vol/vol) FBS, 20 M HEPES, and 2 mM glutamine.

Flow cytometry analysis

Annexin V staining was performed using an Annexin V-FITC/PI Apoptosis Detection Kit (Molecular Probes, Invitrogen) according to the manufacturer's instructions. Cells were analyzed using a BD LSR II flow cytometer (BD Biosciences, CA, USA) as previously described to differentiate between necrosis and apoptosis. Data from three independent experiments were analyzed with FlowJo data analysis software (FlowJo, LLC, OR, USA).

References

1. He YL, Wu YH, He XN, Liu FJ, He XY, Zhang Y: **An immortalized goat mammary epithelial cell line induced with human telomerase reverse transcriptase (hTERT) gene transfer.** *Theriogenology* 2009, **71:**1417-1424.
2. Wu HB, Wu YY, Ai ZY, Yang LP, Gao Y, Du J, Guo ZK, Zhang Y: **Vitamin C Enhances Nanog Expression Via Activation of the JAK/STAT Signaling Pathway.** *Stem Cells* 2014, **32:**166-176.
3. Liu X, Wang YS, Guo WJ, Chang BH, Liu J, Guo ZK, Quan FS, Zhang Y: **Zinc-finger nickase-mediated insertion of the lysostaphin gene into the beta-casein locus in cloned cows.** *Nature Communications* 2013, **4**.
4. Wu HB, Wang YS, Zhang Y, Yang MQ, Lv JX, Liu J, Zhang Y: **TALE nickase-mediated SP110 knockin endows cattle with increased resistance to tuberculosis.** *Proceedings of the National Academy of Sciences of the United States of America* 2015, **112:**E1530-E1539.

Supplementary figure legends

Fig. S1 Selection of sgRNAs.

Fig. S2 Representative sequences from Sanger sequencing. Some of the representative sequences revealed distinct Cas9-induced insertions and deletions at target site 22 (up) and 45 (down). The PAM sequences were underlined and highlighted in red; the targeting sequences were underlined in red; the lowercase letters represent inserted bases. Occurrences of deletions and insertions are listed on the right, deletions (-), and insertions (+).

Fig. S3 Off target sites of sgRNA 2 and 22 with top 15 ChIP-seq binding density. All the off-target sites were computationally identified with 20-bp long sequence; these sites ended with PAM and aligned most effectively with the sgRNA guiding sequence in each peak. OT means off-target, and all the off-target sites in the same group were ranked according to ChIP-seq binding density (peak fold enrichment), as shown on the right-hand bar graphs. At off-target sites, bases matching the sgRNA guiding sequence and PAM sequence are highlighted in green and red, respectively.

Fig. S4 Percent preservation analyses. Percent preservation of bases at the main off-target sites compared with the guiding sequence of sgRNA2 (above) and sgRNA 22 (below).

Fig. S5 Overexpression of bovine *NRAMP 1* in Raw 264.7. Bacterial loads of Raw 264.7 macrophage-like cell lines infected with *M. bovis* were determined by CFU assays. Data are presented as the mean ± SD and derived from at least three independent experiments.

Fig. S6 Representative junction PCR results of G418-resistant colonies. Representative 5' junction (up), 3' junction (down) PCR results of puromycin-resistant colonies. Red fonts represent positive results.

Fig. S7 A typical and representative karyotype of gene-targeted colonies

Fig. S8 Sanger sequencing results of transgenic cattle

Fig. S9 Identification of monocyte-derived macrophages. Macrophages were derived from monocytes by stimulation with GM-CSF. Macrophages express high levels of CD14 and CD11b, whereas monocytes express high levels of CD14 and very low levels of CD11b. GAPDH serves as a loading control.

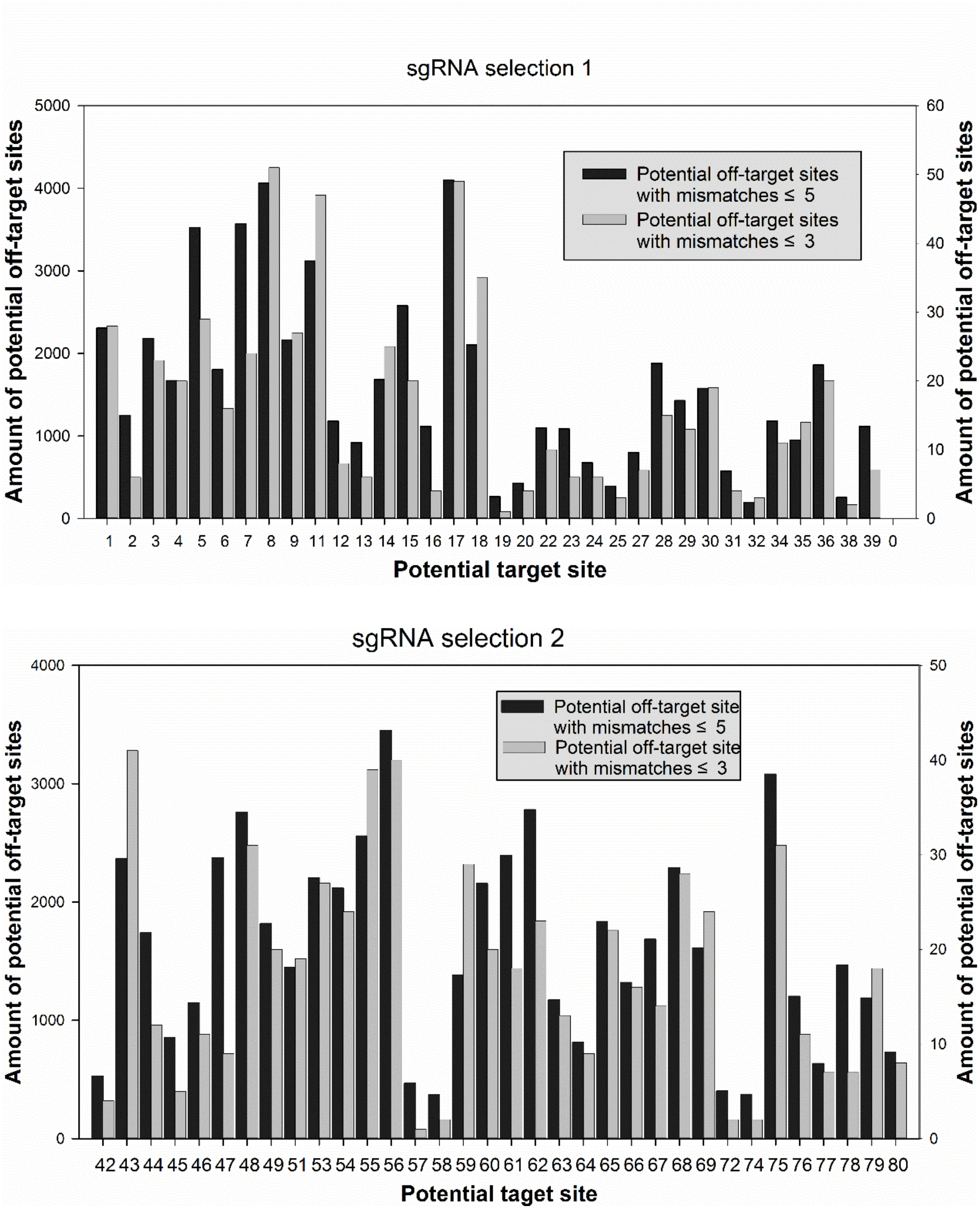
sgRNA selection 1
Potential off-target sites with mismatches ≤ 5
Potential off-target sites with mismatches ≤ 3
Amount of potential off-target sites
Potential target site
sgRNA selection 2
Potential off-target site with mismatches ≤ 5
Potential off-target site with mismatches ≤ 3
Amount of potential off-target sites
Potential taget site

Fig. S1

```
TCACACCCTTTCTAGTGGTCCTCCACATAGGGCACCTTTGTCCTGTCTTCTGCACATAG    (WT)
TCACACCCTTTCTAGTGGTCCTCCACATAGGGCACCTTTGTCCTGTCTTCTGCACATAG    (+1)
                             a
TCACACCCTTTCTAGTGGTCCTCCACATAGGGCACCTTTGTCCTGTCTTCTGCACATAG    (+4)
                           caga
TCACACCCTTT---------------------CACCTTTGTCCTGTCTTCTGCACATAG    (-21)
TCACACCCTTTCTAGTGGTCCTCCACAT---GCACCTTTGTCCTGTCTTCTGCACATAG    (-3)
TCACACCCTTTCTAGTGGTCCTCCACATA---c-----TGTCCTGTCTTCTGCACATAG    (-9, +1)
TCACACCCTTTCTAGTGGTCCT---------------TTGTCCTGTCTTCTGCACATAG    (-15)
```

WT Cas9-sgRNA 22
Indels: 21.43% (39/182)
Insertions: 20.51% (8/39)
Deletions: 87.17% (34/39)

```
CACCCTTTCTAGTGGTCCTCCACATAGGGCACCTTTGTCCTGTCTTCTGCACATAGATT    (WT)
CACCCTTTCTAGTGGTCCTCCACATAGGGCACCTTTGTCCTGTCTTCTGCACATAGATT    (+2)
                        ac
CACCCTTTCTAGTGGTCCTCCA-ATAGGGCACCTTTGTCCTGTCTTCTGCACATAGATT    (-1)
CACCCTTTCTAGTGGTCCTCC-------------TTGTCCTGTCTTCTGCACATAGATT    (-13)
CACCCTTTCTAGTGGTC------ATAGGGCACCTTTGTCCTGTCTTCTGCACATAGATT    (-6)
CACCCTTTCTAGTGGTCCTCCA--TAGGGCACCTTTGTCCTGTCTTCTGCACATAGATT    (-2)
CACCCTTTCTAGT----ct----------------------GTCTTCTGCACATAGATT    (-28, +2)
```

WT Cas9-sgRNA 45
Indels: 41.90% (75/179)
Insertions: 18.67% (14/75)
Deletions: 90.67% (68/75)

Fig. S2

Binding sites **sgRNA guiding sequence and base position**

sgRNA-2	Location	1	2	3	4	5	6	7	8	9	10	11	12	13	14	15	16	17	18	19	20	P	A	M
ON	chr25_40631966_40632466	A	A	C	C	T	G	T	G	A	G	T	G	G	C	A	A	T	T	C	A	A	G	G
OT-1	chr4_1192145_1192645	A	G	C	C	T	G	T	G	A	G	C	T	A	C	A	C	T	A	C	A	T	G	G
OT-2	chr3_103129008_103129508	A	A	A	C	T	T	T	G	A	T	T	G	T	C	A	A	A	T	T	A	A	G	G
OT-3	chr4_25172348_25172848	A	A	C	A	A	A	T	G	A	G	T	G	T	C	A	A	T	T	A	G	A	G	G
OT-4	chr24_17776379_17776879	A	C	C	T	G	G	T	G	T	G	C	T	G	C	A	A	T	T	C	A	T	G	G
OT-5	chr24_45736136_45736636	A	A	C	T	T	G	T	G	A	A	T	A	C	A	A	A	T	A	C	A	A	G	G
OT-6	chr26_33309894_33310394	A	T	C	A	T	G	T	G	A	G	T	G	A	A	A	G	T	T	C	T	T	G	G
OT-7	chr5_111340663_111341163	A	A	C	C	T	G	T	G	G	G	C	T	A	C	A	G	T	C	C	A	T	G	G
OT-8	chr10_45996655_45997155	A	G	C	C	T	T	T	G	A	G	G	T	G	C	A	A	T	T	T	G	G	G	G
OT-9	chr9_3820599_3821099	A	G	C	C	T	G	T	G	T	G	C	T	G	C	A	G	T	C	C	A	T	G	G
OT-10	chr9_99399395_99399895	A	A	C	C	T	T	T	G	A	G	G	G	A	A	A	C	T	G	C	A	A	G	G
OT-11	chr3_124395553_124396053	G	A	C	C	T	G	A	G	T	A	A	G	G	C	C	A	T	T	C	A	T	G	G
OT-12	chr8_48138706_48139206	A	G	C	A	T	T	T	A	A	G	T	T	G	C	T	A	T	T	C	A	G	G	G
OT-13	chr1_108472728_108473228	A	A	T	A	T	C	T	G	G	G	T	G	C	C	A	G	T	T	C	A	T	G	G
OT-14	chr27_38949057_38949557	A	A	G	C	A	C	T	G	T	G	T	G	G	C	A	A	T	T	A	T	G	G	G
OT-15	chr18_41337881_41338381	A	T	G	A	T	A	T	G	A	G	T	G	G	G	A	A	T	A	C	A	A	G	G

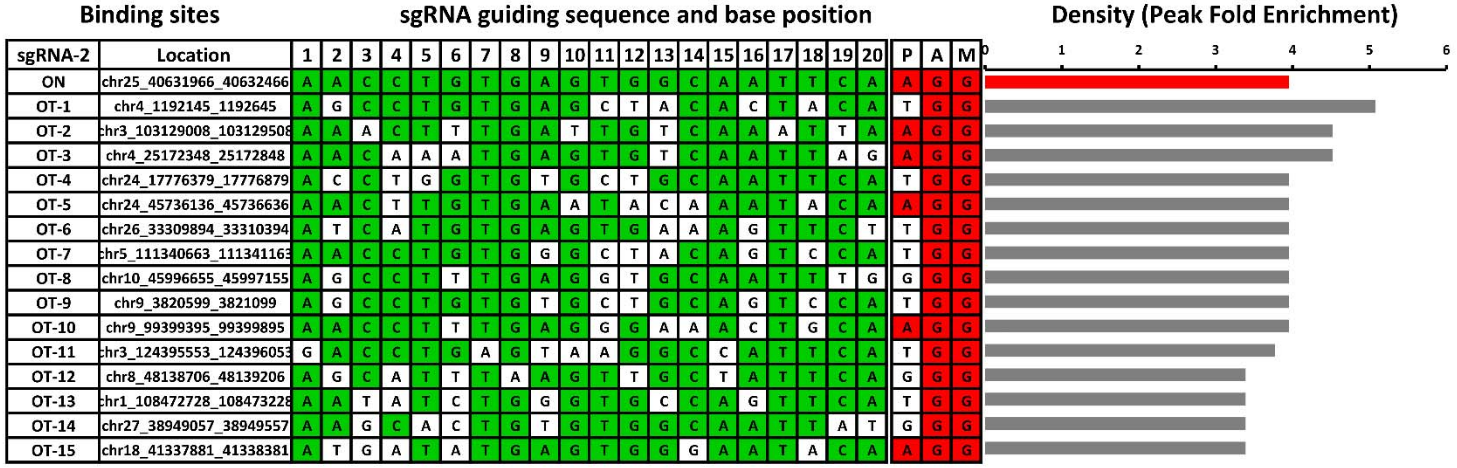

Binding sites **sgRNA guiding sequence and base position**

sgRNA-22	Location	1	2	3	4	5	6	7	8	9	10	11	12	13	14	15	16	17	18	19	20	P	A	M
ON	chr25_40631867_40632367	C	C	T	G	T	C	T	T	C	T	G	C	A	C	A	T	A	G	A	T	T	G	G
OT-1	chr3_35217867_35218367	C	C	T	C	T	C	A	T	A	T	T	C	G	C	A	T	G	G	A	T	A	G	G
OT-2	chr4_68632846_68633346	C	T	T	T	T	C	T	T	C	T	G	C	A	C	T	C	A	G	A	T	G	G	G
OT-3	chr6_115453345_115453845	C	C	T	T	A	T	T	T	T	T	T	C	A	C	A	G	A	G	A	T	C	G	G
OT-4	chr17_41912018_41912518	C	C	T	G	A	C	A	G	C	T	G	C	A	G	A	G	A	A	A	T	A	G	G
OT-5	chr25_34473846_34474346	C	C	C	G	T	C	C	T	C	T	G	G	A	C	A	G	A	G	C	T	T	G	G
OT-6	chr13_17174650_17175150	C	C	T	G	T	C	T	A	G	T	G	A	A	A	A	G	A	G	G	T	G	G	G
OT-7	chr2_132559396_132559896	C	C	A	C	C	C	T	T	C	T	G	C	C	C	A	G	A	G	C	T	G	G	G
OT-8	chr19_52727848_52728348	A	C	A	G	T	C	C	T	C	T	G	G	A	C	A	T	A	A	T	T	T	G	G
OT-9	chr14_68032489_68032989	T	C	T	G	T	C	T	T	G	A	G	C	A	T	A	T	A	T	C	T	A	G	G
OT-10	chr9_80334366_80334866	C	T	T	G	T	T	T	G	C	T	G	G	T	C	T	T	A	G	A	T	A	G	G
OT-11	chr4_55591291_55591791	C	T	T	G	T	C	T	T	G	T	C	C	A	C	T	T	A	G	A	A	G	G	G
OT-12	chr12_84971585_84972085	C	C	T	G	C	A	G	T	T	T	T	C	A	C	A	T	A	G	C	T	T	G	G
OT-13	chr5_121981533_121982033	C	C	C	T	T	C	T	T	C	T	G	A	T	A	A	A	A	G	A	T	G	G	G
OT-14	chr25_38144453_38144953	A	G	T	G	G	T	T	T	C	T	G	A	A	C	A	T	A	G	A	T	A	G	G
OT-15	chr23_18173144_18173644	G	C	T	G	T	C	T	T	G	G	G	T	G	C	A	T	A	C	A	T	G	G	G

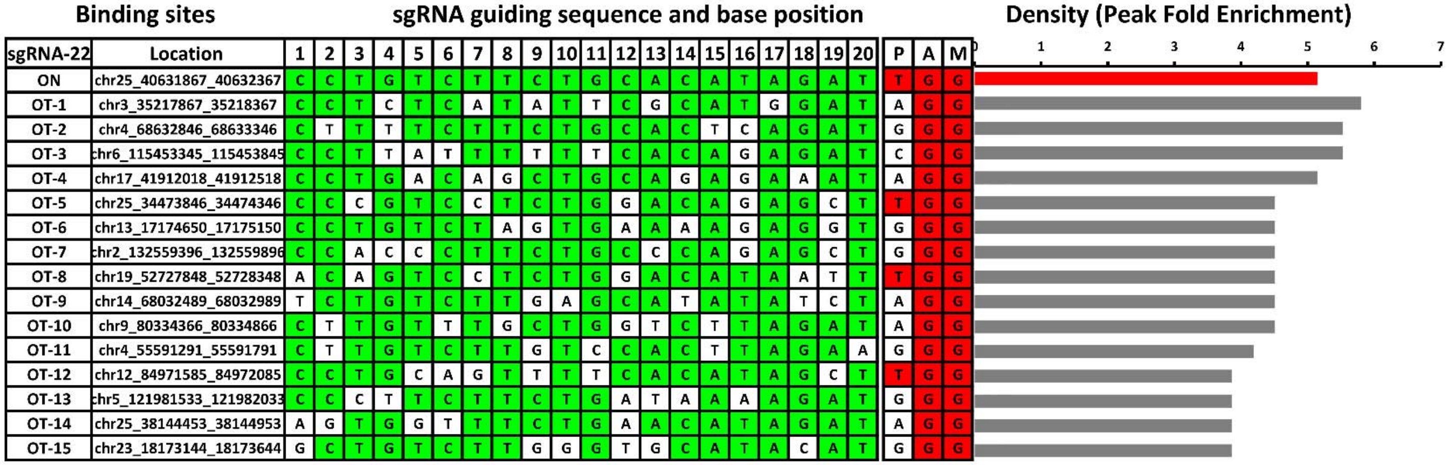

Fig. S3

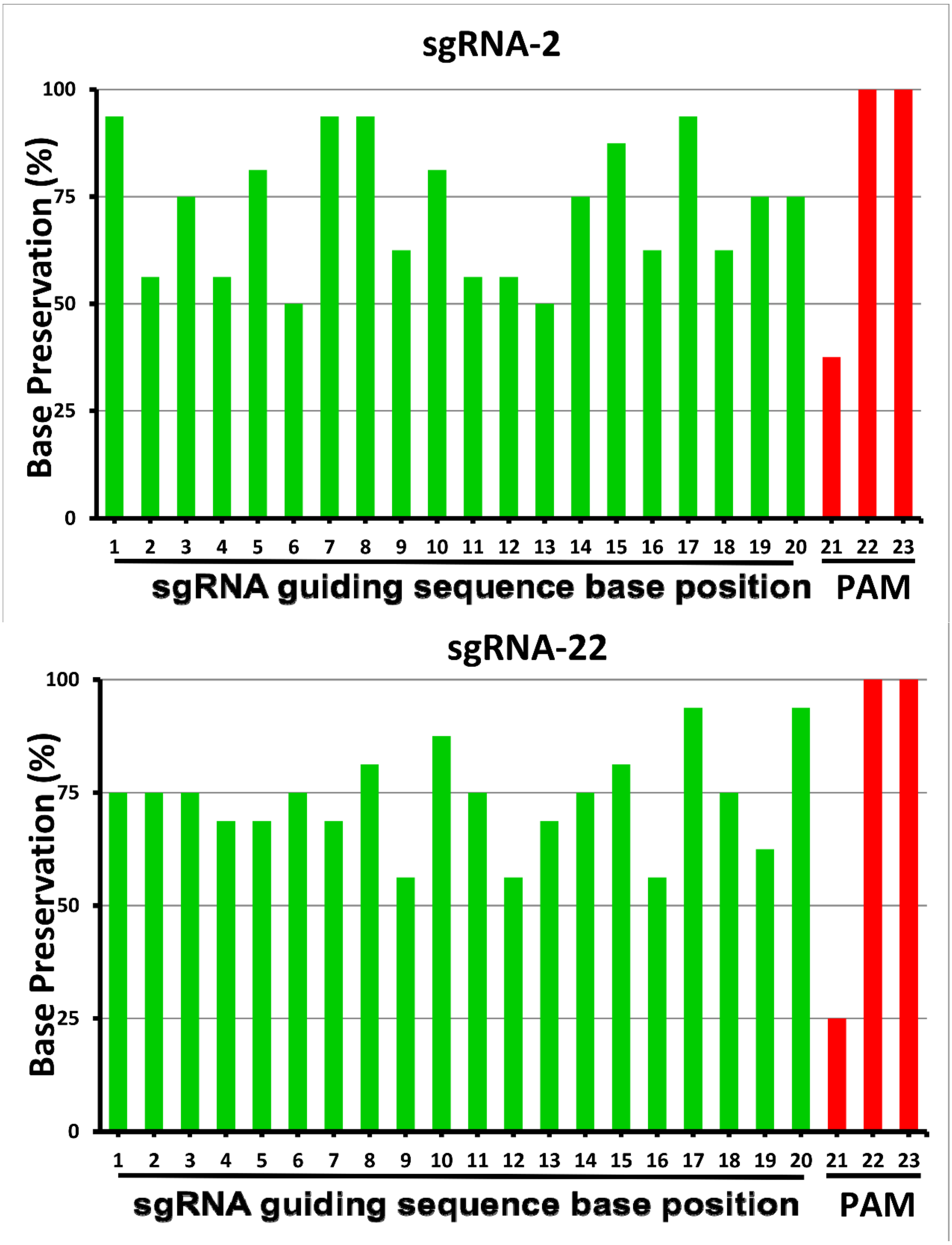
sgRNA-2
Base Preservation (%)
100
75
50
25
0
1 2 3 4 5 6 7 8 9 10 11 12 13 14 15 16 17 18 19 20 21 22 23
sgRNA guiding sequence base position
PAM
sgRNA-22
Base Preservation (%)
100
75
50
25
0
1 2 3 4 5 6 7 8 9 10 11 12 13 14 15 16 17 18 19 20 21 22 23
sgRNA guiding sequence base position
PAM

Fig. S4

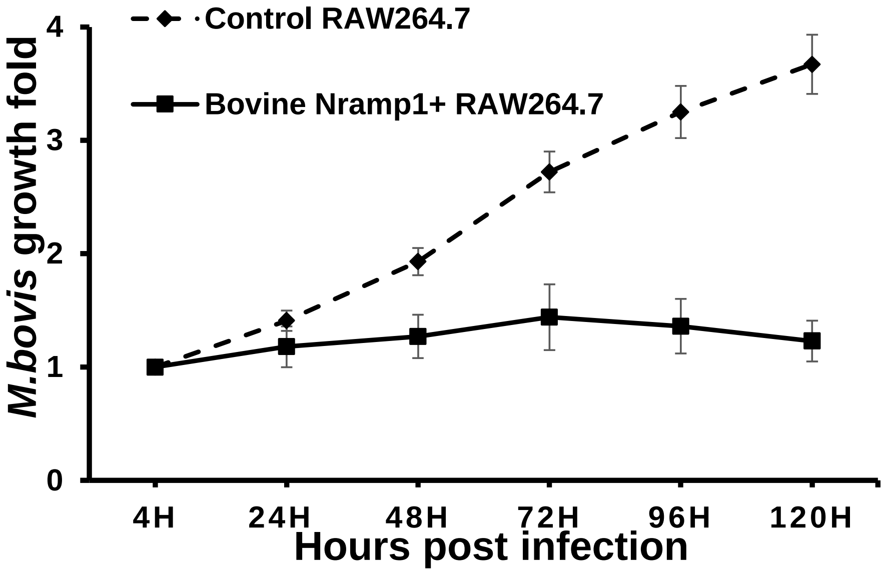

Control RAW264.7
Bovine Nramp1+ RAW264.7
M.bovis growth fold
0
1
2
3
4
4H
24H
48H
72H
96H
120H
Hours post infection

Fig. S5

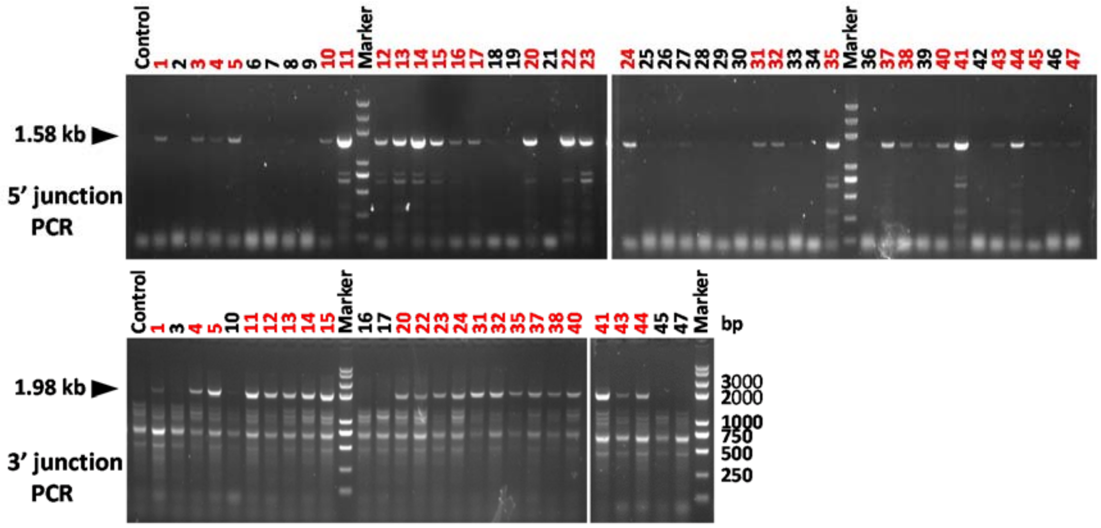
Control
1
2
3
4
5
6
7
8
9
10
11
Marker
12
13
14
15
16
17
18
19
20
21
22
23
24
25
26
27
28
29
30
31
32
33
34
35
Marker
36
37
38
39
40
41
42
43
44
45
46
47
1.58 kb
5' junction PCR
Control
1
3
4
5
10
11
12
13
14
15
Marker
16
17
20
22
23
24
31
32
35
37
38
40
41
43
44
45
47
Marker
bp
1.98 kb
3' junction PCR
3000
2000
1000
750
500
250

Fig. S6

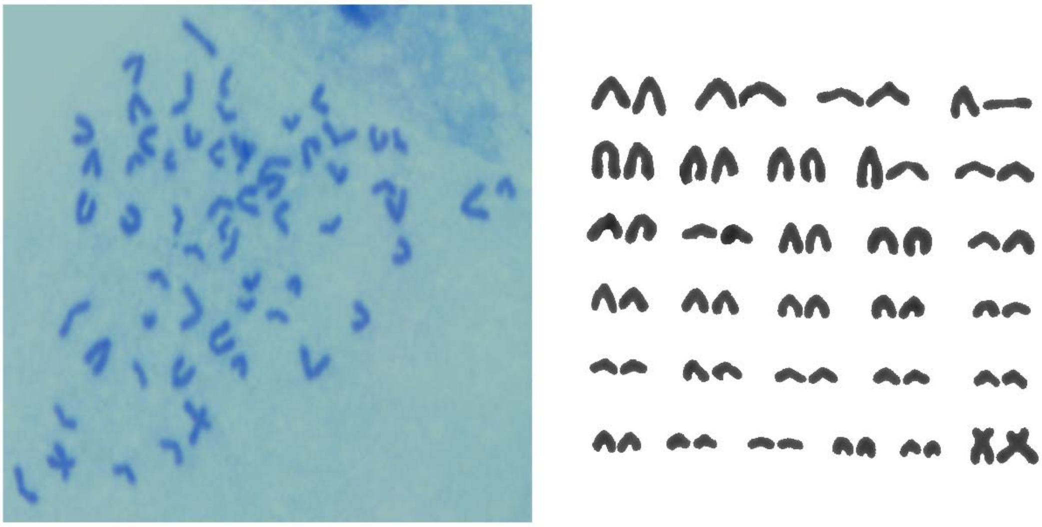

Fig. S7

Sanger sequence of sgRNA45 off target site 1

Control	TACCCTTCCGCCGCCTAGCAAAGAGTCCCTACGTGGAGGATGTGCTTACCTAAGCTATTA
Cas9n #1-9	TACCCTTCCGCCGCCTAGCAAAGAGTCCCTACGTGGAGGATGTGCTTACCTAAGCTATTA
WT Cas9 #1	TACCCTTCCGCCGCCTAGCAAAGAGTCCCT--GTGGAGGATGTGCTTACCTAAGCTATTA
WT Cas9 #2	TACCCTTCCGCCGCCTAGCAAAGAGTCCCTACGTGGAGGATGTGCTTACCTAAGCTATTA

Sanger sequence of sgRNA45 off target site 3

Control	GCACTGCTCACAATACCACACAGGGCCCCTATATGAAGGCCATGGCAGAAGCTCCTCTCC
Cas9n #1-9	GCACTGCTCACAATACCACACAGGGCCCCTATATGAAGGCCATGGCAGAAGCTCCTCTCC
WT Cas9 #1	GCACTGCTCACAATACCACACAGGGCCCCTATATGAAGGCCATGGCAGAAGCTCCTCTCC
WT Cas9 #2	GCACTGCTCACAATACCACACAGGGCCC-T-CATGAAGGCCATGGCAGAAGCTCCTCTCC

Sanger sequence of sgRNA45 off target site 10

Control	AAAGGGTACTCTGCCTGGATGATTTGCCCTATGTGGGGGCCCAAGAGGAGGCAGGGGGCC
Cas9n #1-9	AAAGGGTACTCTGCCTGGATGATTTGCCCTATGTGGGGGCCCAAGAGGAGGCAGGGGGCC
WT Cas9 #1	AAAGGGTACTCTGCCTGGATGAT------TATGTGGGGGCCCAAGAGGAGGCAGGGGGCC
WT Cas9 #2	AAAGGGTACTCTGCCTGGATGATTT--CCTATGTGGGGGCCCAAGAGGAGGCAGGGGGCC

Fig. S8

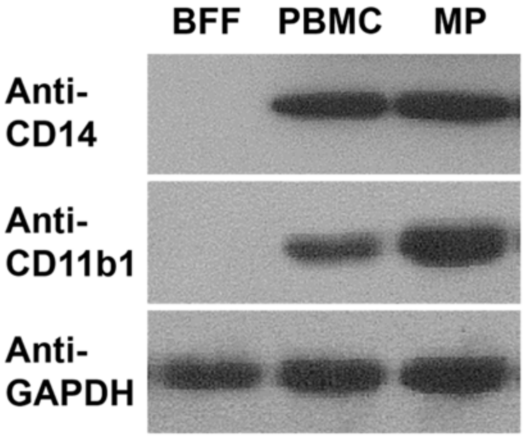

Fig. S9

Supplementary Tables

Table. S1 Primer sequences for sgRNA cloning

Number	Top Guide oligo	Bottom Guide oligo
**2**	CACCGAACCTGTGAGTGGCAATTCA	AAACTGAATTGCCACTCACAGGTTC
**16**	CACCGATGTGGAGGACCACTAGAAA	AAACTTTCTAGTGGTCCTCCACATC
**19**	CACCGCAGATCGACGTACAGGCCTA	AAACTAGGCCTGTACGTCGATCTGC
**20**	CACCGCAGCACCCAGATCGACGTAC	AAACGTACGTCGATCTGGGTGCTGC
**22**	CACCGCAGGACAAAGGTGCCCTATG	AAACCATAGGGCACCTTTGTCCTGC
**24**	CACCGCCACACTCCCAAACTAGCCG	AAACCGGCTAGTTTGGGAGTGTGGC
**32**	CACCGCCTAGGCCTGTACGTCGATC	AAACGATCGACGTACAGGCCTAGGC
**34**	CACCGCCTGTCTTCTGCACATAGAT	AAACATCTATGTGCAGAAGACAGGC
**38**	CACCGCTAGGCCTGTACGTCGATCT	AAACAGATCGACGTACAGGCCTAGC
**42**	CACCGCTGTACGTCGATCTGGGTGC	AAACGCACCCAGATCGACGTACAGC
**45**	CACCGGACAAAGGTGCCCTATGTGG	AAACCCACATAGGGCACCTTTGTCC
**58**	CACCGGTGGAGACCGCGGCTAGTTT	AAACAAACTAGCCGCGGTCTCCACC
**72**	CACCGTGTACGTCGATCTGGGTGCT	AAACAGCACCCAGATCGACGTACAC
**74**	CACCGTGTGGAGACCGCGGCTAGTT	AAACAACTAGCCGCGGTCTCCACAC

Table. S2 Cleavage fragments size distribution and genome modification rates estimate

Sequence Number	On-target site	Fragment Top (bp)	Fragment Bottom (bp)	f_{cut} (%)	Indels (%)
**2**	AACCTGTGAGTGGCAATTCA	562	475	4.28	2.16
**16**	ATGTGGAGGACCACTAGAAA	637	400	14.95	6.48
**19**	CAGATCGACGTACAGGCCTA	611	426	42.16	23.95
**20**	CAGCACCCAGATCGACGTAC	604	433	54.87	32.82
**22**	CAGGACAAAGGTGCCCTATG	654	383	40.77	23.04
**24**	CCACACTCCCAAACTAGCCG	522	515	35.59	19.75
**32**	CCTAGGCCTGTACGTCGATC	593	444	15.37	8.01
**34**	CCTGTCTTCTGCACATAGAT	690	347	25.80	13.86
**38**	CTAGGCCTGTACGTCGATCT	592	445	30.91	16.89
**42**	CTGTACGTCGATCTGGGTGC	586	451	33.22	18.28
**45**	GACAAAGGTGCCCTATGTGG	651	386	72.76	47.81
**58**	GTGGAGACCGCGGCTAGTTT	512	525	13.97	7.25
**72**	TGTACGTCGATCTGGGTGCT	585	452	31.96	17.51
**74**	TGTGGAGACCGCGGCTAGTT	513	524	12.53	7.78

Table. S3 Primer sequences for amplification of each on/off target region.

Name	Primer Forward	Primer Reverse	Length (bp)	Tm (℃)
sgRNA45-ON	AACAGGAAGGGCAGAGGC	CGGGTCATCCGTGAAATG	401	55
sgRNA45-OT1	TGCCTCCCTCCGCACGAT	AGCCGTCCCCTTCCACCTC	313	57
sgRNA45-OT2	GCATCTTTCAGCCCTTCA	TCCACCAGCAAACCCTCT	353	54
sgRNA45-OT3	AGGAAATCAGAGCCAAGA	GAACCAGGTCCAAGTGAG	320	50
sgRNA45-OT4	TATTATGATGTCCCAATC	CATTTCAAGCATATTTACAC	427	45
sgRNA45-OT5	CCCCAAAGTGCAAGGATA	GGAACTAAACCCACAAGA	414	50
sgRNA45-OT6	GTCGGACATGACTGAAGC	GGGAGAAGAAACCCAATT	467	52
sgRNA45-OT7	ATGGGTATAGCAGGTTCT	CTGTATCTTGGGCACTCT	373	49
sgRNA45-OT8	AGGGCCATTTCTTACATT	TCTTTCCTGGAAGTTGCT	335	48
sgRNA45-OT9	TGTAAGAGGCTGCTAAGTT	ACCACATGGTCTCCCAAA	429	50
sgRNA45-OT10	GAGTCACTTATGGAGCAAA	GCTTCTTCAAGGGATAGA	338	50
sgRNA45-OT11	CACTTCTTCCTTGGCTCT	GTCTGGGGCTAAATCTGA	323	49
sgRNA45-OT12	CCAAAAGTCTTCGAGTGC	GGGGAAAGGGATAAATAA	307	49
sgRNA45-OT13	GGAGACACGGTTTTAGAT	AAGATTCCCCAAGATACA	432	50
sgRNA45-OT14	GTGATGGGAGAAGAGGAG	GATGCGTGATAACTGAAAT	405	50
sgRNA45-OT15	ATAATCTGGGTGAGGGAA	ATGGCAGAGGATTTAGTG	363	46
sgRNA20-ON	AACAGGAAGGGCAGAGGC	CGGGTCATCCGTGAAATG	401	55
sgRNA20-OT1	GGCTCAGAGCTTACCTCGTCC	TTGGAACTGCTGCCGTGAA	397	61
sgRNA20-OT2	TGTTAGCCCTCCAGACCG	GACGCATAGCCTCCCTCA	308	52
sgRNA20-OT3	ATATCACTGACTGCACAT	ATAAATACTAGCCTCCAC	376	46
sgRNA20-OT4	CCCCTCAAGTAGTAGGTT	AGTTTGGGTAGACTCTGG	310	50
sgRNA20-OT5	CTTGTGCCAACTGAACTT	TGGAGCCAGATACACCTT	399	50
sgRNA20-OT6	CAAACAAGGGAGAATGGG	TGTGACGGCAGATACTGG	331	52
sgRNA20-OT7	TTTCACCAGCAAGCATTT	GTCATTGTGGAGGTCAGC	317	52
sgRNA20-OT8	GGCTGCGTAATCTTGGTG	ACTGCTGCTGGTGGTAAT	324	54
sgRNA20-OT9	TAGAGGAGGAAACTAAGAGG	CAAGACCAGATGGGTAAA	392	51
sgRNA20-OT10	CTGGTTCCCGGCAATCTC	GCAGCCCACAGTCACAAT	426	49
sgRNA20-OT11	GACAGAGGAGCCTAGTGG	TAATCAAGTTTGGCAGGA	400	48
sgRNA20-OT12	GGGCAAGCCTGTCTGAAC	GGGTGCCTATCACCATCAG	452	55
sgRNA20-OT13	CCTGAAGTCTGTAGGGAAAT	CTAGGACTGGCGAGGAAG	390	53
sgRNA20-OT14	GCTCCTTAGCAGCTTTCT	GTTTGTTGCAGTATTTCG	358	50
sgRNA20-OT15	CATCCTCCTCCCATTACC	TGTGAGCATTTCCCTCTAT	317	52
sgRNA22-ON	AACAGGAAGGGCAGAGGC	CGGGTCATCCGTGAAATG	401	55
sgRNA22-OT1	GGGAGAACTGTAAGCTATTA	CACCCAGAGTCAGAAGAA	360	50
sgRNA22OT2	AGGCACCACCTCTTCTAC	TACGAGCAGTTTGCTTATT	437	50
sgRNA22-OT3	TGGTCATTTAACGCAACA	ATCAGACTCTGGGCAAGT	324	51
sgRNA22-OT4	TCCAGGAGATAATGAAGG	GTGTCTGTTGCTGGCTTT	375	50
sgRNA22-OT5	GACTCAGCAGAGCCAAAT	TTGAAAGAGCCTCAGGAC	367	50
sgRNA22-OT6	GAGGTAAGCTGCTGGAGG	GAAAGTCGCTCAGTCGTG	411	54
sgRNA22-OT7	TTGGTGGCAGCCTTTATT	GCAGCCTACAGTCTTGGATT	321	54

Name	Primer Forward	Primer Reverse	Length (bp)	Tm ℃
sgRNA22-OT8	ATCCACCCAATCAGAACTCC	ACACCCTTAGCGGCAGTC	460	56
sgRNA22-OT9	ATGGAATCATGTAATGTG	GATGTAAAGAAACTGGAA	411	45
sgRNA22-OT10	TCTCAGTTAAGAGGTAGCAAGT	TTGGTCCTTAGATTCACATT	303	48
sgRNA22-OT11	CATTGGGATGACAGATAC	GGGTCCGTGATGAGTTTC	345	48
sgRNA22-OT12	CCCGAGTGTCACAATCCA	ATATCCTGTCTGTGCCTGA	456	54
sgRNA22-OT13	TTAGAGCCTGTATTTGTTTC	GGTCTCATTTACGCCATT	348	49
sgRNA22-OT14	CCCCTGCATTGGAAACTC	GCACAGCAACGAAGACCC	360	50
sgRNA22-OT15	TTCCGCTGGCTCTTTGAC	ATCCGACTACAGACCTTTCC	408	55
sgRNA2-ON	AACAGGAAGGGCAGAGGC	CGGGTCATCCGTGAAATG	401	55
sgRNA2-OT1	TTGATTTCCCAACTTCTC	TTGTTGTTCAGTCGCTAA	317	48
sgRNA2-OT2	TTCTACCAGCATTTCCAT	AGATAATCTTGCCTACCC	375	48
sgRNA2-OT3	GTGAAGTCGCTCAGTCGT	GGCAGCCCTAGCAAAGTA	492	49
sgRNA2-OT4	TTTGTAGCAATCCCAAGA	GTTCAGTTCAGTCGCTCA	366	50
sgRNA2-OT5	ACATTAGGGTTCTCGGCAGTC	GAGCCTCAGTGTCCTGTTTCAT	478	53
sgRNA2-OT6	AGAATGGGTGTTCTATCAA	CTCGTAGAGCAGCTTACC	301	51
sgRNA2-OT7	AGTCAGGACCCCAACAAG	TGGCACCAGAAGATAGCA	389	51
sgRNA2-OT8	TCAGAAGTTAAGCCCAGGTT	CAGGCACGCAATTCATAT	393	53
sgRNA2-OT9	TCAGCATAGCCCAAGAAG	CTGTTGTATAGCCAATAACACT	333	50
sgRNA2-OT10	GATTGAAGCAGCAAGTGA	ATAAAGCATCCCCAGAAA	399	46
sgRNA2-OT11	GTTCACCACCCCTTTGTT	AGGAATTTGCATCTTTGCT	383	55
sgRNA2-OT12	CTCCACTCGGAAGTTCAA	AGCCCACAGAGCACAAAA	305	48
sgRNA2-OT13	CAGCAAGGTCAAAAGAAA	CAGTAGATGCCCCTACAT	439	47
sgRNA2-OT14	TCCTCCTGTTGCTGAATC	CTAAGTGCCACGTTTGCT	306	52
sgRNA2-OT15	GCGTCACAAAGAGTCAGA	GGGCTCCAGTTCAGCATAT	465	48

Table. S4 WT Cas9 and single Cas9 mediated indel analysis at predicted dCas9 binding locations. The sequence located ± 100 bp on both sides of binding site within ChIP-Seq were determined below. The group of WT Cas9 without sgRNA was taken as control. We performed Fisher exact statistical taste between WT Cas9 with sgRNA and control. Sites with significant indels were highlighted yellow (P value < 0.05). The Fisher exact taste between single Cas9 with sgRNA and control was shown in the last row.

			WT Cas9 with sgRNA			WT Cas9 without sgRNA			Single Cas9n with sgRNA			P. value	P. value
	N.O.	Location	Indels	Total	Indels%	Indels	Total	Indels%	Indels	Total	Indels%	(Cas9 vs con)	(Cas9n vs con)
sgRNA45	ON	chr25-40631939-40632439	1652	3854	42.86455	74	5744	1.288300	83	7328	1.132641	0.000	0.233
	OT1	chr18-55380757-55381257	73	2851	2.560505	129	5967	2.161890	41	1684	2.434679	0.137	0.278
	OT2	chr25-2632321-2632821	8	1521	0.525969	14	2063	0.678623	16	2752	0.581395	0.363	0.402
	OT3	chr26-16100195-16100695	83	7963	1.042320	72	9642	0.746733	49	5681	0.862524	0.022	0.247
	OT4	chr29-12763711-12764211	43	3847	1.117754	62	5579	1.111310	35	3269	1.070663	0.526	0.475
	OT5	chr29-45518937-45519437	12	2784	0.431034	39	7849	0.496878	31	5736	0.540446	0.402	0.409
	OT6	chr15-54271885-54272385	4	958	0.417536	21	3673	0.571739	36	4846	0.742880	0.387	0.205
	OT7	chr17-42590109-42590609	64	7451	0.858945	117	14637	0.799344	98	11642	0.841779	0.350	0.378
	OT10	chr8-109757219-109757719	16	1689	0.947306	37	6839	0.541014	96	13827	0.694293	0.047	0.114
	OT11	chr5-62677592-62678092	45	6531	0.689021	78	10636	0.733358	85	12770	0.665622	0.405	0.294
	OT12	chr13-6938241-6938741	262	27708	0.945575	75	8736	0.858516	59	6115	0.964840	0.249	0.279
	OT13	chr13-77751338-77751838	94	6791	1.384184	59	3863	1.527310	37	1849	2.001081	0.304	0.117
	OT14	chr2-108449176-108449676	67	8620	0.777262	29	4582	0.632911	6	834	0.719424	0.206	0.458
	OT15	chr10-37931862-37932362	81	15435	0.524781	31	6522	0.475314	51	8421	0.605628	0.357	0.169
sgRNA20	ON	chr25-40631939-40632439	2072	6703	30.91153	121	11577	1.045175	75	5878	1.275944	0.000	0.098
	OT1	chr25-42863425-42863925	25	1783	1.402131	25	2271	1.100836	21	1552	1.353092	0.235	0.289
	OT2	chr8-67886580-67887080	7	619	1.130856	48	3749	1.280341	45	3863	1.164897	0.473	0.362
	OT4	chr15-46054766-46055266	31	1884	1.645435	48	7321	0.655648	121	16321	0.741376	0.000	0.261
	OT5	chr17-67139136-67139636	18	2307	0.780234	63	8654	0.727987	52	7513	0.692133	0.451	0.430
	OT6	chr18-32760919-32761419	174	10772	1.615298	94	6291	1.494198	61	4729	1.289913	0.291	0.206
	OT7	chr11-23615174-23615674	15	958	1.565762	72	5739	1.254573	121	8402	1.440133	0.256	0.195
	OT8	chr24-35983183-35983683	135	10073	1.340216	67	5372	1.247207	83	8615	0.963435	0.341	0.067
	OT9	chr13-51777151-51777651	15	1649	0.909642	48	6388	0.751408	79	9721	0.812673	0.303	0.367
	OT11	chr17-43449814-43450314	174	27938	0.622807	37	6421	0.576234	36	8942	0.402594	0.366	0.077
	OT12	chr23-18123965-18124465	145	11670	1.242502	84	8732	0.961978	67	6721	0.996875	0.005	0.416
	OT13	chr23-22687723-22688223	17	1628	1.044226	6	673	0.891530	8	862	0.928074	0.471	0.581
	OT14	chr9-43080420-43080920	8	932	0.858369	221	37531	0.588846	42	7821	0.537015	0.194	0.320
	OT15	chr28-24658945-24659445	47	6432	0.730721	32	4679	0.683906	34	6732	0.505050	0.430	0.101

			WT Cas9 with sgRNA			WT Cas9 without sgRNA			Single Cas9n with sgRNA			P. value	P. value
	N.O.	Location	Indels	Total	Indels%	Indels	Total	Indels%	Indels	Total	Indels%	(Cas9 vs con)	(Cas9n vs con)
**sgRNA22**	ON	chr25-40631939-40632439	367	1759	20.86412	28	3481	0.804366	37	5392	0.686201	0.000	0.303
	OT1	chr3-35217867-35218367	83	4992	1.662660	216	13804	1.564763	137	8726	1.570020	0.342	0.510
	OT2	chr4-68632846-68633346	48	3728	1.287553	67	5432	1.233431	30	2871	1.044932	0.445	0.259
	OT3	chr6-115453345-115453845	47	2781	1.690039	36	3852	0.934579	67	6539	1.024621	0.035	0.380
	OT4	chr17-41912018-41912518	216	7621	2.834273	43	3865	1.112548	63	5439	1.158301	0.000	0.460
	OT6	chr13-17174650-17175150	51	6075	0.839506	27	2886	0.935550	75	7632	0.982704	0.364	0.457
	OT7	chr2-132559396-132559896	62	2670	2.322097	361	13672	2.640433	96	3701	2.593893	0.189	0.461
	OT8	chr19-52727848-52728348	98	7282	1.345784	84	5636	1.490418	45	2864	1.571229	0.269	0.419
	OT9	chr14-68032489-68032989	542	15144	3.578975	54	2083	2.592414	128	4506	2.840656	0.010	0.314
	OT10	chr9-80334366-80334866	12	1753	0.684540	42	5420	0.774907	56	6597	0.848870	0.423	0.364
	OT11	chr4-55591291-55591791	70	5606	1.248662	113	7809	1.447048	53	3409	1.554708	0.184	0.363
	OT12	chr12-84971585-84972085	49	2390	2.050209	125	5670	2.204585	84	4055	2.071516	0.366	0.355
	OT13	chr5-121981533-121982033	136	8345	1.629718	34	1387	2.451333	127	4890	2.597137	0.024	0.424
	OT15	chr23-18173144-18173644	238	23409	1.016702	87	9631	0.903332	112	11862	0.944191	0.187	0.405
**sgRNA2**	ON	chr25-40631939-40632439	79	2396	3.297161	65	4561	1.425126	62	3972	1.560926	0.000	0.334
	OT1	chr4-1192145-1192645	273	13470	2.026726	93	5004	1.858513	73	4621	1.579744	0.252	0.168
	OT2	chr3-103129008-103129508	185	5678	3.258189	104	4743	2.192705	45	1806	2.491694	0.001	0.261
	OT3	chr4-25172348-25172848	32	3467	0.922988	58	6732	0.861556	41	4382	0.935645	0.420	0.381
	OT5	chr24-45736136-45736636	125	9578	1.305074	41	3628	1.130099	54	5672	0.952045	0.236	0.233
	OT6	chr26-33309894-33310394	90	3618	2.487562	16	1732	0.923787	6	763	0.786369	0.000	0.470
	OT7	chr5-111340663-111341163	29	4935	0.587639	214	15097	1.417500	129	9555	1.350078	0.000	0.350
	OT8	chr10-45996655-45997155	24	5382	0.445930	11	2682	0.410141	17	3571	0.476057	0.488	0.426
	OT9	chr9-3820599-3821099	36	4561	0.789300	18	3655	0.492476	33	7139	0.462249	0.063	0.473
	OT10	chr9-99399395-99399895	34	7195	0.472550	24	6422	0.373715	42	8226	0.510576	0.226	0.135
	OT11	chr3-124395553-124396053	174	13427	1.295896	73	9726	0.750565	117	6321	0.395507	0.311	0.477
	OT13	chr1-108472728-108473228	5	842	0.593824	24	5732	0.418702	25	4775	0.732984	0.491	0.498
	OT14	chr27-38949057-38949557	45	6430	0.699844	64	8935	0.716284	35	5632	0.852272	0.008	0.430
	OT15	chr18-41337881-41338381	74	4562	1.622095	23	2508	0.917065	48	6321	0.395507	0.311	0.477

Table. S5 Summary of junction PCR results of puromycin-resistant colonies.

BFF cells	Donor plasmid 1/2 only[1]	WT Cas9 +donor plasmid 1/2	WT Cas9-sgR20 +donor plasmid 1	D10A Cas9-sgR20+ donor plasmid 1	WT Cas9-sgR45 +donor plasmid 2	D10A Cas9-sgR45+ donor plasmid 2	D10A Cas9-sgR2 + D10A Cas9-sgR19+ donor plasmid 1	D10A Cas9-sgR16+ D10A Cas9-sgR34+ donor plasmid 2
Puromycin^R colonies	5/3	13/9	169	372	165	381	127	143
5' Junction PCR$^+$ colonies	0	0	47 (27.81)	51 (13.71)	62 (37.58)	73 (19.16)	11 (8.66)	16 (11.19)
3' Junction PCR$^+$ colonies	0	0	43 (25.44)	48 (12.90)	57 (34.55)	69 (18.11)	9 (7.09)	13 (9.09)
Suitable for SCNT[2]	0	0	38	47	50	63	9	12

[1] The only difference in donor plasmid 1 and 2 is right homology arms sequence for corresponding target sites.

[2] Heterozygous colonies with normal karyotype, compact spindle-like cell morphology, and rapid growth were defined as suitable for SCNT.

Table. S6 Constitution of the positive cell colonies generated by different types of Cas9 at the target site 45.

Group	Positive colonies	Heterozygous colonies	Homozygous colonies	Random insertion[1]	Indels in the allele [2]
**WT Cas9**	57	55	0	2	5
**Single Cas9n**	69	69	0	0	0

[1] This content mean the positive colonies which contain another transgene cassette insertion apart from the target site 45.

[2] Indels in the allele were detected by PCR with the primers 5j F and 3j R which were also used for junction PCR.

Table. S7 Sequences of primers for Real-Time PCR.

Gene name	Primer	Sequence (5′−3′)	Product length
FSCN1	Forward	ATCGGAGGATTATTCTGCGTG	209 bp
	Reverse	ATAGTTGGAGCGGTTGGCA	
ACTB	Forward	CTGCGGCATTCACGAAACT	268 bp
	Reverse	CTGCTTGCTGATCCACATCTG	
FBXL18	Forward	AGCACGGACCTGGTTCTGAA	428 bp
	Reverse	ACGCCATACGAGGGTGTGTA	
GAPDH	Forward	CAAGTTCAACGGCACAGTCAA	368 bp
	Reverse	TGGTCATAAGTCCCTCCACGAT	
